# Synthesis of P-Modified DNA from Boranophosphate DNA as a Precursor via Acyl Phosphite Intermediates

**DOI:** 10.1021/acs.joc.3c00659

**Published:** 2023-07-18

**Authors:** Yuhei Takahashi, Kiyoshi Kakuta, Yukichi Namioka, Ayumi Igarashi, Taiichi Sakamoto, Rintaro Iwata Hara, Kazuki Sato, Takeshi Wada

**Affiliations:** † Department of Medicinal and Life Sciences, Faculty of Pharmaceutical Sciences, Tokyo University of Science, 2641 Yamazaki, Noda, Chiba 278-8510, Japan; ‡ Department of Medical Genome Sciences, Graduate School of Frontier Sciences, The University of Tokyo, Kashiwa, Chiba 277-8562, Japan; § Department of Life Science, Chiba Institute of Technology, Graduate School of Advanced Engineering, Chiba 275-0016, Japan; ∥ Department of Neurology and Neurological Science, Graduate School of Medicinal and Dental Sciences, Tokyo Medical and Dental University, Tokyo 113-8519, Japan

## Abstract

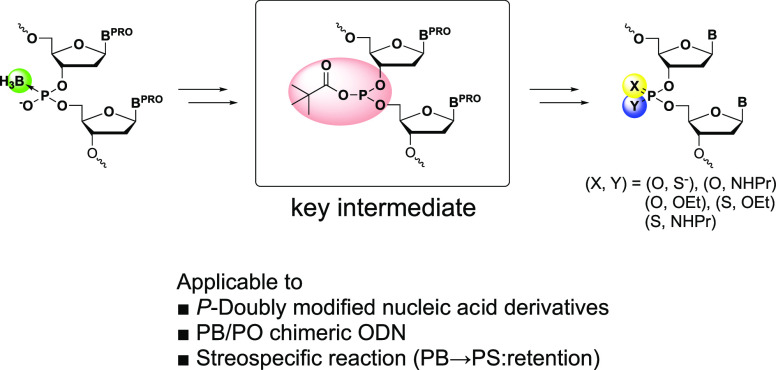

In this study, we successfully synthesized several kinds of *P*-modified nucleic acids from boranophosphate DNAs via an acyl phosphite intermediate in solution and on a solid support. In the solution-phase synthesis, phosphorothioate diester, phosphotriester, and phosphoramidate diester were synthesized in a one-pot reaction from boranophosphodiester via the conversion of an acyl phosphite as a key intermediate. In addition, doubly *P*-modified nucleic acid derivatives which were difficult to synthesize by the phosphoramidite and *H*-phosphonate methods were also obtained by the conversion reaction. In the solid-phase synthesis, a boranophosphate derivative was synthesized on a solid support using the *H*-boranophosphonate method. Then, an acyl phosphite intermediate was formed by treatment with pivaloyl chloride in pyridine, followed by appropriate transformations to obtain the *P*-modified derivatives such as phosphotriester and phosphorothioate diester. Notably, it was suggested that the conversion reaction of a boranophosphate to a phosphorothioate diester proceeded with retention of the stereochemistry of the phosphorous center. In addition, a phosphorothioate/phosphate chimeric dodecamer was successfully synthesized from a boranophosphate/phosphate chimeric dodecamer using the same strategy. Therefore, boranophosphate derivatives are versatile precursors for the synthesis of *P*-modified DNA, including chimeric derivatives.

## Introduction

A wide range of *P*-modified oligodeoxyribonucleotides (ODNs) have been developed because the introduction of *P*-modification into antisense oligonucleotides (ASOs) improves their physicochemical and biological properties. A phosphorothioate (PS) backbone, in which one of the nonbridging oxygen atoms is replaced with a sulfur atom, is the most commonly used chemical modification applied to ASOs owing to its high nuclease resistance, preferable pharmacokinetics, and ease of accessibility.^1,2^ However, some PS ODNs are cytotoxic and trigger undesired side effects, creating a major obstacle for clinical trials.^3,4^ Recently, it was revealed that replacing part of the PS linkages of the ASOs with several types of *P*-modifications, such as methoxypropylphosphonate^5^ and mesylphosphoramidate,^6^ could secure both the safety and efficacy of ASOs. Thus, there is a growing demand to investigate the properties of various *P*-modified ODNs, and a versatile method to synthesize them is needed to develop a more potent ASO. To date, the phosphoramidite^7^ and *H*-phosphonate^8^ methods have been widely used to synthesize *P*-modified ODNs. In the phosphoramidite method, *P*-modified derivatives are synthesized by the stepwise conversion reaction of phosphite triester intermediates after each condensation reaction. In contrast, a wider range of *P*-modified ODNs can be synthesized by the simultaneous transformation of internucleotidic *H*-phosphonate diesters in the final stage of the synthesis using the *H*-phosphonate method.^8^

**Scheme 1:**
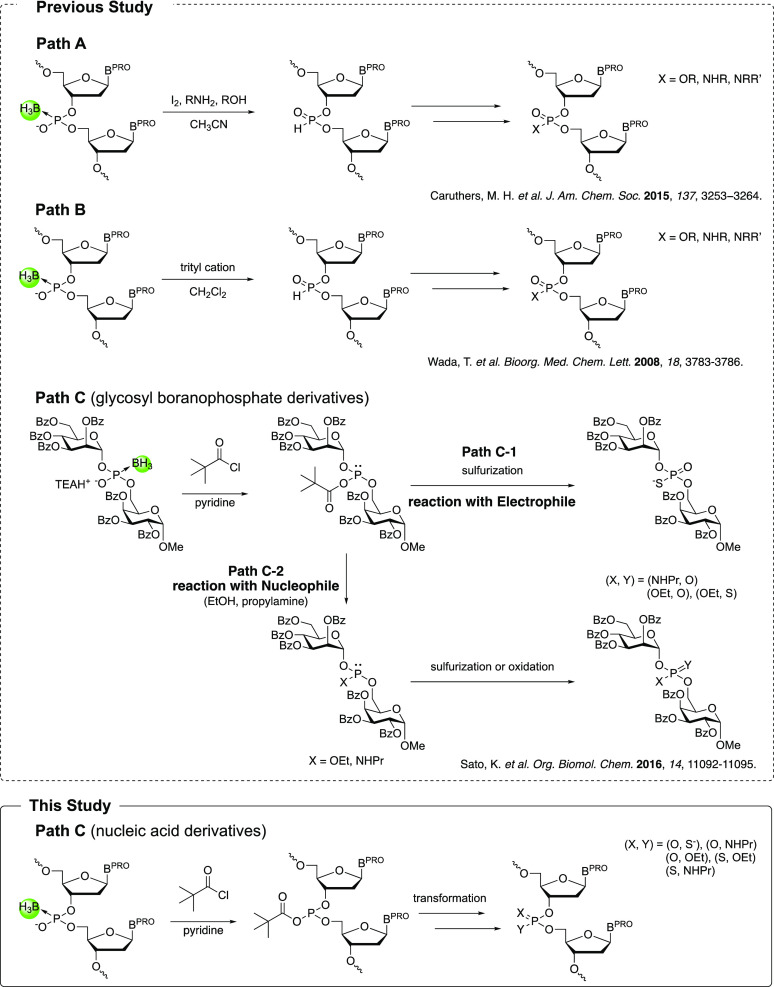
Previous Study and This Study of the Conversion Reaction of Boranophosphodiester

In addition to these synthetic methods for *P*-modified ODNs, our group^9^ and Caruthers and co-workers^10^ proposed another strategy to synthesize *P*-modified ODNs using boranophosphate (PB) derivatives, in which a nonbridging oxygen atom of a phosphate (PO) diester is replaced with a borano group. PB derivatives were developed by Shaw and co-workers^11^ and are alternative candidates for ASOs because PB derivatives offer higher nuclease resistance than their PS counterparts^12^ and exhibit low cytotoxicity.^13,14^ Therefore, several efficient synthetic methods have been developed.^12,15,16^ In addition, our group and Caruthers and co-workers focused on the PB derivatives as a synthetic precursor and tried to convert it to various *P*-modified ODNs. Caruthers and co-workers achieved the synthesis of a variety of *P*-modified ODNs through the reaction of PB derivatives with several nucleophiles, such as amine, in the presence of iodine (Scheme 1 Path A).^10^ This mechanism was rigorously investigated by Stawinski and co-workers.^17^ Briefly, a boranophosphodiester is activated by iodine to produce an iodoboranophosphate and then allowed to react with an amine to yield aminoboranophosphate derivatives. The obtained aminoboranophosphate is dissociated into an *H*-phosphonate diester as a key intermediate. The *H*-phosphonate diester is converted to a phosphoroiodidate by iodine, and the corresponding phosphoramidate can be synthesized via substitution by an amine as a nucleophile.

Meanwhile, it has been reported that boranophosphodiester is unstable in the presence of a trityl cation and is converted to an *H*-phosphonate diester.^18^ From a different perspective, the borano group can be regarded as a protecting group for *H*-phosphonate linkages.^19^ Based on this concept, we developed a new method to synthesize *P*-modified deoxyribonucleotides.^9^ Briefly, a PB diester is synthesized on a solid support and then converted into the *H*-phosphonate diester, followed by the conversion of *H*-phosphonate linkages to several types of *P*-modified diester (Scheme 1 Path B). In addition to these reactions, *P*-modified derivatives are obtained under mild basic conditions from a boranophosphodiester via a key intermediate, acyl phosphite, in a study of glycosyl phosphate derivatives (Scheme 1 Path C).^20^ First, a boranophosphodiester is treated with pivaloyl chloride (PivCl) in the presence of pyridine to produce a mixed anhydride, and then the borano group is removed by pyridine to obtain an acyl phosphite. Then, the obtained acyl phosphite derivative is transformed to obtain *P*-modified derivatives. For example, the acyl phosphite derivative acts as a nucleophile with a sulfurizing reagent, such as 3-phenyl 1,2,4-dithiazoline-5-one (POS), to eventually yield a PS diester (Scheme 1 Path C-1). In contrast, acyl phosphite derivatives react as electrophiles with alcohol and amine to yield a phosphite triester and phosphoramidite. These intermediates are then transformed into a phosphotriester, phosphoramidate, and phosphorothioate triester, respectively, by oxidation or sulfurization (Scheme 1 Path C-2). In this study, the transformation reaction of a boranophosphodiester via an acyl phosphite as the key intermediate was applied to both solution and solid-phase synthesis of ODNs. In addition, this transformation reaction was applied to PB/PO chimeric ODNs to synthesize PO and *P*-modified chimeric ODNs.

## Results and Discussion

### ^31^P NMR Analysis for the Formation of an Acyl Phosphite

**Scheme 2:**
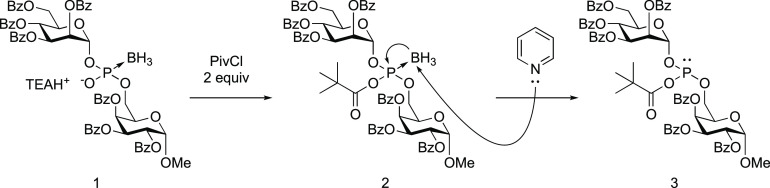
Proposed Mechanism for the Formation of an Acyl Phosphite Derivative^20^

**Scheme 3:**
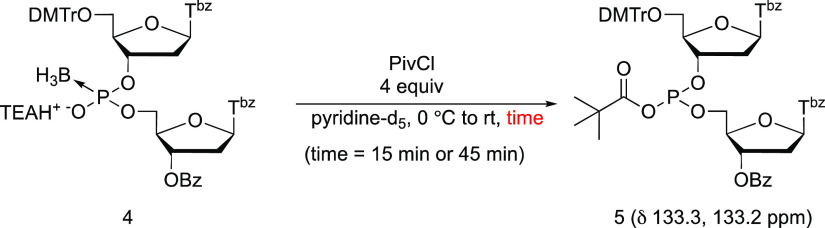
^31^P NMR Analysis for the Formation of an Acyl Phosphite

In the conversion reaction of glycosyl boranophosphates (**1**) using PivCl and pyridine, the reaction is expected to proceed according to the following mechanism (Scheme 2).^20^ First, the boranophosphodiester (**1**) reacts with PivCl to produce a mixed acid anhydride (**2**); then, with pyridine as a nucleophile, deboronation of the mixed acid anhydride occurs to form an acyl phosphite derivative (**3**) as a key intermediate. Thus, we analyzed the reaction mechanism by ^31^P nuclear magnetic resonance (NMR) to confirm that the reaction also occurs by this mechanism with a nucleic acid derivative. ^31^P NMR analysis was conducted for 15 and 45 min after the addition of 4 equivalents of PivCl to the solution of a boranophosphodiester (**4**) in pyridine-d_5_ (Scheme 3). (The synthetic procedure of the boranophosphodiester (**4**) is described in Supporting Information, and it should be noted that a thymidine boranophosphotriester monomer was protected by the benzoyl group at the *N*^3^ position of thymine to avoid side reactions on the nucleobase.^21^) The signals at 133.3 and 133.2 ppm were predominant, indicating the formation of diastereomers of an acyl phosphite (**5**).^22^ However, a small amount of an *H*-phosphonate diester was also observed as a byproduct in the formation of acyl phosphite probably due to hydrolysis of acyl phosphite (δ 10.4, 8.9 ppm, Figure S1).

### Reaction between an Acyl Phosphite and Electrophile

**Scheme 4:**
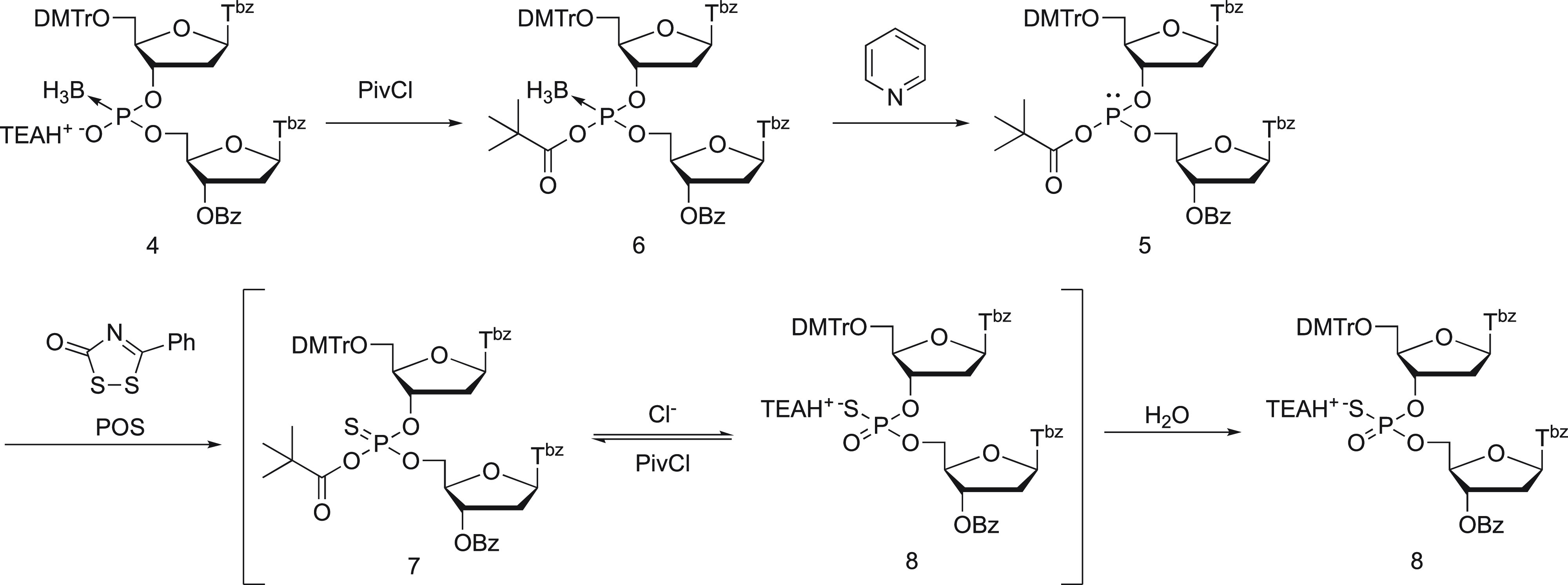
Plausible Mechanism for Dithymidine Phosphorothioate Diester Formation

**Scheme 5:**
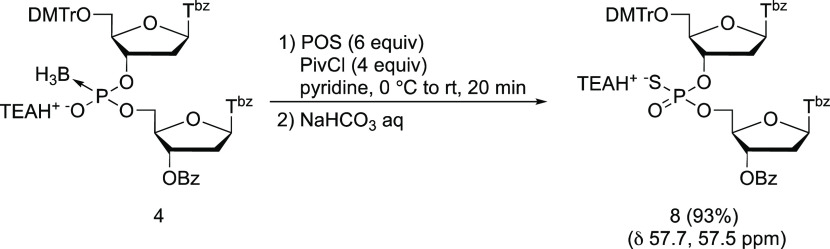
One-Pot Synthesis of Dithymidine Phosphorothioate Diester

Next, we attempted the conversion reaction of the acyl phosphite to its PS counterpart with a sulfurizing reagent, POS. PivCl was added to a mixture containing the boranophosphodiester (**4**) in pyridine-d_5_ in the presence of POS, and the reaction was analyzed by ^31^P NMR after 30 and 60 min. As a result, four signals were observed in the ^31^P NMR spectra (δ 60.7, 60.4, 58.0, 57.4 ppm) (Figure S2). The lower (δ 60.7, 60.4 ppm) and upper (δ 58.0, 57.4 ppm) field signals observed at the beginning likely arose from the diastereomers of the mixed anhydride derivative (**7**) and the PS diester (**8**), respectively. Thereafter, a saturated NaHCO_3_ aqueous solution (100 μL) was added to the solution, resulting in two signals (δ 57.2, 56.7 ppm) corresponding to compound **8**. The existence of the PS diester (**8**) before the addition of an aqueous solution was unexpected because PivCl would act as a dehydrating reagent. To prove the mechanism, after the acyl phosphite (**5**) was sulfurized, 10 equivalents of PivCl were added to the reaction mixture (Figure S3). Despite the presence of an excess amount of PivCl, signals corresponding to both the mixed anhydride (**7**) and the PS diester (**8**) were observed. From these results, it was indicated that the mixed anhydride derivative (**7**) and PS diester (**8**) were in an equilibrium state (Scheme 4). A one-pot reaction was then carried out to produce a dithymidine PS diester (Scheme 5), and a PS diester (**8**) was isolated with a yield of 93% without notable side reactions.

### Reaction between an Acyl Phosphite and a Nucleophile Such as an Alcohol or Amine

**Scheme 6:**
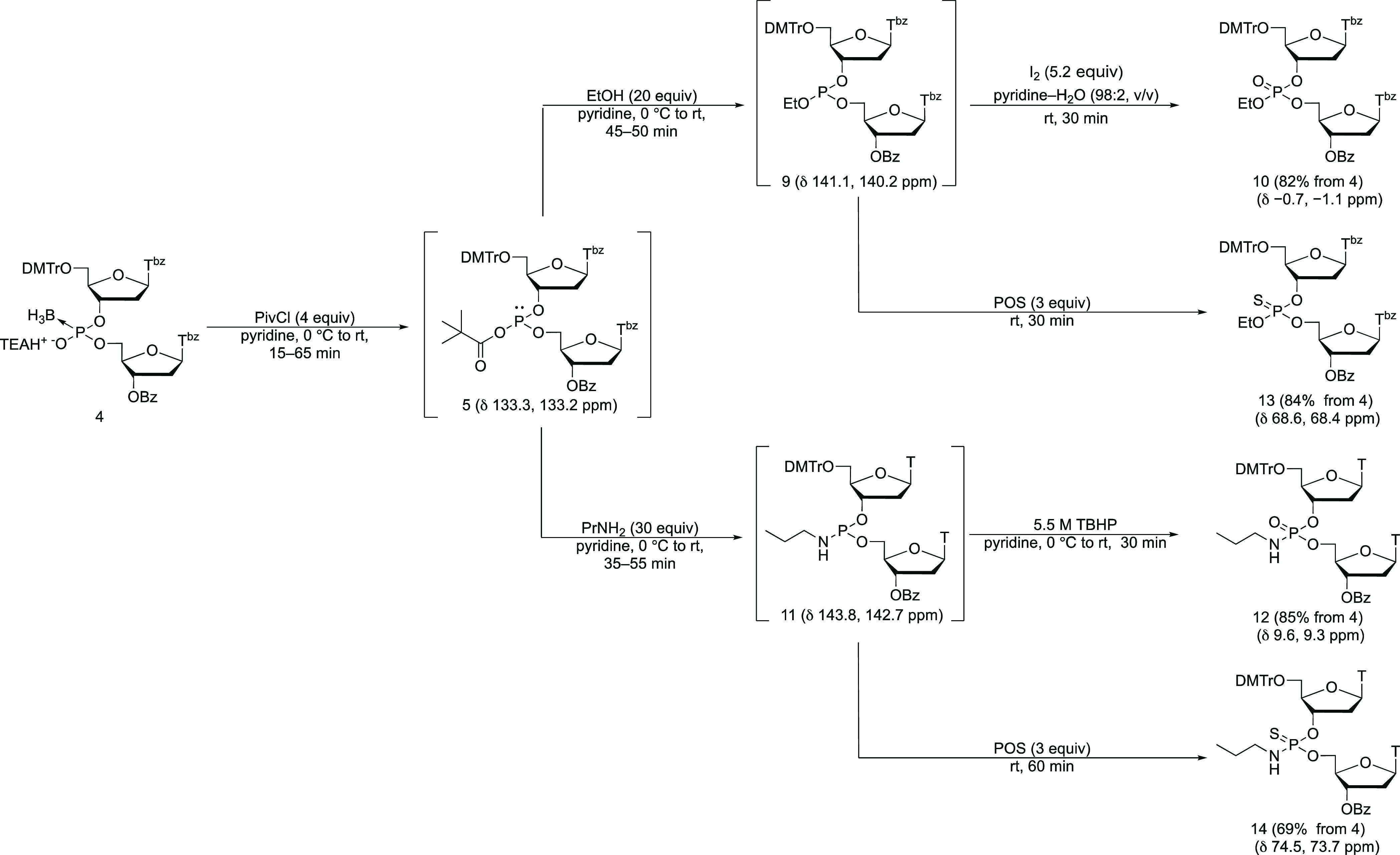
One-Pot Synthesis of *P*-Modified Nucleic Acid Derivatives

Subsequently, we attempted a conversion reaction of the acyl phosphite with a nucleophile. PivCl was allowed to react with the boranophosphodiester (**4**) in pyridine for 25 min, followed by the addition of 20 equiv of ethanol (EtOH), and the reaction mixture was analyzed 60 min after the addition of EtOH by ^31^P NMR. Two signals were mainly observed at 141.1 and 140.2 ppm, suggesting the formation of a phosphite triester intermediate. In addition to that, unidentified signals around 0 ppm were also observed (Figure S4). Then, this reaction was carried out as a one-pot reaction to convert the boranophosphodiester to its phosphotriester counterpart (Scheme 6). A solution of I_2_/pyridine–H_2_O (98:2, v/v) was used as an oxidizing reagent of the phosphite triester intermediate (**9**) to phosphotriester (**10**). Under this conversion reaction, the desired phosphotriester (**10**) was isolated with a yield of 82%.

Next, we investigated the conversion reaction to a phosphoramidate diester from the boranophosphodiester. After the formation of the acyl phosphite following the procedure described above, propylamine was added to the reaction mixture, which was analyzed by ^31^P NMR after 15 min. Two signals were observed at 143.8 and 142.7 ppm, indicating the formation of a phosphoramidite intermediate (**11**). In addition to these signals, unidentified signals around 0 ppm were also observed (Figure S5). Then, a one-pot reaction to obtain the phosphotriester (**12**) was conducted using *t*-BuOOH as an oxidizing reagent for the phosphoramidite intermediate (**11**) (Scheme 6). The desired phosphoramidate (**12**) was isolated with a yield of 85%. It must be noted that the benzoyl groups on the *N*^3^ position of thymidine were removed upon treatment with propylamine.

### Synthesis of Doubly *P*-Modified Nucleic Acid Derivatives

Next, we tried to synthesize doubly *P*-modified nucleic acid derivatives. A phosphorothioate triester and a phosphorothioamidate were chosen as synthetic targets. In terms of phosphorothioate triester, a phosphite triester intermediate was synthesized as mentioned above, followed by adding POS as a sulfurizing reagent of the phosphite triester (Scheme 6). The desired phosphorothioate triester (**13**) was isolated with a yield of 84%.

For the synthesis of a phosphorothioamidate, the oxidation step for the synthesis of the phosphoramidate (**12**) was replaced with sulfurization by POS (Scheme 6). The ^31^P NMR analysis of this reaction indicated the product (**14**) was obtained in a 90% NMR yield (Figure S6). However, the separation of byproducts derived from POS and/or propylamine was troublesome, and multiple silica gel column chromatography and reprecipitation were conducted to isolate the product, resulting in a moderate yield (69%).

As described above, acyl phosphite is a versatile intermediate that reacts swiftly with both an electrophile and a nucleophile to give *P*-modified nucleic acid derivatives. Particularly, it afforded doubly *P*-modified analogs, whose synthesis is challenged by widely used phosphoramidite and *H*-phosphonate methods.

### Solid-Phase Synthesis of a Dinucleoside Phosphotriester

To expand the utility of the transformation reactions, we applied these transformation reactions to the solid-phase synthesis of *P*-modified ODNs. A PB derivative was synthesized by the *H*-boranophosphonate method.^15^ In brief, an *H*-boranophosphonate monomer unit (**16**) containing the characteristic H–P → BH_3_ group is condensed with a 5′-OH using a 1,3-dimethyl-2-(3-nitro-1,2,4-triazol-1-yl)-2-pyrrolidin-1-yl-1,3,2-diazaphospholidinium hexafluorophosphate (MNTP)^23^ to form an *H*-boranophosphonate diester linkage followed by a detritylation step without transformation of the resultant internucleotide linkages. It should be noted that thymine base was protected by acyl-protecting groups to suppress the side reaction caused by phosphonium-type condensing reagents bearing 3-nitro-1,2,4-triazol as a leaving group.^21^ These two steps are repeated until the desired length is achieved. Then, all internucleotidic *H*-boranophosphonate diesters are oxidized into boranophosphodiesters by treatment with CCl_4_ and water in the presence of a base, followed by the removal of the protecting groups of nucleobases and release from the solid support to obtain PB derivatives.^15^ In this study, after a PB derivative was synthesized on a solid support by the *H*-boranophosphonate method, the resultant PB was converted to several types of *P*-modified derivatives.

**Scheme 7:**
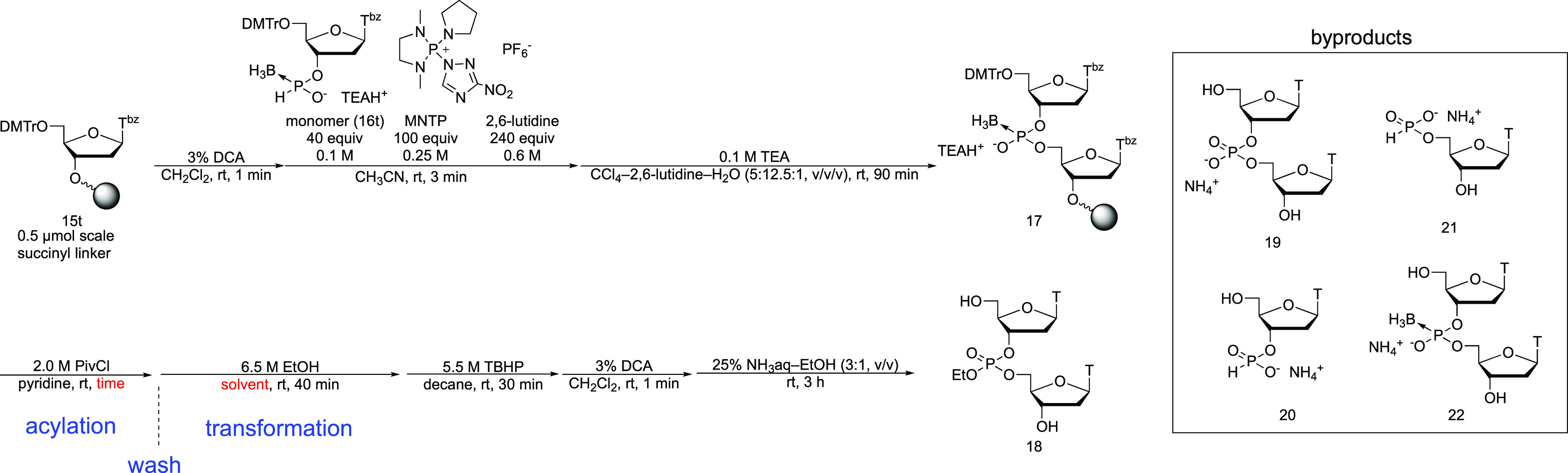
Solid-Phase Synthesis of Dithymidylate Phosphotriester

First, we attempted to synthesize a dinucleoside phosphotriester on a solid support using a transformation reaction with EtOH. As shown in Scheme 7, a PB derivative (**17**) was synthesized on a highly cross-linked polystyrene (HCP)^24^ solid support using the *H*-boranophosphonate method, followed by the formation of the acyl phosphite using 2.0 M PivCl in pyridine. Then, the transformation to phosphite triesters using EtOH, oxidation with *t*-BuOOH, deprotection of the 5′-*O*-dimethyoxytrytl (DMTr) group, removal of the protecting groups from the nucleobases, and cleavage of the linker by treatment with a mixture of concentrated aqueous NH_3_ and EtOH yielded the dinucleoside phosphotriester (**18**). The crude mixture was analyzed using reversed-phase high-performance liquid chromatography (RP-HPLC, Figure S7). It was worth noting that after the acyl phosphite was formed, it was washed with a reaction solution containing dry EtOH under an Ar atmosphere to prevent hydrolysis of the acyl phosphite. The results are shown in Table 1. In entry 1, the acylation reaction was conducted for 5 min, and the RP-HPLC analysis showed that the desired phosphotriester was obtained, but there were several peaks corresponding to thymidine, phosphodiester (**19**), *H*-phosphonate monoester (**20** and/or **21**), and PB diester (**22**), as judged by electrospray ionization mass spectrometry (ESI-MS). The presence of the boranophosphonate diester indicated that the conversion of the PB diester to a mixed anhydride derivative was incomplete for 5 min. Thus, the reaction conditions for the formation of the acyl phosphite and the phosphite triester in this conversion reaction were reinvestigated. To begin with, the effect of the reaction time (entries 1–3) on the formation of the acyl phosphite diester was examined. The extension of the reaction time from 15 to 60 min resulted in the disappearance of the peak corresponding to PB diester (**22**). In addition, the peak areas of *H*-phosphonate monoester (**20** and/or **21**) and thymidine also decreased (Figure S7, entries 2, 3). A PB diester is known to be converted into a *H*-phosphonate diester under the conditions for the removal of the DMTr group in the absence of a cation scavenger, as mentioned above.^18^ Therefore, byproducts, such as the *H*-phosphonate monoester (**20** and/or **21**) and thymidine, were partially formed by hydrolysis of the *H*-phosphonate diester, which was derived from the unreacted PB diester (**22**), under ammonia treatment. To take this into consideration, the conversion of the PB diester to the acyl phosphite derivative was suggested to be insufficient, even at 15 min. Thus, entry 3 was chosen as the optimal conditions for the formation of the acyl phosphite.

**Table 1:** Solid-Phase Synthesis for Dithymidylate Phosphotriester

entry	acylation conditions	wash and transformation conditions	HPLC yield of **18** (%)^a^
	time (min)		
1	5	6.5 M EtOH/pyridine	19
2	15	6.5 M EtOH/pyridine	72
3	60	6.5 M EtOH/pyridine	79
4	60	6.5 M EtOH/2,6-lutidine	55
5	60	6.5 M EtOH/1.0 M DIPEA in CH_3_CN	75
6	60	6.5 M EtOH/1.0 M DMAN in CH_3_CN	80
7^b^	60	6.5 M EtOH/1.0 M DMAN in CH_3_CN	79
8^b^^,^^c^	60	6.5 M EtOH/1.0 M DMAN in CH_3_CN	84
9^b^^,^^d^	60	6.5 M EtOH/1.0 M DMAN in CH_3_CN	54

a Determined by RP-HPLC: area ratio of **18**/(**18** + **19** + **20** + **21** + **22** + T).

b Washed with CH_3_CN between conversion to phosphite triester and oxidation using *t*-BuOOH.

c Treated with concentrated aqueous NH_3_aq–EtOH (3:1, v/v) at room temperature for 1 h.

d Treated with concentrated aqueous NH_3_aq–EtOH (3:1, v/v) at 50 °C for 12 h.

As a next step, the base used for the formation of phosphite triester (**18**) was studied in entries 3–6. Among pyridine, 2,6-lutidine, *i*Pr_2_NEt (DIPEA), and 1,8-bis(dimethylamino)naphthalene (DMAN), DMAN provided the best yield of the desired phosphotriester (80% HPLC yield). Thus, the conditions in entry 6 were established as the optimized conditions for the solid-phase synthesis of the phosphotriester (**18**). Finally, we investigated the stability of the dinucleoside phosphotriester under ammonia treatment. After the phosphotriester (**18**) was synthesized on the solid support using the optimized conditions, the solid support was divided into two and subjected to different ammonia treatment conditions (entry 8: room temperature, 1 h; entry 9: 50 °C, 12 h). The crude mixture obtained under each condition was analyzed by RP-HPLC (Table 1). When the phosphotriester (**18**) was treated with concentrated aqueous NH_3_aq–EtOH (3:1, v/v) on a solid support at room temperature for 1 h, the HPLC yield of the phosphotriester (**18**) improved from 79% (entry 7, 3 h) to 84% (entry 8, 1 h). However, harsher conditions (concentrated aqueous NH_3_aq–EtOH (3:1, v/v), 50 °C, 12 h) resulted in 54% HPLC yield (entry 9). The decomposition of phosphotriesters may occur with extended ammonia treatment time, even at room temperature, and ammonia treatment at a higher temperature causes significant degradation of the product. Under these circumstances, it was difficult to synthesize phosphotriesters using the standard protecting groups of all four nucleobases, and no further investigation into the synthesis of any other phosphotriester was conducted. However, the synthesis of phosphotriester is expected to be possible using a protecting group or linker that can be removed under mild conditions.

### Solid-Phase Synthesis of a Dinucleoside Phosphorothioate

**Figure 1:**
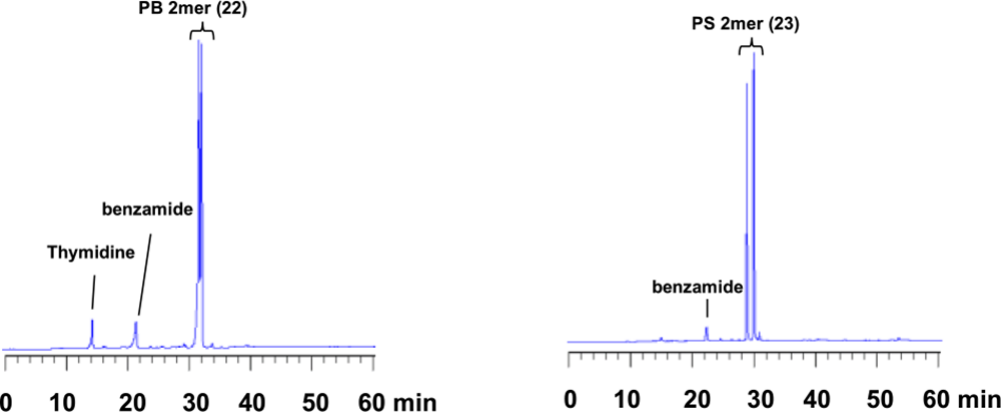
RP-HPLC profiles of PB dimer (**22**) (left) and PS dimer (**23**) (right).

**Scheme 8:**
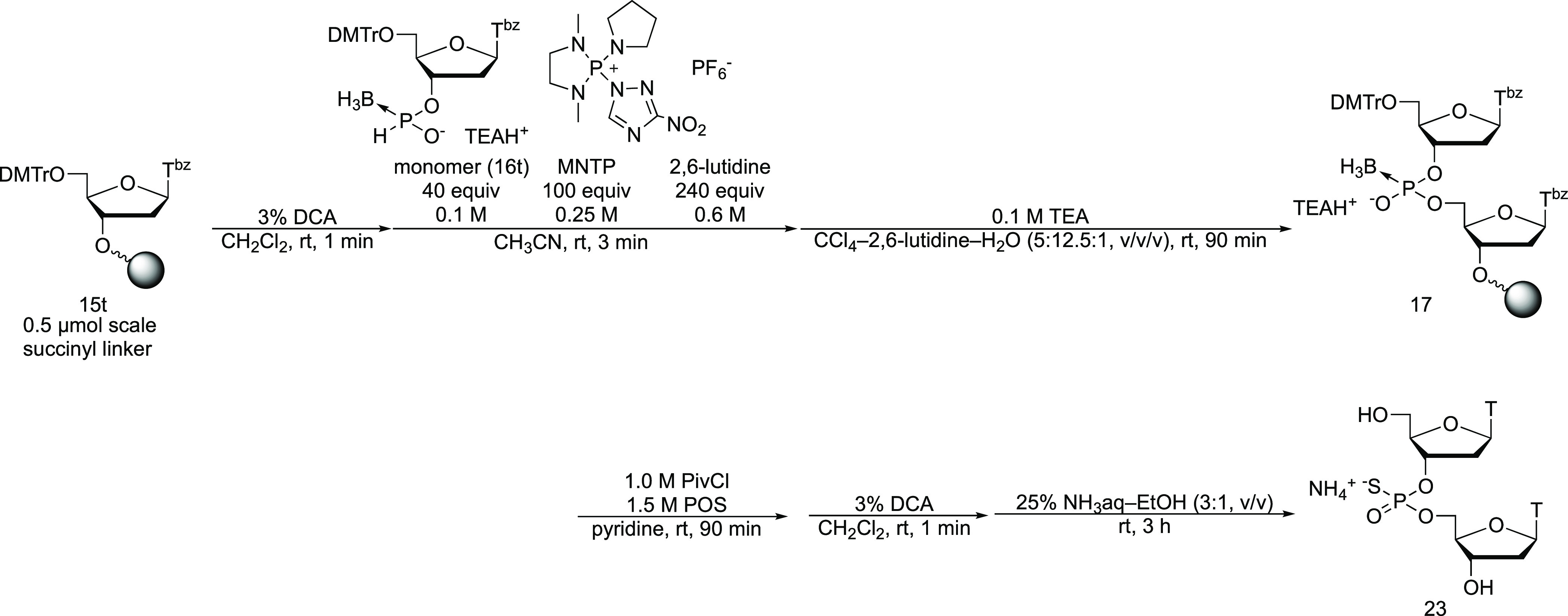
Solid-Phase Synthesis of Dithymidine Phosphorothioate Diester

Next, we synthesized a dinucleoside PS. The PB dimer with a 5′-DMTr group was synthesized on a solid support using the *H*-boranophosphonate method and then sulfurized by adding POS and a mixture of PivCl and pyridine to the reaction vessel to form a mixed anhydride of a PS and pivalic acid. This was followed by the deprotection of the DMTr group, removal of the protecting groups from the nucleobases, and release from the solid support yielded the dinucleoside PS (**23**) (Scheme 8). To evaluate the transformation efficiency, we also synthesized the PB dimer without the transformation reaction using POS and PivCl. It must be noted that the deprotection of the DMTr group was carried out before oxidation of the internucleotidic linkage with CCl_4_ because boranophosphodiester is unstable in the presence of the DMTr cation.^15^ The crude mixture obtained from each synthesis was analyzed by RP-HPLC. The dinucleoside PS (**23**) was obtained in over 99% HPLC yield, and the dinucleoside PB (**22**) was obtained in 96% HPLC yield (Figure 1). Therefore, the conversion reaction from PB derivatives to PS derivatives was verified quantitatively.

### Stereochemical Analysis of the Transformation Reaction

**Figure 2:**
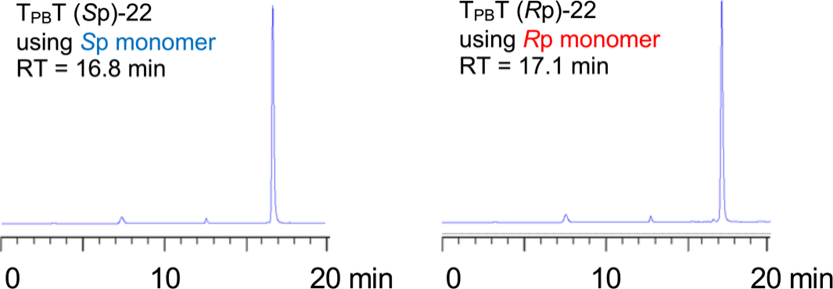
RP-HPLC profiles of (*S*p)-PB dimer ((*S*p)-**22**) synthesized by using (*S*p)-monomer ((*S*p)-**25**) (left) and (*R*p)-PB dimer ((*R*p)-**22**) synthesized by using (*R*p)-monomer ((*R*p)-**25**) (right).

**Scheme 9:**
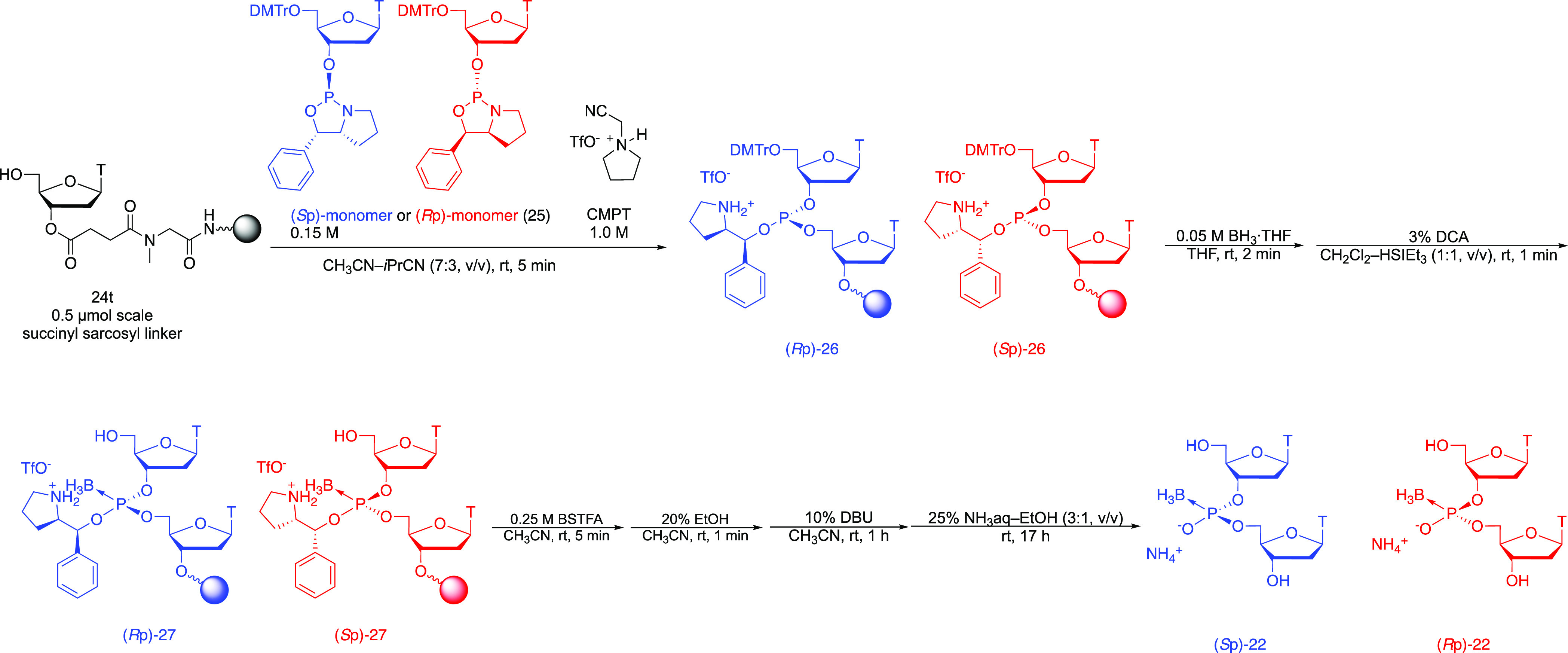
Stereocontrolled Synthesis of Boranophosphodiester

To investigate the stereochemistry of the reaction, the conversion reaction was applied to a stereopure boranophosphodiester. To obtain a stereopure boranophosphodiester, we used the oxazaphospholidine method.^25^ The oxazaphospholidine is a cyclic phosphoramidite derivative bearing a chiral auxiliary.^25^ Our group found that a bicyclic oxazaphospholidine derivative and a nonnucleophilic acidic activator enable a stereocontrolled synthesis of *P*-modified oligonucleotides.^26^ First, we carried out the determination of the stereochemistry of the boranophosphodiester obtained by the oxazaphospholidine method. A (*S*p)- or (*R*p)-oxazaphospholidine monomer (**25**) whose thymine base was unprotected^26^ was condensed with the 5′-OH on a solid support (**24t**) using *N*-cyanomethyl pyrrolidinium triflate (CMPT)^27^ as a nonnucleophilic acidic activator. The resultant phosphite triester was boronated followed by removal of the DMTr group on the 5′ position and capping of the liberated hydroxy group by a silylating reagent. After an *N*-TMS group was eliminated from a chiral auxiliary, the removal of the chiral auxiliary by treatment with DBU followed by an ammonia treatment afforded a stereopure boranophosphodiester (Scheme 9). Then, the crude mixture was analyzed and purified by RP-HPLC to determine the stereochemical purity of the boranophosphates (Figure 2). ^1^H NMR spectra of the purified products were compared with the ones reported by Li et al.,^28^ and it was found that the (*R*p) and (*S*p)-oxazaphospholidine monomers afforded (*R*p) and (*S*p)-dithymidine boranophosphates, respectively (Figures S8 and S9). In addition, the stereochemistry of the obtained boranophosphates was unambiguously determined by the 2D NOESY experiment with ^11^B decoupling (Figures S10 and S11). A cross-peak of protons of BH_3_ and the 5-methyl group of the 3′-downstream thymidine was clearly observed for the measurement of the presumptive *S*p isomer, whereas such a cross-peak was not observed for the measurement using the presumptive *R*p isomer. The molecular models of (*R*p) and (*S*p)-dithymidine boranophosphates were prepared from dithymidine phosphate, and it was indicated that the borano group of the (*S*p)-isomer occupied near the 5-methyl group of the 3′-downstream thymidine (Figure S11). Thus, NOESY spectra were in good agreement with the model structures.

**Figure 3:**
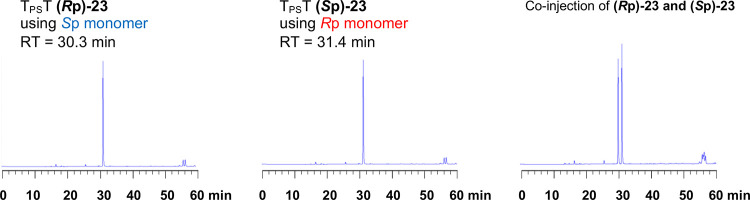
RP-HPLC profiles of (*R*p)-PS dimer ((*R*p)-**23**) from (*S*p)-**17** (left), (*S*p)-PS dimer ((*S*p)-**23**) from (*R*p)-**17** (center), and co-injection of (*R*p)-**23** and (*S*p)-**23** (right).

**Scheme 10:**
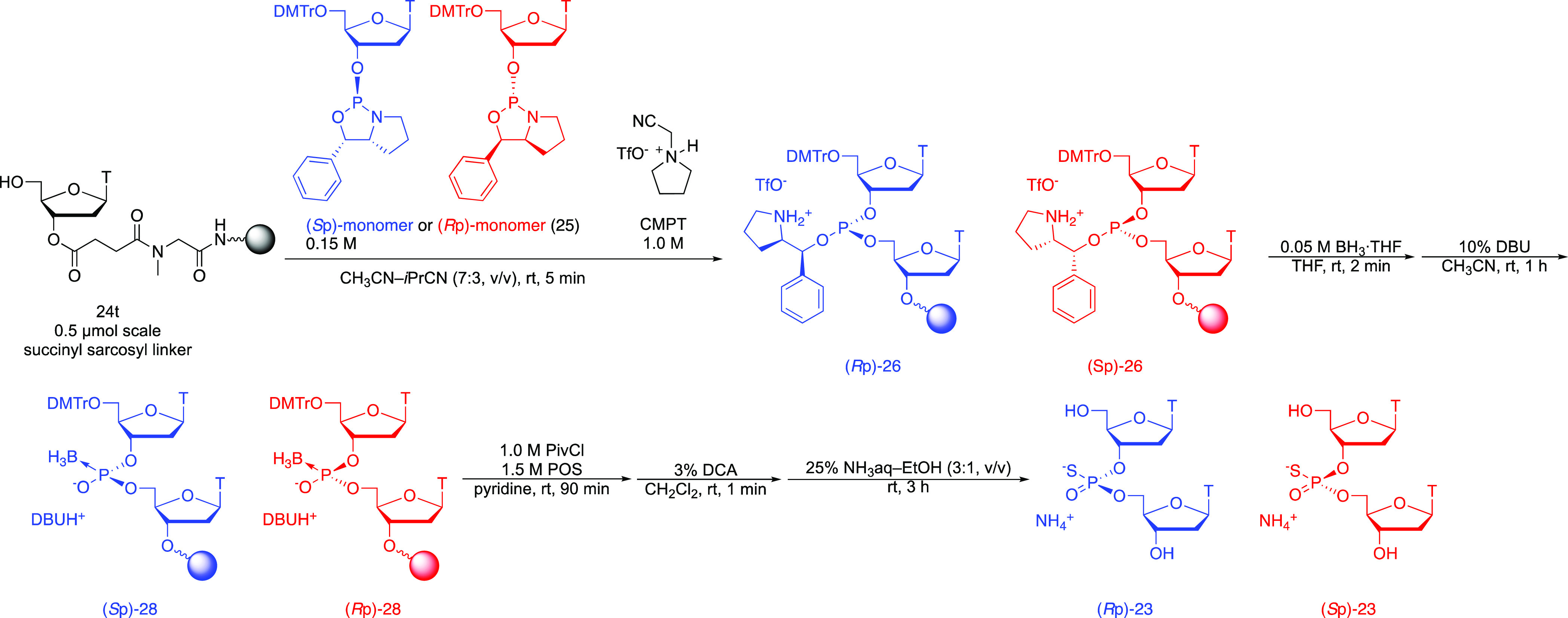
Stereocontrolled Synthesis of Phosphorothioate Diester

Next, the conversion reaction into the phosphorothioate was conducted. The stereopure (*S*p)- or (*R*p)-boranophosphodiesters (**17**) were synthesized on a solid support and converted to the phosphorothioate diester. It was followed by the deprotection of the DMTr group and release from a solid support to afford the phosphorothioate counterpart (Scheme 10). The crude mixtures were analyzed and purified by RP-HPLC. The RP-HPLC profiles suggested that there was only one peak corresponding to the stereopure phosphorothioate diester. Isolated products were co-injected into RP-HPLC, and these exhibited a different retention time (Figure 3). These results indicated that the conversion reaction underwent in a stereospecific manner. The products were analyzed by ^1^H and ^31^P NMR, and it was confirmed that a (*S*p)- and (*R*p)-boranophosphodiester gave a (*R*p)- and (*S*p)-phosphorothioate diester, respectively (Figures S12–S15).^26^ It should be noted that boranophosphate and phosphorothioate diastereomers with the identical absolute configuration of the chiral phosphorus atoms have opposite Prelog designations because the Prelog priority order of the atoms is S > O > B. From these results, the stereochemistry of the reaction was found to be overall retention.

### Solid-Phase Synthesis of PS ODNs and PS/PO Chimeric ODN

**Scheme 11:**
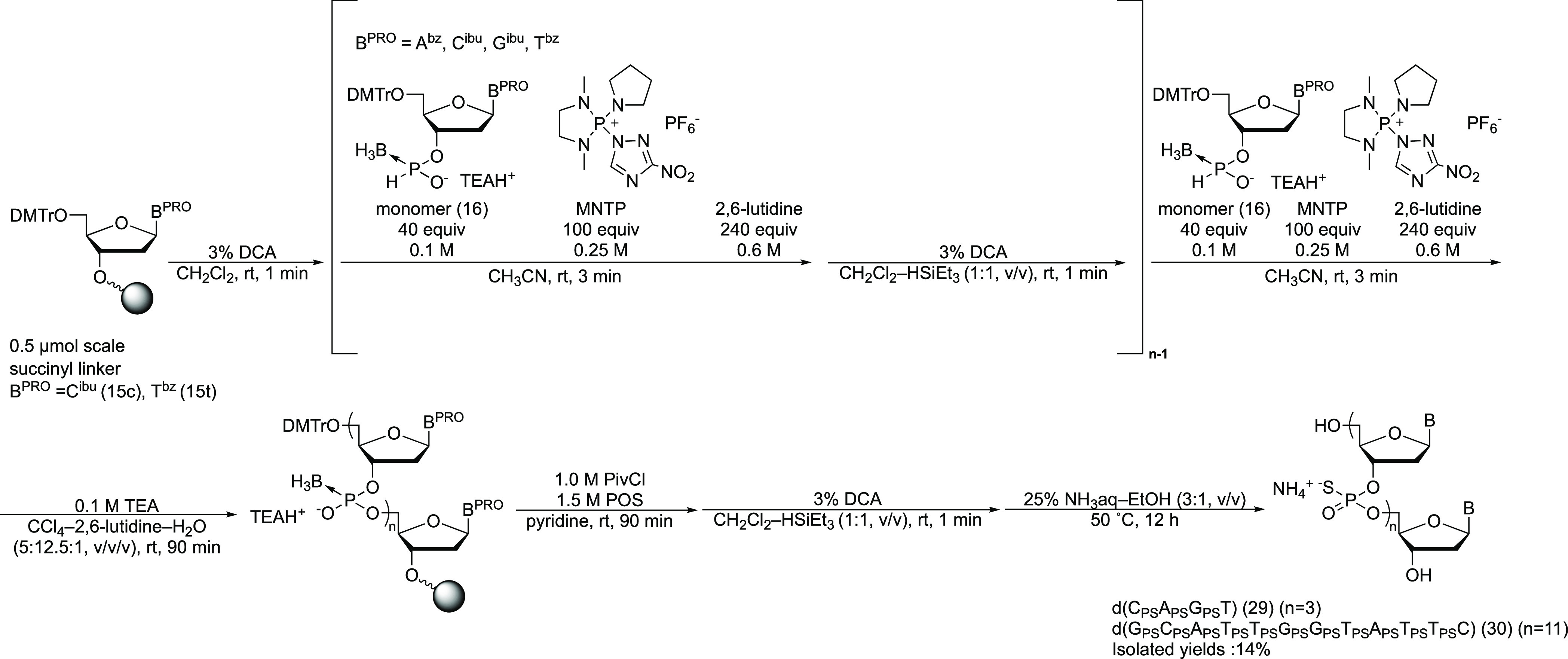
Solid-Phase Synthesis of PS ODNs

We attempted the conversion reaction to PS DNA tetramer containing four nucleobases (d(C_PS_A_PS_G_PS_T)) (**29**) from the PB counterpart. After the PB DNA tetramer was synthesized on a solid support, the tetramer was converted to the PS DNA using POS, PivCl, and pyridine, followed by the deprotection of the DMTr group, removal of the amino protecting groups, and release from the solid support (Scheme 11). The tetramers were confirmed to be the main products by RP-HPLC analysis of the reaction mixture (Figure S16). This result indicated that the conversion reaction was applicable without limitation to nucleobases.

These results prompted us to synthesize a PS DNA dodecamer. The synthetic target sequence comprised antisense sequences to apoB protein mRNA (d(G_PS_C_PS_A_PS_T_PS_T_PS_G_PS_G_PS_T_PS_A_PS_T_PS_T_PS_C)).^29^ The length of the strand was extended by repeated condensation of the *H*-boranophosphonate monomers and removal of the DMTr group at the 5′ position. After chain elongation was achieved, the internucleotidic *H*-boranophosphonate linkages were oxidized and the resultant PB linkages were converted to PS linkages by sulfurization using POS, PivCl, and pyridine. The PS DNA dodecamer was then obtained by removal of the DMTr group at the 5′ position, removal of the protecting groups at the nucleobases, and release from the solid support. After RP-HPLC purification, the PS dodecamer (**30**) was successfully isolated with a 14% yield (Figure S17). This result indicated that the PB dodecamer can be effectively converted to a PS dodecamer using this synthetic strategy.

**Scheme 12:**
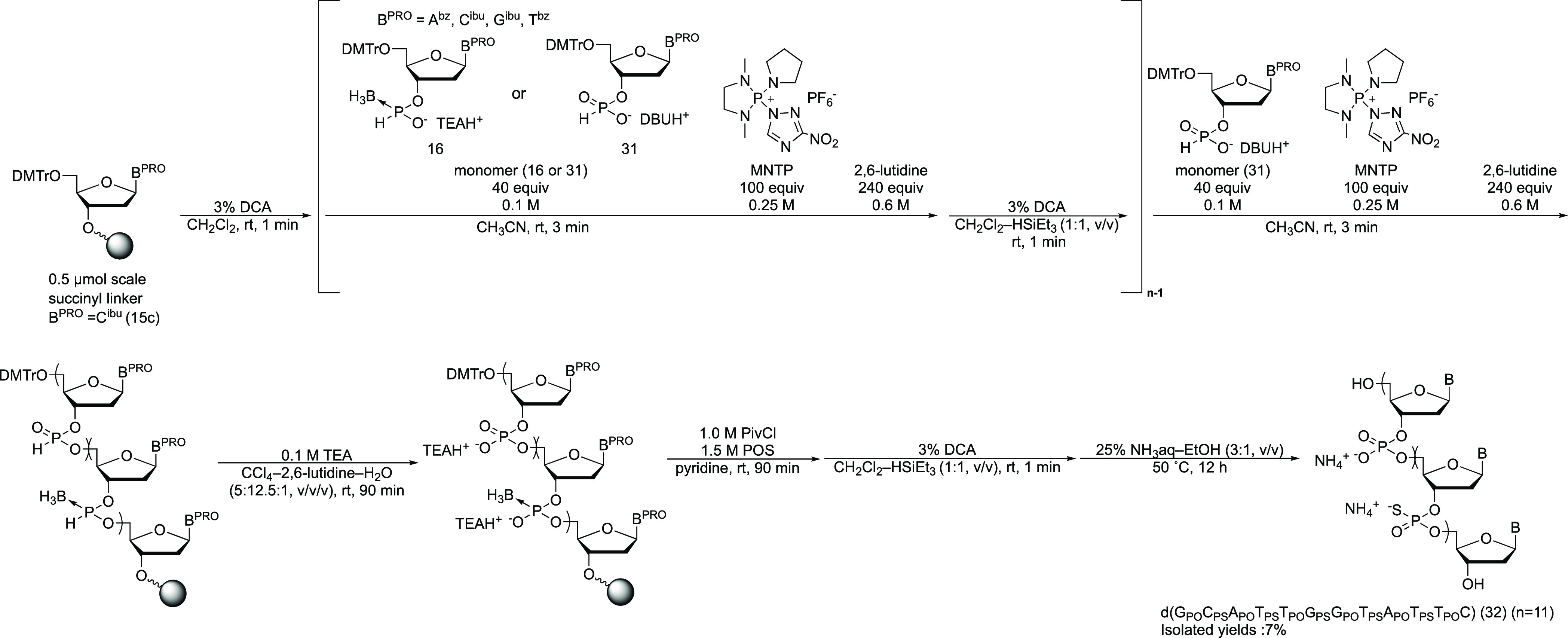
Solid-Phase Synthesis of a PS/PO Chimeric ODN from a PB/PO Counterpart

Finally, this transformation reaction was applied to the PB/PO dodecamer. We reported the solid-phase synthesis of PB/PO dodecamer by a combination of the *H*-boranophosphonate and *H*-phosphonate methods.^30^ Briefly, the length of the strand was extended by repeated condensations of an *H*-boranophosphonate (**16**) or *H*-phosphonate monomer (**31**)^30^ to produce *H*-boranophosphonate or *H*-phosphonate linkages, respectively, followed by the removal of the DMTr group at the 5′ position. After chain elongation, the internucleotidic *H*-boranophosphonate and *H*-phosphonate linkages were simultaneously oxidized to PB and PO linkages through treatment with CCl_4_ in the presence of water and a base. We speculated that the selective conversion of PB linkages to PS linkages produced a PS/PO chimeric ODN. Accordingly, after PB/PO chimeric dodecamer (d(G_PO_C_PB_A_PO_T_PB_T_PO_G_PB_G_PO_T_PB_A_PO_T_PB_T_PO_C)) was formed on a solid support, the solid support was treated with POS, PivCl, and pyridine, followed by the removal of the DMTr group at the 5′ position, removal of the protecting groups from the nucleobases, and release from the solid support (Scheme 12). The resultant mixture was analyzed and purified by RP-HPLC. The desired PS/PO dodecamer (d(G_PO_C_PS_A_PO_T_PS_T_PO_G_PS_G_PO_T_PS_A_PO_T_PS_T_PO_C)) (**32**) was obtained as the main product and successfully isolated with a 7% yield (Figure S18). This result indicated that this transformation reaction can be applied to PB/PO chimeric nucleic acid.

## Conclusions

We have developed an efficient method to synthesize a wide variety of *P*-modified nucleic acids, including doubly *P*-modified nucleic acid derivatives from PB derivatives in both liquid-phase synthesis and solid-phase synthesis. In the stereochemical analysis of the conversion reaction, the stereopure boranophosphodiesters, whose stereochemistry was unambiguously determined by the 2D NOESY experiment, were used for the transformation reaction to a phosphorothioate diester, and it was found that the reaction proceeded with retention of the configuration at the phosphorus atom. In addition, the conversion reaction was found to be applicable to the PB and PB/PO chimeric ODNs bearing all four nucleobases on a solid support. PS and PS/PO chimeric dodecamers were synthesized from PB and PB/PO chimeric dodecamers, respectively. To take these results into consideration, the conversion reaction would offer an efficient synthesis of a wider range of *P*-modified nucleic acid derivatives and *P*-modified chimeric nucleic acid derivatives including doubly *P*-modified ones, which have been difficult to synthesize by the existing method. Therefore, the synthesis of a wider range of *P*-modified nucleic acids is expected using this method, which leads to the discovery of new types of *P*-modified nucleic acids that have favorable physicochemical and biological properties.

## Experimental Section

### General Information

All reactions were conducted under an Ar atmosphere. Dry organic solvents were prepared by appropriate procedures. ^1^H NMR spectra were recorded at 400 MHz with tetramethylsilane (δ 0.00) as an internal standard in CDCl_3_ or at 500 MHz or 600 MHz with CH_3_CN (δ 2.06) as an internal standard in D_2_O. ^13^C NMR spectra were recorded at 101 MHz with CDCl_3_ (δ 77.0) as an internal standard in CDCl_3_. ^31^P NMR spectra were recorded at 162 MHz or 243 MHz with H_3_PO_4_ (δ 0.0) as an external standard in CDCl_3_, pyridine-d_5_, or D_2_O. NOESY spectra (mixing time of 400 ms) were recorded on Bruker Avance Neo 500 MHz spectrometer with a cryogenic probe (Bruker Biospin, Inc.). Analytical thin-layer chromatography was performed on commercial glass plates with a 0.25 mm thickness silica gel layer. Manual silica gel column chromatography was performed using spherical, neutral, 63–210 μm silica gel. Automated silica gel column chromatography was performed on silica gel (Yamazen UNIVERSAL Premium column (30 μm 60 Å)) using the automated flash chromatography system W-prep 2XY (Yamazen Corporation). Manual solid-phase synthesis was carried out using a glass filter (10 mm × 50 mm) with a stopper at the top and a stopcock at the bottom as a reaction vessel. Synthesized dimers by manual solid-phase synthesis were analyzed by reversed-phase HPLC. Synthesized oligomers (tetramer and dodecamers) were analyzed and purified by reverse-phase HPLC and identified by electrospray ionization (ESI) mass spectroscopy. Isolated yields of dodecamers were estimated by measuring UV–vis spectra using a molar absorption constant at 260 nm (ε = 1.135 × 10^5^ L/mol/cm).

### Reaction Analysis for the Formation of Acyl Phosphite by ^31^P NMR

Compound **4** (62.6 mg, 50 μmol) was dried by repeated coevaporation with dry pyridine and dissolved in pyridine-d_5_ (1 mL). PivCl (24 μL, 200 μmol) was added to the mixture at 0 °C while stirring. Then, the mixture (0.5 mL) was transferred into an NMR tube. The formation of the acyl phosphite (**5**) (δ 133.3 and 133.2 ppm) was confirmed by ^31^P NMR analysis after 15 and 45 min (Figure S1).

### Reaction Analysis for the Formation of Phosphorothioate Diester by ^31^P NMR

Compound **4** (63.0 mg, 50 μmol) was dried by repeated coevaporation with dry pyridine and dissolved in pyridine-d_5_ (1 mL). 3-Phenyl 1,2,4-dithiazoline-5-one (POS) (60.9 mg, 300 μmol) was added to the mixture and cooled to 0 °C. PivCl (24 μL, 200 μmol) was added to the mixture at 0 °C while stirring. Then, the mixture (0.50 mL) was transferred into an NMR tube. The formation of the mixed anhydride derivative and the phosphorothioate derivative (**7** and **8**) were confirmed by ^31^P NMR after 30 and 60 min. After that, a saturated NaHCO_3_ aqueous solution (0.1 mL) was added to the NMR tube, and the formation of the phosphorothioate diester (**8**) was confirmed by ^31^P NMR (Figure S2).

### Reaction Analysis for the Formation of the Mixed Anhydride Derivative and the Phosphorothioate Diester by ^31^P NMR

Compound **4** (33.7 mg, 25 μmol) and POS (14.8 mg, 77 μmol) were dried in an NMR tube in vacuo. Then, pyridine (500 μL) was added to the NMR tube and cooled to 0 °C. PivCl (12 μL, 100 μmol) was added to the mixture at 0 °C. The formation of the mixed anhydride derivative and the phosphorothioate derivative (**7** and **8**) were confirmed by ^31^P NMR after 60 min. After 140 min of adding POS, PivCl (30 μL, 250 μmol) was added to the NMR tube and the reaction mixture was analyzed by ^31^P NMR (Figure S3).

#### Triethylammonium 5′-*O*-Dimethoxytrityl-*N*^3^-benzoylthymidin-3′-yl 3′-*O*, *N*^3^-Dibenzoylthymidin-5′-yl Phosphorothioate Diester (**8**)

Compound **4** (64.0 mg, 51 μmol) was dried by repeated coevaporation with dry pyridine and dissolved in dry pyridine (1 mL). After adding POS (59.2 mg, 300 μmol) to the mixture, the reaction mixture was cooled to 0 °C. PivCl (24 μL, 200 μmol) was added to the mixture at 0 °C while stirring. The mixture was warmed to room temperature and stirred for 20 min. After that, the mixture was cooled to 0 °C, and a saturated NaHCO_3_ aqueous solution (5 mL) was added to the reaction mixture and stirred for 30 min. Then, the mixture was diluted with CHCl_3_ (5 mL) and the solution was washed with 1.0 M TEAB buffers (2 × 5 mL), and the combined aqueous layers were extracted with CHCl_3_ (2 × 5 mL). The organic layer was combined, dried over Na_2_SO_4_, filtered, and concentrated to dryness under reduced pressure. The residue was purified by silica gel column chromatography (5 g of neutral silica gel, 1 × 8.5 cm) using EtOAc–CH_2_Cl_2_–Et_3_N (80:20:1, v/v/v) as the eluent. The fractions containing **8** were collected and concentrated under reduced pressure to obtain **8** as a yellow foam (60.8 mg, 47.5 μmol, 93% yield *R*_f_ = 0.63 (CH_2_Cl_2_–MeOH = 4:1, v/v, neutral silica)). ^1^H NMR (400 MHz, CDCl_3_) δ 11.9–11.7 (brs, 1H), 8.10 (d, *J* = 0.9 Hz, 0.5H), 8.02–7.89 (m, 6.5H), 7.80 (s, 0.5H), 7.75 (s, 0.5H), 7.68–7.53 (m, 4H), 7.53–7.39 (m, 9H), 7.35–7.20 (m, 5H), 6.89–6.80 (m, 4H), 6.57–6.42 (m, 2H), 5.73 (d, *J* = 5.5 Hz, 0.5H), 5.50–5.32 (m, 1.5H), 4.46–4.22 (m, 3H), 4.20–4.01 (m, 1H), 3.78 (s, 1.5H), 3.78 (s, 1.5H), 3.77 (s, 1.5H), 3.76 (s, 1.5H), 3.59 (dd, *J* = 10.5, 2.3 Hz, 0.5H), 3.55–3.42 (m, 1.5H), 3.08 (q, *J* = 7.3 Hz, 6H), 2.84–2.74 (m, 0.5H), 2.66–2.59 (m, 0.5H), 2.59–2.32 (m, 3 H), 2.04 (s, 1.5H), 2.02 (s, 1.5H), 1.41 (s, 1.5H), 1.37 (s, 1.5H), 1.32 (t, *J* = 7.3 Hz, 9H); ^13^C{^1^H} NMR (101 MHz, CDCl_3_) δ 169.2, 169.1, 169.1, 169.0, 165.8, 165.7, 162.8, 158.7, 149.6, 149.6, 149.4, 149.2, 144.2, 144.2, 136.1, 135.7, 135.5, 135.4, 135.2, 135.2, 134.9, 133.5, 131.6, 131.6, 130.4, 130.4, 130.1, 129.6, 129.2, 129.1, 128.4, 128.2, 128.0, 127.1, 113.3, 111.8, 111.4, 111.2, 87.1, 85.9 (d, ^3^*J*_C–P_ = 4.8 Hz), 85.3 (d, ^3^*J*_C–P_ = 5.9 Hz), 85.0, 84.9, 84.8, 84.6, 83.9 (d, ^3^*J*_C–P_ = 7.7 Hz), 83.8 (d, ^3^*J*_C–P_ = 9.6 Hz), 83.7, 77.2, 76.3, 75.9, 67.9, 65.7 (d, ^2^*J*_C–P_ = 5.8 Hz), 65.4 (d, ^2^*J*_C–P_ = 6.7 Hz), 64.0, 63.9, 55.2, 45.7, 40.1 (d, ^3^*J*_C–P_ = 1.9 Hz), 39.3 (d, ^3^*J*_C–P_ = 3.9 Hz), 37.5, 37.3, 12.5, 11.6, 11.5, 8.6; ^31^P{^1^H} NMR (162 MHz, CDCl_3_) δ 57.7, 57.5. HRMS (ESI–TOF) *m*/*z* calcd for C_62_H_56_N_4_O_16_PS^–^ [M– Et_3_N–H]^−^, 1175.3155; found 1175.3078.

### Reaction Analysis for the Formation of Phosphitetriester by ^31^P NMR

Compound **4** (67.0 mg, 50 μmol) was dried by repeated coevaporation with dry pyridine and dissolved in dry pyridine (1 mL). Then, a part of the reaction mixture (500 μL) was transferred into an NMR tube. PivCl (12 μL, 100 μmol) was added to the mixture at 0 °C. After 5 min, the mixture was warmed to room temperature and allowed to stir for further 20 min. Then, the reaction mixture was cooled at 0 °C for 30 min, and EtOH (58 μL, 0.5 mmol) was added to the reaction mixture. The mixture was analyzed by ^31^P NMR after 60 min of the addition of EtOH (Figure S4).

#### 5′-*O*-Dimethoxytrityl-*N*^3^-benzoylthymidin-3′-yl 3′-*O*, *N*^3^-Dibenzoylthymidin-5′-yl Ethyl Phosphotriester (**10**)

Compound **4** (65.1 mg, 49 μmol) was dried by repeated coevaporation with dry pyridine and dissolved in dry pyridine (1 mL). PivCl (24 μL, 200 μmol) was added to the mixture at 0 °C while stirring. The mixture was warmed to room temperature after 5 min and allowed to stir for further 35 min. EtOH (58 μL, 1.0 mmol) was added to the mixture at 0 °C, and the mixture was warmed to room temperature after 5 min. The mixture was allowed to stir for further 40 min. A 0.26 M iodine in pyridine/H_2_O (98:2, v/v) (1 mL, 0.26 mmol) was added to the mixture and the mixture was allowed to stir further for 30 min. Then, the mixture was diluted with CHCl_3_ (10 mL) and washed with mixtures of a saturated NaHCO_3_ aqueous solution and a 10% Na_2_S_2_O_3_ aqueous solution (1:1, v/v) (3 × 5 mL). The combined aqueous layers were extracted with CHCl_3_ (3 × 10 mL). The organic layers were combined, dried over Na_2_SO_4_, filtered, and concentrated to dryness under reduced pressure. The residue was purified by silica gel column chromatography. Column chromatography was carried out on Yamazen UNIVERSAL Premium column (M size) using the automated flash chromatography system W-prep 2XY (Yamazen Corporation), which was performed with an isocratic elution of 47% Hexane in EtOAc over 3 min followed by an isocratic elution of 20% hexane in EtOAc over 10 min and an isocratic elution of 10% Hexane in EtOAc over 7 min to afford **10** as a colorless foam (47.7 mg, 40.1 μmol, 82% yield *R*_f_ = 0.53 (EtOAc–hexane = 9:1, v/v, neutral silica)).

^1^H NMR (400 MHz, CDCl_3_) δ 8.01–7.90 (m, 6H), 7.75–7.69 (m, 1H), 7.67–7.56 (m, 4H), 7.53–7.36 (m, 8H), 7.36–7.23 (m, 7H), 6.90–6.82 (m, 4H), 6.51–6.40 (m, 2H), 5.54–5.49 (m, 0.5H), 5.45–5.41 (m, 0.5H), 5.27–5.19 (m, 1H), 4.47–4.06 (m, 6H), 3.80 (s, 1.5H), 3.80 (s, 1.5H), 3.79 (s, 1.5H), 3.78 (s, 1.5H), 3.60 (dt, *J* = 11.0, 3.5 Hz, 1H), 3.44 (dt, *J* = 10.3, 2.3 Hz, 1H), 2.77–2.67 (m, 1H), 2.61–2.48 (m, 2H), 2.34–2.24 (m, 1H), 1.98 (d, *J* = 0.9 Hz, 1.5H), 1.96 (d, *J* = 0.9 Hz, 1.5H), 1.44–1.40 (m, 3 H), 1.39–1.25 (m, 3H); ^13^C{^1^H} NMR (101 MHz, CDCl_3_) δ 168.9, 168.9, 168.8, 168.7, 165.9, 165.9, 162.7, 162.7162.5, 158.8, 149.4, 149.4, 149.3, 143.9, 143.8, 135.0, 134.9, 134.8, 134.7, 133.7, 131.5, 131.5, 130.5, 130.4, 130.1, 129.7, 129.1, 128.8, 128.6, 128.1, 127.4, 113.3, 112.0, 111.8, 111.7, 87.4, 84.8, 84.7, 84.6, 84.4, 82.9 (d, ^3^*J*_C–P_ = 7.7 Hz), 82.8 (d, ^3^*J*_C–P_ = 8.7 Hz), 79.1, 79.1, 74.5, 74.3, 67.4 (d, ^2^*J*_C–P_ = 5.8 Hz), 67.2 (d, ^2^*J*_C–P_ = 5.8 Hz), 64.9 (d, ^2^*J*_C–P_ = 4.8 Hz), 64.8 (d, ^2^*J*_C–P_ = 4.8 Hz), 63.3, 63.2, 55.3, 39.3 (d, ^3^*J*_C–P_ = 3.9 Hz), 39.1 (d, ^3^*J*_C–P_ = 2.9 Hz), 37.3, 37.2, 16.2 (d, ^3^*J*_C–P_ = 1.9 Hz), 16.1 (d, ^3^*J*_C–P_ = 2.9 Hz), 12.5, 11.7; ^31^P{^1^H} NMR (162 MHz, CDCl_3_) δ – 0.7, −1.1; HRMS (ESI–TOF) *m*/*z* calcd for C_64_H_65_N_5_NaO_17_P^+^ [M + Na]^+^, 1211.3662; found 1211.3663.

### Reaction Analysis for the Formation of Phosphoramidite by ^31^P NMR

Compound **4** (67.0 mg, 50 μmol) was dried by repeated coevaporation with dry pyridine and dissolved in dry pyridine (1 mL). Then, a part of the reaction mixture (500 μL) was transferred into an NMR tube. PivCl (12 μL, 100 μmol) was added to the mixture at 0 °C. After 5 min, the mixture was warmed to room temperature, and allowed to stir for further 20 min. Then, the reaction mixture was cooled at 0 °C for 30 min, and propylamine (56 μL, 0.75 mmol) was added to the reaction mixture. The mixture was analyzed by ^31^P NMR after 50 min of the addition of propylamine (Figure S5).

#### 5′-*O*-Dimethoxytritylthymidin-3′-yl 3′-*O*-Benzoylthymidinyl Propyl Phosphoramidate Diester (**12**)

Compound **4** (61.0 mg, 50 μmol) was dried by repeated coevaporation with dry pyridine and dissolved in dry pyridine (1 mL). PivCl (24 μL, 200 μmol) was added to the mixture at 0 °C while stirring. The mixture was warmed to room temperature after 5 min and allowed to stir for further 60 min. Propylamine (110 μL, 1.5 mmol) was added to the mixture at 0 °C, and the mixture was warmed to room temperature after 5 min. The mixture was allowed to stir for further 30 min. A 5.5 M *t*-BuOOH decane solution (500 μL, 2.75 mmol) was added to the mixture at 0 °C, and the mixture was warmed to room temperature after 5 min. After stirring for 25 min, the reaction mixture was diluted with CHCl_3_ (5 mL) and washed with mixtures of a saturated NaHCO_3_ aqueous solution and a 10% Na_2_S_2_O_3_ aqueous solution (1:1, v/v) (3 × 5 mL). The combined aqueous layers were back-extracted with CHCl_3_ (3 × 5 mL). The organic layers were combined, dried over Na_2_SO_4_, filtered, and concentrated to dryness under reduced pressure. The residue was purified by silica gel column chromatography (15 g of amino silica gel, 2 × 8 cm) using CH_2_Cl_2_–MeOH–pyridine (100:0:1–100:4:1, v/v/v) as the eluent. The fractions containing **12** were collected and concentrated under reduced pressure to obtain **12** as a colorless foam (40.5 mg, 40.7 μmol, 85% yield *R*_f_ = 0.30 (EtOAc–MeOH = 9:1, v/v, amino silica)). ^1^H NMR (400 MHz, CDCl_3_) δ 8.78 (s, 0.5H), 8.71 (s, 1H), 8.69 (s, 0.5H), 8.06–7.99 (m, 2H), 7.64–7.53 (m, 2H), 7.50–7.40 (m, 2H), 7.39–7.33 (m, 2H), 7.32–7.21 (m, 8H), 6.86–6.79 (m, 4H), 6.49–6.38 (m, 1.5H), 6.35 (dd, *J* = 8.7, 5.5 Hz, 0.5H), 5.59–5.53 (m, 0.5H), 5.50–5.45 (m, 0.5H), 5.18–5.11 (m, 1H), 4.42–4.17 (m, 4H), 3.79 (s, 3H), 3.77 (s, 1.5H), 3.77 (s,1.5H), 3.53 (dt, *J* = 10.5, 2.7 Hz, 1H), 3.44 (dd, *J* = 10.8, 2.4 Hz, 0.5H), 3.36 (dd, *J* = 10.5, 2.7 Hz, 0.5H), 3.04–2.95 (m, 0.5H), 2.94–2.68 (m, 2H), 2.67–2.52 (m, 1.5H), 2.50–2.23 (m,2H), 1.93 (d, *J* = 0.9 Hz, 1.5H), 1.92 (d, *J* = 0.9 Hz, 1.5H), 1.41–1.36 (m, 3H), 0.95–0.82 (m, 5H); ^13^C{^1^H} NMR (101 MHz, CDCl_3_) δ 165.9, 163.7, 163.6, 158.8, 158.7, 150.5, 150.4, 150.4, 150.3, 144.1, 143.9, 135.3, 135.3, 135.1, 135.1, 135.0, 135.0, 134.9, 133.6, 130.1, 129.7, 128.9, 128.5, 128.1, 128.0, 127.2, 127.2, 113.3, 111.6, 87.2, 85.0, 85.0, 84.9, 84.4, 84.3, 82.9 (d, ^3^*J*_C–P_ = 7.7 Hz), 77.6, 74.7, 66.1 (d, ^2^*J*_C–P_ = 2.9 Hz), 65.9 (d, ^2^*J*_C–P_ = 4.8 Hz), 63.5, 63.3, 55.2, 43.2, 43.1, 39.1, 37.3, 37.1, 14.1, 12.5, 12.4, 11.6, 11.1, 11.0; ^31^P{^1^H} NMR (162 MHz, CDCl_3_) δ 9.6, 9.3. HRMS (ESI–TOF) m/z calcd for C_51_H_55_N_5_O_14_P^–^ [M–H]^−^, 993.3561; found 993.3548.

#### 5′-*O*-Dimethoxytrityl-*N*^3^-benzoylthymidin-3′-yl 3′-*O*, *N*^3^-Dibenzoylthymidin-5′-yl Ethyl Phosphorothioate Triester (**13**)

Compound **4** (67.8 mg, 51 μmol) was dried by repeated coevaporation with dry pyridine and dissolved in dry pyridine (1 mL). PivCl (24 μL, 200 μmol) was added to the mixture at 0 °C while stirring. The mixture was warmed to room temperature after 5 min and allowed to stir for further 40 min. EtOH (58 μL, 1.0 mmol) was added to the mixture at 0 °C, and the mixture was warmed to room temperature after 5 min. The mixture was allowed to stir for further 45 min. After adding POS (29.7 mg, 150 μmol) to the mixture at room temperature, the mixture was allowed to stir for 30 min. Then, the reaction mixture was diluted with CHCl_3_ (10 mL) and washed with mixtures of a saturated NaHCO_3_ aqueous solution and a 10% Na_2_S_2_O_3_ aqueous solution (1:1, v/v) (3 × 5 mL). The combined aqueous layers were extracted with CHCl_3_ (3 × 10 mL). The organic layers were combined, dried over Na_2_SO_4_, filtered, and concentrated to dryness under reduced pressure. The residue was purified by silica gel column chromatography. Column chromatography was carried out on Yamazen UNIVERSAL Premium column (M size) using the automated flash chromatography system W-prep 2XY (Yamazen Corporation), which was performed with an isocratic elution of 47% hexane in EtOAc over 10 min followed by an isocratic elution of 37% hexane in EtOAc over 7 min to afford **13** as a colorless foam (51.4 mg, 43 μmol, 84% yield *R*_f_ = 0.70 (EtOAc–hexane = 9:1, v/v, neutral silica)).

^1^H NMR (400 MHz, CDCl_3_) δ 8.00–7.89 (m, 6H), 7.74 (d, *J* = 0.9 Hz, 0.5H), 7.72 (d, *J* = 0.9 Hz, 0.5H), 7.68–7.55 (m, 4H), 7.53–7.39 (m, 8H), 7.38–7.27 (m, 7H), 6.90–6.84 (m, 4H), 6.50–6.42 (m, 2H), 5.56–5.50 (m, 0.5H), 5.47–5.39 (m, 1.5H), 4.51–4.04 (m, 6H), 3.80 (s, 3H), 3.79 (s, 1.5H), 3.79 (s, 1.5H), 3.54–3.46 (m, 2H), 2.75–2.45 (m, 3H), 2.34–2.23 (m, 1H), 2.00–1.98 (m, 3H), 1.48 (d, *J* = 0.9 Hz, 1.5H), 1.47 (d, *J* = 0.9 Hz, 1.5H), 1.37 (t, *J* = 7.1 Hz, 1.5H), 1.28 (t, *J* = 7.0 Hz, 1.5H); ^13^C{^1^H} NMR (101 MHz, CDCl_3_) δ 168.9, 168.9, 168.7, 168.7, 166.0, 165.9, 162.7, 162.7, 162.5, 158.8, 149.4, 149.4, 149.3, 149.3, 144.0, 135.0, 134.9, 134.7, 134.6, 133.7, 131.5, 131.5, 130.5, 130.4, 130.0, 129.7, 129.7, 129.1, 128.8, 128.5, 128.4, 128.1, 128.0, 127.3, 126.9, 113.4, 112.0, 111.8, 111.7, 111.6, 87.4, 84.9, 84.8, 84.8, 84.7, 84.6, 82.9 (d, ^3^*J*_C–P_ = 9.6 Hz), 79.7 (d, ^2^*J*_C–P_ = 3.9 Hz), 79.5 (d, ^2^*J*_C–P_ = 3.9 Hz), 74.9, 74.7, 67.5 (d, ^2^*J*_C–P_ = 5.8 Hz), 67.4 (d, ^2^*J*_C–P_ = 6.7 Hz), 65.3 (d, ^2^*J*_C–P_ = 5.8 Hz), 65.2 (d, ^2^*J*_C–P_ = 5.8 Hz), 63.3, 55.2, 39.3 (d, ^3^*J*_C–P_ = 4.8 Hz), 39.2 (d, ^3^*J*_C–P_ = 4.8 Hz), 37.4, 37.3, 15.9 (d, ^3^*J*_C–P_ = 6.7 Hz), 15.8 (d, ^3^*J*_C–P_ = 6.7 Hz), 12.6, 12.6, 11.8, 11.8; ^31^P{^1^H} NMR (162 MHz, CDCl_3_) δ 68.6, 68.4; HRMS (ESI–TOF) m/z calcd for C_64_H_61_N_4_NaO_16_PS^+^ [M + Na]^+^,1227.3433; found 1227.3437.

### Reaction Analysis for the Formation of Propyl Phosphorothioamidate by ^31^P NMR

Compound **4** (68.0 mg, 51 μmol) was dried by repeated coevaporation with dry pyridine and dissolved in dry pyridine (1 mL). PivCl (24 μL, 200 μmol) was added to the mixture at 0 °C while stirring. The mixture was warmed to room temperature after 2 min and allowed to stir for further 40 min. Propylamine (110 μL, 1.5 mmol) was added to the mixture at 0 °C, and the mixture was warmed to room temperature. The mixture was allowed to stir for 80 min. After adding POS (28.3 mg, 150 μmol) to the mixture at room temperature, the mixture was allowed to stir for further 80 min. Then, a part of the mixture (0.50 mL) was transferred into an NMR tube and analyzed by ^31^P NMR (Figure S6).

#### 5′-*O*-Dimethoxytritylthymidin-3′-yl 3′-*O*-Benzoylthymidinyl Propyl Phosphorothioamidate (**14**)

Compound **4** (65.2 mg, 49 μmol) was dried by repeated coevaporation with dry pyridine and dissolved in dry pyridine (1 mL). PivCl (24 μL, 200 μmol) was added to the mixture at 0 °C while stirring. The mixture was warmed to room temperature after 5 min and allowed to stir for further 40 min. Propylamine (110 μL, 1.5 mmol) was added to the mixture at 0 °C, and the mixture was warmed to room temperature after 10 min. The mixture was allowed to stir for 45 min. After adding POS (28.1 mg, 150 μmol) to the mixture at room temperature, the mixture was allowed to stir for further 60 min. Then, the reaction mixture was diluted with CHCl_3_ (10 mL) and washed with mixtures of a saturated NaHCO_3_ aqueous solution and a 10% Na_2_S_2_O_3_ aqueous solution (1:1, v/v) (3 × 10 mL). The combined aqueous layers were extracted with CHCl_3_ (3 × 10 mL). The organic layers were combined, dried over Na_2_SO_4_, filtered, and concentrated to dryness under reduced pressure. The residue was purified by silica gel column chromatography. Column chromatography was carried out on Yamazen UNIVERSAL Premium column (M size) using automated flash chromatography system W-prep 2XY (Yamazen Corporation), which was performed with an isocratic elution of EtOAc over 60 min followed by a linear gradient of 0%–30% MeOH in EtOAc for the first time, and with an isocratic elution of CH_2_Cl_2_ over 3 min followed by a linear gradient of 0–2% MeOH in CH_2_Cl_2_ for 10 min and 2–10% MeOH in CH_2_Cl_2_ for 10 min for the second time. Then, the fractions containing **14** were collected and concentrated under reduced pressure. The residue was dissolved in CHCl_3_ (3 mL), and hexane (50 mL) was added to induce precipitation. The precipitate was collected by filtration, washed with hexane (20 mL), and dried under reduced pressure to afford **14** as a colorless solid (33.9 mg, 34 μmol, 69% *R*_f_ = 0.30 (EtOAc–MeOH = 9:1, v/v, amino silica)).

^1^H NMR (400 MHz, CDCl_3_) δ 9.14 (brs, 2H), 8.07–8.00 (m, 2H), 7.63–7.57 (m, 2H), 7.46 (t, *J* = 7.8 Hz, 2H), 7.42–7.37 (m, 2H), 7.33–7.20 (m, 8H), 6.87–6.81 (m, 2H), 6.47–6.40 (m, 1.5H), 6.37 (dd, *J* = 8.9, 5.7 Hz, 0.5H), 5.58 (d, *J* = 6.4 Hz, 0.5H), 5.50 (d, *J* = 6.4 Hz, 0.5H), 5.40–5.27 (m, 1H), 4.42–4.24 (m, 3.5H), 4.23–4.15 (m, 0.5H), 3.79 (s, 3H), 3.78 (s, 3 H), 3.55–3.42 (m, 2H), 3.34–3.27 (m, 0.5H), 3.17–3.09 (m, 0.5H), 3.02–2.92 (m, 1H), 2.91–2.77 (m, 1H), 2.71–2.55 (m, 2H), 2.49–2.21 (m, 2H), 1.97 (d, *J* = 0.9 Hz, 1.5H), 1.92 (d, *J* = 0.9 Hz,1.5H), 1.58–1.37 (m, 5H), 0.93 (t, *J* = 7.3 Hz, 1.5H), 0.84 (t, *J* = 7.3 Hz, 1.5H); ^13^C{^1^H} NMR (101 MHz, CDCl_3_) δ 166.0, 165.9, 163.7, 163.7, 163.6, 158.7, 158.7, 150.5, 150.4, 150.3, 144.2, 144.1, 135.2, 135.1, 135.1, 135.1, 135.0, 134.9, 133.7, 130.1, 129.7, 129.0, 128.5, 128.0, 127.2, 127.2, 113.3, 111.6, 87.2, 85.1, 84.5, 84.5, 84.4, 83.0 (d, ^3^*J*_C–P_ = 9.6 Hz), 82.9 (d, ^3^*J*_C–P_ = 9.6 Hz), 78.5 (d, ^2^*J*_C–P_ = 2.9 Hz), 78.0 (d, ^2^*J*_C–P_ = 2.9 Hz), 75.0, 74.9, 66.1 (d, ^2^*J*_C–P_ = 3.9 Hz), 63.5, 55.2, 43.8 (d, ^2^*J*_C–P_ = 2.9 Hz), 43.6 (d, ^2^*J*_C–P_ = 2.9 Hz), 39.2 (d, ^3^*J*_C–P_ = 4.8 Hz), 37.5, 37.4, 24.7 (d, ^3^*J*_C–P_ = 5.8 Hz), 24.6 (d, ^3^*J*_C–P_ = 6.7 Hz), 12.6, 12.5, 11.7, 11.2, 11.1; ^31^P{^1^H} NMR (162 MHz, CDCl_3_) δ 74.5, 73.7; HRMS (ESI–TOF) m/z calcd for C_51_H_55_N_5_O_13_PS^–^ [M–H]^−^, 1008.3260; found 1008.3252.

### General Procedure for the Manual Solid-Phase Synthesis of Dithymidine Phosphotriester (**18**)

5′-*O*-DMTr-*N*^3^-benzoylthymidine on an HCP via a succinyl linker (0.5 μmol, 30.3 μmol/g for entries 1–7, 30.7 μmol/g for entry 8, 9) in a reaction vessel was treated with 3% DCA in dry CH_2_Cl_2_ (5 × 12 s, 1 mL each) and washed with dry CH_2_Cl_2_ (4 × 1 mL) and CH_3_CN (4 × 1 mL). Thereafter, it was dried in vacuo for 10 min. To the reaction vessel, the 3′-*H*-boranophosphonate monomer unit (**16t**^15^ (16.7 mg, 20 μmol, 40 equiv, 0.1 M) and MNTP^23^ (22.3 mg, 50 μmol, 100 equiv) as a condensing reagent were added and 2,6-lutidine (0.6 M, 120 μmol) in dry CH_3_CN (200 μL) was added under Ar, and the reaction vessel was stirred slowly with hands for 3 min. The HCP was washed with dry CH_3_CN (4 × 1 mL) and dry CH_2_Cl_2_ (4 × 1 mL) and dried in vacuo for 10 min. The *H*-boranophosphonate internucleotide linkage was oxidized by treatment with a solution (500 μL) of 0.1 M Et_3_N in CCl_4_–2,6-lutidine–H_2_O (5:12.5:1, v/v/v) for 90 min. The HCP was washed with dry CH_3_CN (4 × 1 mL) and dry CH_2_Cl_2_ (4 × 1 mL), and dried in vacuo for 5 min. The internucleotidic linkages were treated with a solution of 2.0 M PivCl (49.2 μL, 400 μmol) in pyridine (200 μL) for 5 (entry 1), 15 (entries 2), or 60 min (entries 3–9). Then, the HCP was washed with solution A (entries 1–3), solution B (entry 4), solution C (entry 5), or solution D (entries 6–9) (3 × 1 mL). The HCP was treated with solution A (entries 1–3), solution B (entry 4), solution C (entry 5), solution D (entries 6–9) for 40 min (solution A: 6.5 M EtOH in pyridine; B: 6.5 M EtOH in 2,6-lutidine; C: 6.5 M EtOH and 1.0 M DIPEA in CH_3_CN; D: 6.5 M EtOH and 1.0 M DMAN in CH_3_CN). After that, the HCP was washed with CH_3_CN (3 × 1 mL) and dried in vacuo for 5 min (entries 7–9) (In entries 1–6, the HCP was oxidized without wash treatment). The HCP was treated with a 5.5 M *t*-BuOOH decane solution (200 μL) for 30 min. The HCP was washed with dry CH_3_CN (8 × 1 mL) and CH_2_Cl_2_ (8 × 1 mL), and dried in vacuo for 5 min. 3% DCA in dry CH_2_Cl_2_ (4 × 15 s, 1 mL) was added to the reaction vessel, and the HCP was washed with dry CH_2_Cl_2_ (4 × 1 mL) and CH_3_CN (4 × 1 mL). The HCP was treated with concentrated aqueous NH_3_–EtOH (3:1, v/v, 5 mL) at room temperature for 3 h (entries 1–7), room temperature for 1 h (entry 8), or at 50 °C in a thermostatic chamber for 12 h (entry 9), filtrated, and washed with EtOH (3 × 1 mL). The filtrate and washings were combined and concentrated under reduced pressure. The residue was analyzed by RP-HPLC. RP-HPLC was performed with a linear gradient of 0%–30% CH_3_CN for 60 min in a 0.1 M triethylammonium acetate (TEAA) buffer (pH 7.0) at 30 °C with a flow rate of 0.5 mL/min using a C18 column (5 μm, 100 Å, 3.9 mm × 150 mm).

### Procedure for the Manual Solid-Phase Synthesis of Dithymidine Boranophosphodiester (**22**)

HCP-loaded 5′-*O*-DMTr-*N*^3^-benzoylthymidine, via a succinyl linker (0.5 μmol, 30.3 μmol/g), in a reaction vessel was treated with 3% DCA in dry CH_2_Cl_2_ (5 × 12 s, 1 mL each) and washed with dry CH_2_Cl_2_ (4 × 1 mL) and CH_3_CN (4 × 1 mL). Thereafter, it was dried in vacuo for 10 min. To the reaction vessel, the 3′-*H*-boranophosphonate monomer unit (**16t** (16.7 mg, 20 μmol, 40 equiv, 0.1 M)) and MNTP (22.3 mg, 50 μmol, 100 equiv) as a condensing reagent were added, then 2,6-lutidine (0.6 M, 120 μmol) in dry CH_3_CN (200 μL) was added under Ar, and the reaction vessel was stirred slowly with hands for 3 min. The HCP was washed with dry CH_3_CN (4 × 1 mL) and CH_2_Cl_2_ (4 × 1 mL), and the detritylation reaction was carried out by treatment with 3% DCA in dry CH_2_Cl_2_–Et_3_SiH (1:1, v/v) (5 × 15 s, 1 mL each). Therefore, the HCP was washed with dry CH_2_Cl_2_ (4 × 1 mL) and dry CH_3_CN (4 × 1 mL), and dried in vacuo for 10 min. The *H*-boranophosphonate internucleotide linkage was oxidized by treatment with a solution (200 μL) of 0.1 M TEA in CCl_4_–2,6-lutidine–H_2_O (5:12.5:1, v/v/v) for 90 min. The HCP was washed with dry CH_3_CN (4 × 1 mL) and CH_2_Cl_2_ (4 × 1 mL), and dried in vacuo. The HCP was treated with concentrated aqueous NH_3_–EtOH (3:1, v/v, 5 mL) at room temperature for 3 h, filtrated, and washed with EtOH (3 × 1 mL). The filtrate and washings were combined and concentrated under reduced pressure. The residue was analyzed by RP-HPLC. RP-HPLC was performed with a linear gradient of 0%–30% CH_3_CN for 60 min in a 0.1 M triethylammonium acetate (TEAA) buffer (pH 7.0) at 30 °C with a flow rate of 0.5 mL/min using a C18 column (5 μm, 100 Å, 3.9 mm × 150 mm).

### Procedure for the Manual Solid-Phase Synthesis of Dithymidine Phosphorothioate Diester (**23**)

HCP-loaded 5′-*O*-DMTr-*N*^3^-benzoylthymidine, via a succinyl linker (0.5 μmol, 32.3 μmol/g), in a reaction vessel was treated with 3% DCA in dry CH_2_Cl_2_ (5 × 12 s, 1 mL each) and washed with dry CH_2_Cl_2_ (4 × 1 mL) and CH_3_CN (4 × 1 mL). Thereafter, it was dried in vacuo for 10 min. To the reaction vessel, the 3′-*H*-boranophosphonate monomer unit (**16t** (16.7 mg, 20 μmol, 40 equiv, 0.1 M)) and MNTP (22.3 mg, 50 μmol, 100 equiv) as a condensing reagent were added, then 2,6-lutidine (0.6 M, 120 μmol) in dry CH_3_CN (200 μL) was added under Ar, and the reaction vessel was stirred slowly with hands for 3 min. The HCP was washed with dry CH_3_CN (4 × 1 mL) and CH_2_Cl_2_ (4 × 1 mL) and dried in vacuo for 10 min. The *H*-boranophosphonate internucleotide linkage was oxidized by treatment with a solution (500 μL) of 0.1 M Et_3_N in CCl_4_–2,6-lutidine–H_2_O (5:12.5:1, v/v/v) for 90 min. The HCP was washed with dry CH_3_CN (4 × 1 mL) and CH_2_Cl_2_ (4 × 1 mL), and dried in vacuo for 5 min. The internucleotidic linkages were sulfurized with a solution of 1.5 M POS (58.6 mg, 300 μmol) and 1.0 M PivCl (24.6 μL, 200 μmol) in pyridine (200 μL) for 90 min and the HCP was washed with dry CH_2_Cl_2_ (4 × 1 mL) and dry CH_3_CN (4 × 1 mL). 3% DCA in dry CH_2_Cl_2_ (4 × 15 s, 1 mL) was added to the reaction vessel, and the HCP was washed with dry CH_2_Cl_2_ (4 × 1 mL) and CH_3_CN (4 × 1 mL). The HCP was treated with concentrated aqueous NH_3_–EtOH (3:1, v/v, 5 mL) at room temperature for 3 h, filtrated, and washed with EtOH (3 × 1 mL). The filtrate and washings were combined and concentrated under reduced pressure. The residue was analyzed by RP-HPLC. RP-HPLC was performed with a linear gradient of 0%–30% CH_3_CN for 60 min in a 0.1 M triethylammonium acetate (TEAA) buffer (pH 7.0) at 30 °C with a flow rate of 0.5 mL/min using a C18 column (5 μm, 100 Å, 3.9 mm × 150 mm).

### Synthesis of the Highly Cross-Linked Polystyrene with a Succinyl Sarcosyl Linker (**24t**)

*N*-(((9H-Fluoren-9-yl)methoxy)carbonyl)-*N*-methylglycine (Fmoc-Sar-OH, 50 mg, 0.15 mmol) was added to the round bottom flask and dried by repeated co-evaporation with toluene. HCP (33 μg/mol, 1.68 g, 0.055 mmol) and dry CH_2_Cl_2_ (8.5 mL) were added to the vessel successively. PyNTP (150 mg, 0.30 mmol) and DIPEA (45 μL, 0.26 mmol) was added to the suspension under the Ar atmosphere. The reaction mixture was rotated at room temperature for 3 h. After the HCP was washed with dry pyridine and dry CH_2_Cl_2_, 10 mL of capping solution (pyridine–Ac_2_O, 9:1, v/v) was added to the HCP and the mixture was rotated at room temperature for 5 h. After the HCP was washed with dry pyridine and dry CH_2_Cl_2_, 10 mL of a solution of CH_2_Cl_2_–piperidine (8:2, v/v) was added to the HCP and the mixture was rotated at room temperature for 1 h. After the HCP was washed with dry pyridine and dry CH_2_Cl_2_, HCP was dried in vacuo for 1 d to give HCP bearing *N*-methylglycine. 5′-O-DMTr-3′-succynyl-thymidine (90 mg, 0.12 mmol) was added to the round bottom flask and dried by repeated co-evaporation with toluene. HCP bearing *N*-methylglycine (0.99 g, 0.030 mmol) was added to the vessel, and then dry CH_2_Cl_2_ (5.0 mL) was added to the vessel. PyNMP (90 mg, 0.18 mmol) and DIPEA (50 μL, 0.29 mmol) were added to the mixture under Ar atmosphere. The reaction mixture was rotated at room temperature for 3 h. After the HCP was washed with dry pyridine and dry CH_2_Cl_2_, 10 mL of a capping solution (pyridine–Ac_2_O, 9:1, v/v) was added to the HCP and the mixture was rotated at room temperature for 1 h. After the HCP was washed with dry pyridine and dry CH_2_Cl_2_, it was dried in vacuo for 1 day to give the HCP with a succinyl sarcosyl linker (**24t**). The amount of loaded thymidine to the solid support was 29.6 μmol/g from the calculation of released 4,4′-demethoxytrityl cation by a solution of 0.1 M TsOH/CH_3_CN.

### General Procedure for the Manual Solid-Phase Stereocontrolled Synthesis of (*R*p) and (*S*p)-Dithymidine Boranophosphodiester ((*R*p) and (*S*p)-**22**)

5′-*O*-DMTr-thymidine on an HCP via a succinyl sarcosyl linker (0.5 μmol, 29.6 μmol/g) was used for the experiment. It should be noted that the succinyl sarcosyl linker was adopted to prevent the linker from releasing from a solid support under the treatment with 10% DBU in dry CH_2_Cl_2_.^31^ The HCP in a reaction vessel was treated with 3% DCA in dry CH_2_Cl_2_ (5 × 12 s, 1 mL each) and washed with dry CH_2_Cl_2_ (3 × 1 mL) and CH_3_CN (3 × 1 mL). Thereafter, it was dried in vacuo for 5 min. To the reaction vessel, the (*R*p) or (*S*p)-T oxazaphospholidine monomer unit ((*R*p) or (*S*p)-**25**, 16.9 mg, 22.5 μmol, 45 equiv) was added and then it was dried in vacuo for 5 min. Thereafter, 1.0 M CMPT/CH_3_CN–*i*PrCN (7:3, v/v) (150 μL, 150 μmol, 300 equiv) as a nonnucleophilic acidic activator was added to the reaction vessel followed by stirring slowly for 5 min under Ar. The HCP was washed with dry CH_3_CN (3 × 1 mL) and dry CH_2_Cl_2_ (3 × 1 mL) and dried in vacuo for 5 min. The obtained phosphite triester was boronated with a solution (1 mL) of 0.05 M BH_3_·THF in THF for 2 min. The HCP was washed with dry THF (3 × 1 mL), dry EtOH (3 × 1 mL), and dry CH_2_Cl_2_ (3 × 1 mL). 3% DCA in dry CH_2_Cl_2_–Et_3_SiH (1:1, v/v) (5 × 12 s, 1 mL) was added to the reaction vessel, and the HCP was washed with dry CH_2_Cl_2_ (3 × 1 mL) and CH_3_CN (3 × 1 mL). The HCP was treated with a solution (200 μL) of 0.25 M BSTFA in dry CH_3_CN for 5 min. Then, the HCP was washed with dry CH_3_CN (3 × 1 mL) followed by the treatment with 20% EtOH in dry CH_3_CN (1 mL) for 1 min. Thereafter, the HCP was washed with dry CH_3_CN (3 × 1 mL) and dried in vacuo for 5 min. After that, the HCP was treated with 10% DBU in dry CH_3_CN (500 μL) for 1 h to remove the chiral auxiliary. The HCP was treated with dry CH_3_CN (6 × 1 mL) and then it was dried in vacuo for 5 min. The HCP was treated with concentrated aqueous NH_3_–EtOH (3:1, v/v, 5 mL) at room temperature for 17 h, filtrated and washed with EtOH (3 × 1 mL). The filtrate and washings were combined and concentrated under reduced pressure. The residue was analyzed and purified by RP-HPLC. RP-HPLC was performed with a linear gradient of 5%–25% CH_3_CN for 20 min in a 0.1 M triethylammonium acetate (TEAA) buffer (pH 7.0) at 30 °C with a flow rate of 0.5 mL/min using a C18 column (5 μm, 100 Å, 3.9 mm × 150 mm). The obtained products were analyzed by ^1^H NMR, and the spectra were corresponded to the data in the literature.^28^

### Procedure for the Manual Solid-Phase Synthesis of (*S*p) and (*R*p)-Dithymidine Phosphorothioate Diester ((*S*p) and (*R*p)-**23**)

5′-*O*-DMTr-thymidine on an HCP via a succinyl sarcosyl linker (0.5 μmol, 29.58 μmol/g) in a reaction vessel was treated with 3% DCA in dry CH_2_Cl_2_ (5 × 12 s, 1 mL each) and washed with dry CH_2_Cl_2_ (3 × 1 mL) and CH_3_CN (3 × 1 mL). Thereafter, it was dried in vacuo for 5 min. To the reaction vessel, the (*R*p) or (*S*p)-T oxazaphospholidine monomer unit ((*R*p) or (*S*p)-**25**, 16.9 mg, 22.5 μmol, 45 equiv, 0.15 M) was added, and then it was dried in vacuo for 5 min. Thereafter, 1.0 M CMPT/CH_3_CN–*i*PrCN (7:3, v/v) (150 μL) as a nonnucleophilic acidic activator was added to the reaction vessel followed by stirring slowly with hands for 5 min under Ar. The HCP was washed with dry CH_3_CN (3 × 1 mL) and dry CH_2_Cl_2_ (3 × 1 mL) and dried in vacuo for 5 min. The obtained phosphite triester was boronated with a solution (1 mL) of 0.05 M BH_3_·THF in THF for 2 min. The HCP was washed with dry THF (3 × 1 mL), dry EtOH (3 × 1 mL), and dry CH_2_Cl_2_ (3 × 1 mL). After that, the HCP was treated with 10% DBU in dry CH_3_CN (500 μL) for 1 h to remove the chiral auxiliary. The HCP was treated with dry CH_3_CN (6 × 1 mL), and then it was dried in vacuo for 5 min. The internucleotidic linkage was sulfurized with a solution of 1.5 M POS (58.6 mg, 300 μmol) and 1.0 M PivCl (24.6 μL, 200 μmol) in dry pyridine (200 μL) for 90 min, and the HCP was washed with dry CH_2_Cl_2_ (4 × 1 mL) and dry CH_3_CN (4 × 1 mL). Three percent DCA in dry CH_2_Cl_2_ (4 × 15 s, 1 mL) was added to the reaction vessel, and the HCP was washed with dry CH_2_Cl_2_ (4 × 1 mL) and CH_3_CN (4 × 1 mL). The HCP was treated with concentrated aqueous NH_3_–EtOH (3:1, v/v, 5 mL) at room temperature for 3 h, filtered, and washed with EtOH (3 × 1 mL). The filtrate and washings were combined and concentrated under reduced pressure. The residue was analyzed and purified by RP-HPLC. RP-HPLC was performed with a linear gradient of 0%–30% CH_3_CN for 60 min in a 0.1 M triethylammonium acetate (TEAA) buffer (pH 7.0) (for analysis) or a linear gradient of 5%–25% CH_3_CN for 20 min in a 0.1 M ammonium acetate (AA) buffer (pH 7.0) (for purification) at 30 °C with a flow rate of 0.5 mL/min using a C18 column (5 μm, 100 Å, 3.9 mm × 150 mm). The obtained products were analyzed by ^1^H NMR and ^31^P NMR and the spectra corresponded to the data in the literature.^26^

### Synthesis of PS Tetramer (**29**), PS Dodecamer (**30**), and PS/PO Dodecamer (**32**)

HCP-loaded 5′-*O*-DMTr-*N*^3^-benzoyl thymidine (0.5 μmol, 30.7 μmol/g) or 5′-*O*-DMTr-*N*^4^-isobutyryl deoxycytidine (0.5 μmol, 22.0 μmol/g) via a succinyl linker, was treated with 3% DCA in dry CH_2_Cl_2_ (5 × 12 s, 1 mL each) and washed with dry CH_2_Cl_2_ (4 × 1 mL) and CH_3_CN (4 × 1 mL), and then dried in vacuo for 10 min. Chain elongations were conducted by repeating steps (a) and (b) 3 times (for the synthesis of **29**) or 11 times (for the synthesis of **30** and **32**).

(a) Condensation step: to introduce a PB or PO (for the synthesis of **32**) linkage, the *H*-boranophosphonate (**16a**, **c**, **g**, or **t**, 20 μmol) or *H*-phosphonate monomer (**31a**, **c**, **g**, or **t**, 20 μmol) and MNTP (22.3 mg, 50 μmol) were added to the reaction vessel and a condensation reaction was performed with 0.6 M 2,6-lutidine in CH_3_CN (200 μL) for 3 min.
(b) Detritylation step: the HCP was treated with 3% DCA in dry CH_2_Cl_2_–Et_3_SiH (1:1, v/v) (4 × 15 s, 1 mL each) and washed with dry CH_2_Cl_2_ (4 × 1 mL) and CH_3_CN (4 × 1 mL), and then dried in vacuo for 10 min.

After the designed length was achieved, the resultant internucleotide linkages were oxidized with a solution (500 μL) of 0.1 M Et_3_N in CCl_4_–2,6-lutidine–H_2_O (5:12.5:1, v/v/v) for 90 min. The HCP was washed with dry CH_2_Cl_2_ (4 × 1 mL) and CH_3_CN (4 × 1 mL) and then dried in vacuo for 10 min. The internucleotidic linkages were sulfurized with a solution of 1.5 M POS (58.6 mg, 300 μmol) and 1.0 M PivCl (24.6 μL, 200 μmol) in pyridine (200 μL) for 90 min, and the HCP was washed with dry CH_2_Cl_2_ (4 × 1 mL) and CH_3_CN (4 × 1 mL). 3% DCA in dry CH_2_Cl_2_ – Et_3_SiH (1:1, v/v) (4 × 15 s, 1 mL) was added to the reaction vessel, and the HCP was washed with dry CH_2_Cl_2_ (4 × 1 mL) and CH_3_CN (4 × 1 mL). The HCP was treated with concentrated aqueous NH_3_ – EtOH (3:1, v/v, 5 mL) at 50 °C in a thermostatic chamber for at least 12 h, filtered, and washed with EtOH. The filtrate and washings were combined and concentrated under reduced pressure. The residue was analyzed by RP-HPLC and then purified by RP-HPLC.

RP-HPLC was performed with a linear gradient of 0%–60% CH_3_CN for 60 min in a 0.1 M triethylammonium acetate (TEAA) buffer (pH 7.0) at 30 °C with a flow rate of 0.5 mL/min using a C18 column (5 μm, 100 Å, 3.9 mm × 150 mm) for **29**.

RP-HPLC was performed with a linear gradient of 5%–40% MeOH in a buffer containing 400 mM hexafluoro isopropanol (HFIP) and 8 mM triethylamine at 60 °C for 20 min with a flow rate of 0.5 mL/min using a C18 column (5 μm, 100 Å, 3.9 mm × 150 mm) for **30** and **32**.

Isolated yields: 14% (d(G_PS_C_PS_A_PS_T_PS_T_PS_G_PS_G_PS_T_PS_A_PS_T_PS_T_PS_C) (**30**)); 7% (d(G_PO_C_PS_A_PO_T_PS_T_PO_G_PS_G_PO_T_PS_A_PO_T_PS_T_PO_C) (**32**)); HRMS (ESI/Q-TOF): *m/z* calcd for d(C_PS_A_PS_G_PS_T) (**29**) [M–2H]^2–^ 609.5818; found 609.5836. calcd for d(G_PS_C_PS_A_PS_T_PS_T_PS_G_PS_G_PS_T_PS_A_PS_T_PS_T_PS_C) (**30**) [M–6H]^6–^, 636.7247; found 636.7242. calcd for d(G_PO_C_PS_A_PO_T_PS_T_PO_G_PS_G_PO_T_PS_A_PO_T_PS_T_PO_C) (**32**) [M–6H]^6–^, 620.7476; found 620.7471.

## Data Availability

The data underlying this study are available in the published article and its online Supporting Information.

## Supporting Information Available

Experimental procedures, copies of ^1^H, ^13^C {^1^H}, ^31^P NMR spectra for new compounds, and HPLC profiles (PDF)
