# Supplementary material for: Synthesis of P-Modified DNA from Boranophosphate DNA as a Precursor via Acyl Phosphite Intermediates
Source: J Org Chem. 2023 Jul 18;88(15):10617–31. doi: 10.1021/acs.joc.3c00659 (PMC10407935; doi:10.1021/acs.joc.3c00659)

Synthesis of *P*-modified DNA from boranophosphate DNA as a precursor via acyl phosphite intermediates

Yuhei Takahashi[†], Kiyoshi Kakuta[†], Yukichi Namioka[†], Ayumi Igarashi[‡], Taiichi Sakamoto[¶],

Rintaro Iwata Hara[†, §], Kazuki Sato[†]*, Takeshi Wada[†]*

[†]Department of Medicinal and Life Sciences, Faculty of Pharmaceutical Sciences, Tokyo University of Science, 2641 Yamazaki, Noda, Chiba 278–8510, Japan

[‡] Department of Medical Genome Sciences, Graduate School of Frontier Sciences, The University of Tokyo, Kashiwa, Chiba 277-8562, Japan

[¶] Department of Life Science, Chiba Institute of Technology, Graduate School of Advanced Engineering, Chiba 275-0016, Japan

[§] Department of Neurology and Neurological Science, Graduate School of Medicinal and Dental Sciences, Tokyo Medical and Dental University, Tokyo 113-8519

Supporting Information

Table of Contents

Experimental procedure of the synthesis of boranophosphodiester	S3
^{31}P NMR analysis of the formation of acylphosphite (4 equiv, 15 min, 45 min) (Figure S1)	S5
^{31}P NMR analysis of the formation of phosphorothioate (30 min, 60 min, after quench) (Figure S2)	S6
^{31}P NMR analysis of the formation of the mixed anhydride derivative and the phosphorothioate diester	S7
^{31}P NMR analysis of the formation of the phosphite triester (9) (Figure S4)	S8
^{31}P NMR analysis of the formation of the phosphoramidite (11) (Figure S5)	S9
^{31}P NMR spectrum of a crude mixture of the phosphorothioamidate (14) (Figure S6)	S10
RP-HPLC profiles of the dithymidylate phosphotriester (Figure S7)	S11
^{1}H NMR spectrum of (*R*p)-22 (500 MHz, D_2O) (Figure S8)	S13
^{1}H NMR spectrum of (*S*p)-22 (500 MHz, D_2O) (Figure S9)	S13
NOESY spectrum (*R*p)-PB diester ((*R*p)-22) (Figure S10)	S14
NOESY spectrum (*S*p)-PB diester ((*S*p)-22) (Figure S11)	S14
^{31}P NMR spectrum of (*S*p)-23 (243 MHz, D_2O) (Figure S12)	S15
^{1}H NMR spectrum of (*S*p)-23 (600 MHz, D_2O) (Figure S13)	S15
^{31}P NMR spectrum of (*R*p)-23 (243 MHz, D_2O) (Figure S14)	S16
^{1}H NMR spectrum of (*R*p)-23 (600 MHz, D_2O) (Figure S15)	S16
RP-HPLC profile of phosphorothioate tetramer (Figure S16)	S17
RP-HPLC profiles of PS dodecamer (Figure S17)	S18
RP-HPLC profiles of PS/PO dodecamer (Figure S18)	S19
NMR spectra of new compounds	S20

General Information

All reactions were conducted under an Ar atmosphere. Dry organic solvents were prepared by appropriate procedures. ^{1}H NMR spectra were recorded at 400 MHz with tetramethylsilane (δ 0.00) as an internal standard in $CDCl_3$ or at 500 MHz or 600 MHz with CH_3CN (δ 2.06) as an internal standard in D_2O. ^{13}C NMR spectra were recorded at 101 MHz with $CDCl_3$ (δ 77.0) as an internal standard in $CDCl_3$. ^{31}P NMR spectra were recorded at 162 MHz or 243 MHz with H_3PO_4 (δ 0.0) as an external standard in $CDCl_3$, pyridine-d_5 or D_2O. NOESY spectra (mixing time of 400 ms) were recorded on Bruker Avance Neo 500 MHz spectrometer with cryogenic probe (Bruker Biospin, Inc.). Analytical thin-layer chromatography was performed on commercial glass plates with a 0.25 mm thickness silica gel layer. Manual silica gel column chromatography was performed using spherical, neutral, 63−210 µm silica gel. Automated silica gel column chromatography was performed on silica gel (Yamazen UNIVERSAL Premium column (30 µm 60 Å)) using automated flash chromatography system W-prep 2XY (Yamazen Corporation). Manual solid-phase synthesis was carried out using a glass filter (10 mm × 50 mm) with a stopper at the top and a stopcock at the bottom as a reaction vessel. Synthesized dimers by manual solid-phase synthesis were analyzed by reversed-phase HPLC. Synthesized oligomers (tetramer and dodecamers) were analyzed and purified by reverse-phase HPLC and identified by electrospray ionization (ESI) mass spectroscopy. Isolated yields of dodecamers were estimated by measuring UV−vis spectra using a molar absorption constant at 260 nm (ε = 1.135 × 10^5 L/mol/cm).

5′-*O*-Dimethoxytrityl-*N*^{3}-Benzoylthymidin-3′-yl 3′-*O*, *N*^{3}-Dibenzoylthymidin-5′-yl Cyanoethyl Boranophosphotriester (S3)

Compound **S1** (0.77 g, 1.71 mmol) and **S2**[1] (1.69 g, 2.03 mmol) were dissolved in dry CH_3CN (17 mL). 2,6-Lutidine (1.96 mL, 17 mmol) and 3-nitro-1,2,4-triazol-1-yl-tris(pyrrolidin-1-yl) phosphonium hexafluorophosphate PyNTP (2.45 g, 4.90 mmol) were added successively to the solution. After being stirred at rt for 80 min, the mixture was diluted with $CHCl_3$ (50 mL). The solution was washed with saturated NaHCO3 aqueous solutions (3 × 50 mL), and the combined aqueous layers was extracted with $CHCl_3$ (2 × 50 mL). The organic layers were combined, dried over Na_2SO_4, filtered, and concentrated to dryness under reduced pressure. The residue was purified by silica gel column chromatography (80 g of neutral silica gel, 3.5 × 13 cm) using toluene–EtOAc (2:1, v/v) as the eluent. The fractions containing **S3** were collected and concentrated under reduced pressure to obtain **S3** as a colorless foam (1.70 g, 1.40 mmol, 82% yield R_f=0.80 (CH_2Cl_2:MeOH=95:5, v/v, neutral silica)). ^{1}H NMR (400 MHz, $CDCl_3$) 8.01–7.88 (m, 6H), 7.75–7.54 (m, 4H), 7.53–7.36 (m, 8H), 7.36–7.22 (m, 8H), 6.91–6.82 (m, 4H), 6.49–6.36 (m, 2H), 5.53–5.48 (m, 0.5H), 5.41–5.36 (m, 0.5H), 5.31–5.23 (m, 1H), 4.50–4.40 (m, 1H), 4.37–4.10 (m, 5H), 3.79 (s, 1.5H), 3.79 (s, 1.5H), 3.78 (s, 1.5H), 3.78 (s, 1.5H), 3.63–3.43 (m, 2H), 2.76–2.49 (m, 5H), 2.43–2.27 (m, 1H), 1.99–1.96 (m, 3H), 1.49 (s, 3H), 1.0–0.1 (brs, 3H); ^{13}C{^{1}H} NMR (101 MHz, $CDCl_3$) 168.9, 168.9, 168.7, 166.0, 165.9, 162.7, 162.6, 162.5, 158.8, 149.3, 143.9, 143.9, 135.1, 135.0, 134.9, 134.9, 134.8, 134.6, 133.8, 131.5, 131.4, 130.5, 130.1, 129.7, 129.1, 128.7, 128.7, 128.6, 128.1, 128.0, 127.3, 127.3, 116.2, 116.2, 113.4, 112.1, 112.0, 111.8, 111.7, 87.4, 85.0, 84.9, 84.7, 84.6, 82.7, 82.7, 82.6, 79.3, 79.2, 74.2, 74.1, 66.0, 63.2, 63.2, 61.9, 61.8, 55.3, 39.6, 39.4, 36.9, 36.9, 19.8, 19.7, 19.7, 19.6, 12.6, 11.8; ^{31}P{^{1}H} NMR (162 MHz, $CDCl_3$) 120.5–116.9. HRMS (ESI–TOF) *m/z* calcd for $C_{65}H_{67}BN_6O_{16}P^+$ $[M+NH_4]^+$, 1228.4476; found 1228.4476.

Triethylammonium 5′-*O*-Dimethoxytrityl-*N*^{3}-Benzoylthymidin-3′-yl 3′-*O*, *N*^{3}-Dibenzoylthymidin-5′-yl Boranophosphate (4)

Compound **S3** (1.62 g, 1.34 mmol) was dissolved in dry CH_2Cl_2 (20 mL) and Et_3N (2.75 mL, 19.8 mmol) was added to the solution. After being stirred at rt for 4 h, the mixture was concentrated to dryness under reduced pressure and then the mixture

was dried by repeated coevaporation with toluene. The residue was purified by silica gel column chromatography (70 g of neutral silica gel, 3.0 × 13 cm) using CH_2Cl_2–EtOAc–MeOH–Et_3N (65:35:0:1–65:35:2:1, v/v/v/v) as the eluent. The fractions containing **4** were collected and concentrated under reduced pressure to obtain **4** as a colorless foam (1.40 g, 1.11 mmol, 85% yield R_f=0.63 (CH_2Cl_2:MeOH=4:1, v/v, neutral silica)). ^{1}H NMR (400 MHz, $CDCl_3$) 12.3–12.1 (brs, 1H), 8.04–7.88 (m, 7H), 7.84 (s, 0.5H), 7.78 (s, 0.5H), 7.67–7.53 (m, 3H), 7.52–7.38 (m, 7H), 7.36–7.19 (m, 8H), 6.91–6.78 (m, 4H), 6.58 (dd, J = 8.4 Hz, 5.7 Hz, 0.5H), 6.54–6.43 (m, 1.5H), 5.76 (d, J = 5.0 Hz, 0.5H), 5.33 (d, J = 4.6 Hz, 0.5H), 5.25–5.15 (m, 1H), 4.55–4.45 (m, 0.5H), 4.40–4.29 (m, 1.5H), 4.21 (s, 0.5H), 4.17–4.04 (m, 1H), 3.99–3.90 (m, 0.5H), 3.80–3.73 (m, 6H), 3.61–3.42 (m, 2H), 3.02 (q, J = 7.2 Hz, 6H), 2.78–2.66 (m, 0.5H), 2.63–2.29 (m, 3.5H), 2.05 (s, 1.5H), 2.00 (s, 1.5H), 1.43 (s, 1.5H), 1.37 (s, 1.5H), 1.28 (t, J = 7.1 Hz, 9H), 1.0–0.1 (m, 3H); ^{13}C{^{1}H} NMR (101 MHz, $CDCl_3$) 169.2, 169.1, 169.0, 168.9, 165.8, 165.7, 162.9, 162.8, 158.7, 158.7, 158.6, 158.6, 149.6, 149.5, 149.3, 149.2, 144.2, 144.1, 135.9, 135.6, 135.6, 135.3, 135.1, 135.1, 134.9, 134.8, 133.4, 131.6, 131.6, 130.4, 130.3, 130.1, 130.1, 130.0, 129.5, 129.2, 129.1, 129.0, 128.4, 128.1, 128.0, 127.9, 127.1, 127.1, 113.3, 111.9, 111.8, 111.3, 111.1, 87.1, 87.1, 86.1 (d, $^3J_{C-P}$ = 3.9 Hz), 85.7 (d, $^3J_{C-P}$ = 3.9 Hz), 85.1, 84.9, 84.6, 84.5, 84.1 (d, $^3J_{C-P}$ = 4.8 Hz), 84.0 (d, $^3J_{C-P}$ = 6.7 Hz), 83.9, 76.7, 76.0, 75.4, 75.1, 64.0, 63.9, 62.0 (d, $^2J_{C-P}$ = 6.7 Hz), 61.5 (d, $^2J_{C-P}$ = 7.7 Hz), 55.2, 45.6, 40.5, 39.7, 37.4, 37.1, 12.5, 11.6, 11.5, 8.5; ^{31}P{^{1}H} NMR (162 MHz, $CDCl_3$) 98.3–91.4. HRMS (ESI–TOF) *m*/*z* calcd for $C_{62}H_{59}BN_4O_{16}P^-$ [M–Et_3N–H]$^-$, 1157.3762; found 1157.3707.

Scheme S1 Synthesis of dithymidine boranophosphate diester.

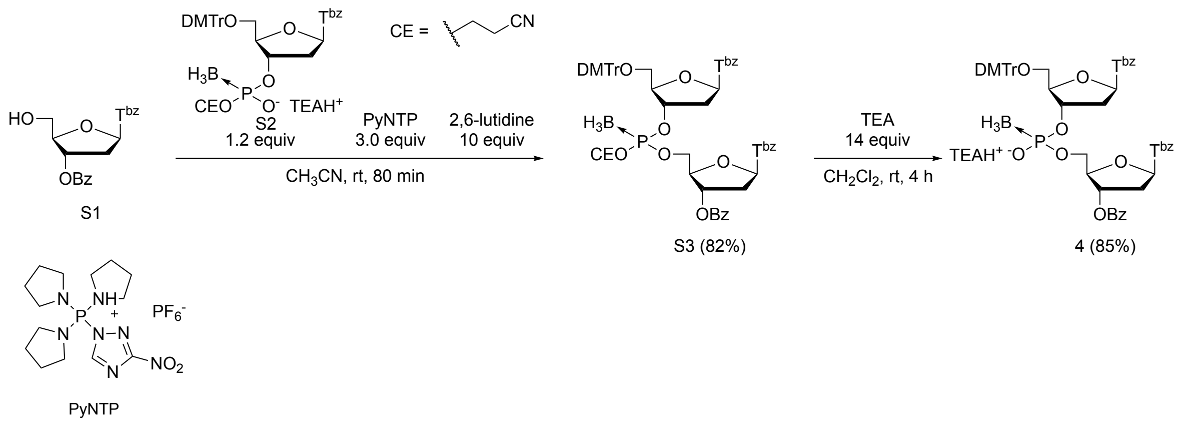

Reference for Supporting information

(1) Shimizu, M.; Saigo, K.; Wada, T. Solid-Phase Synthesis of Oligodeoxyribonucleoside Boranophosphates by the Boranophosphotriester Method. *J. Org. Chem.* **2006**, *71* (11), 4262–4269. https://doi.org/10.1021/jo0603779.

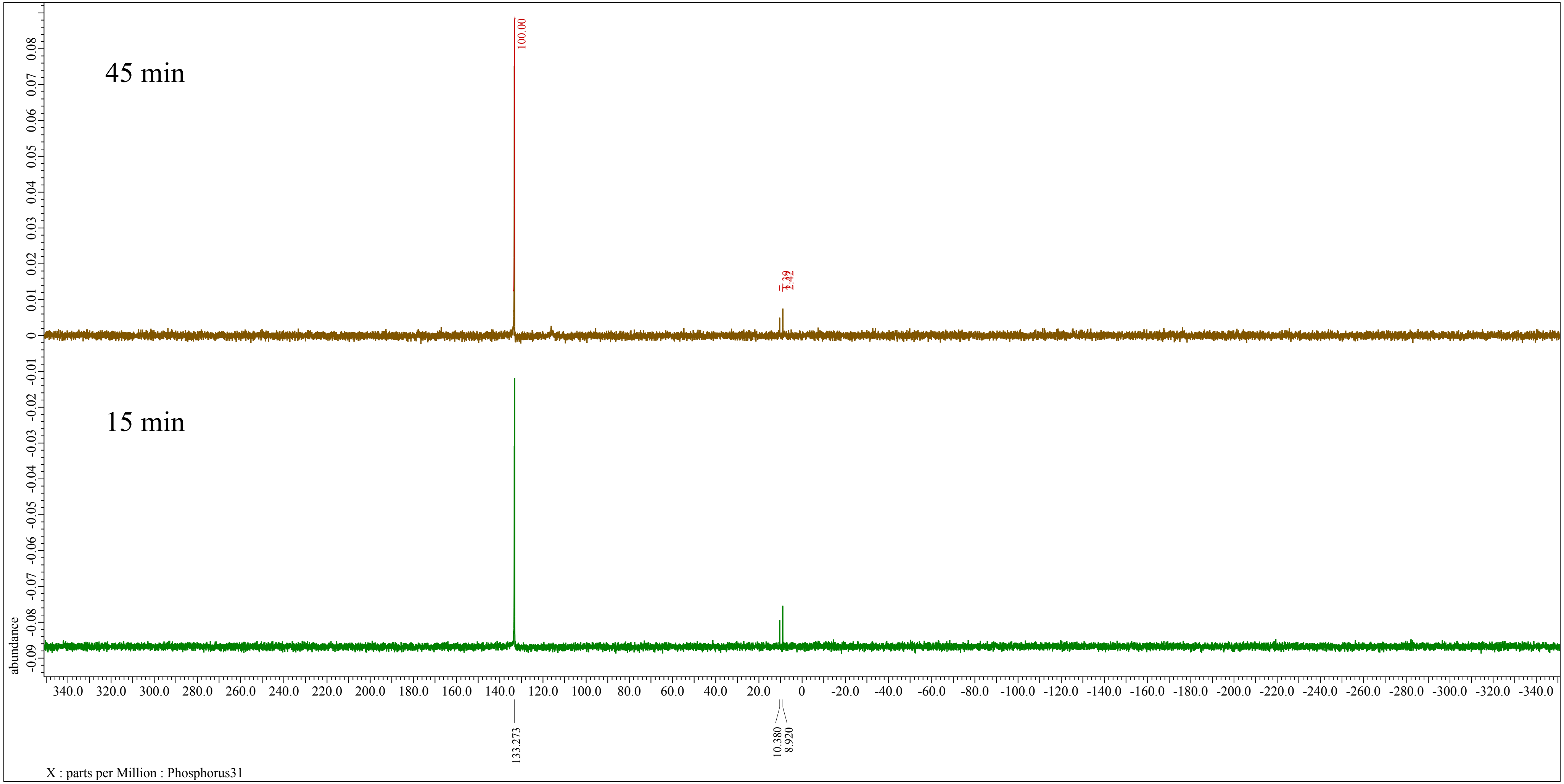

Figure S1 ^{31}P NMR analysis of the formation of acylphosphite (4 equiv, 15 min, 45 min)

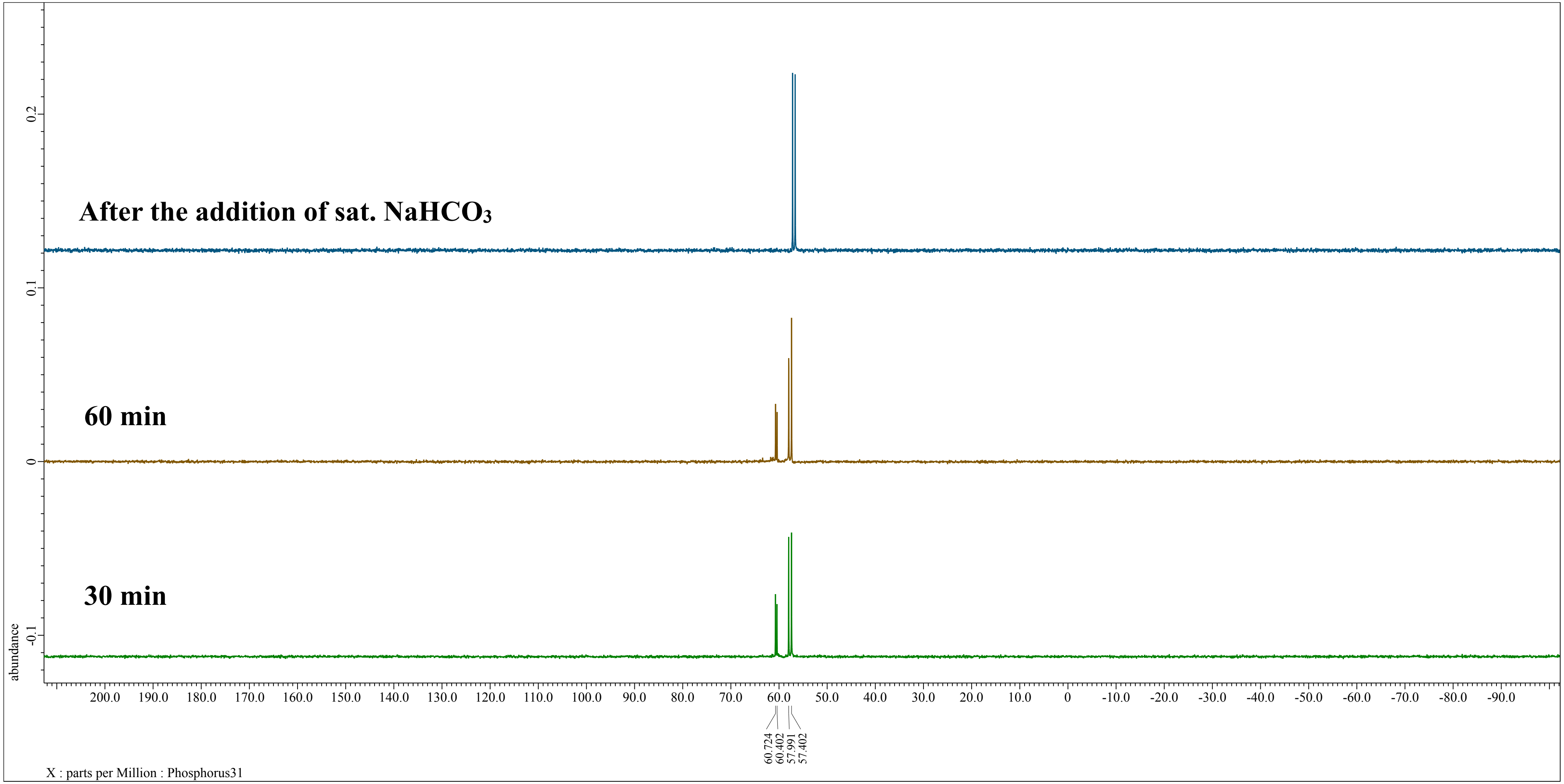

Figure S2 ^{31}P NMR analysis of the formation of phosphorothioate (30 min, 60 min, after quench)

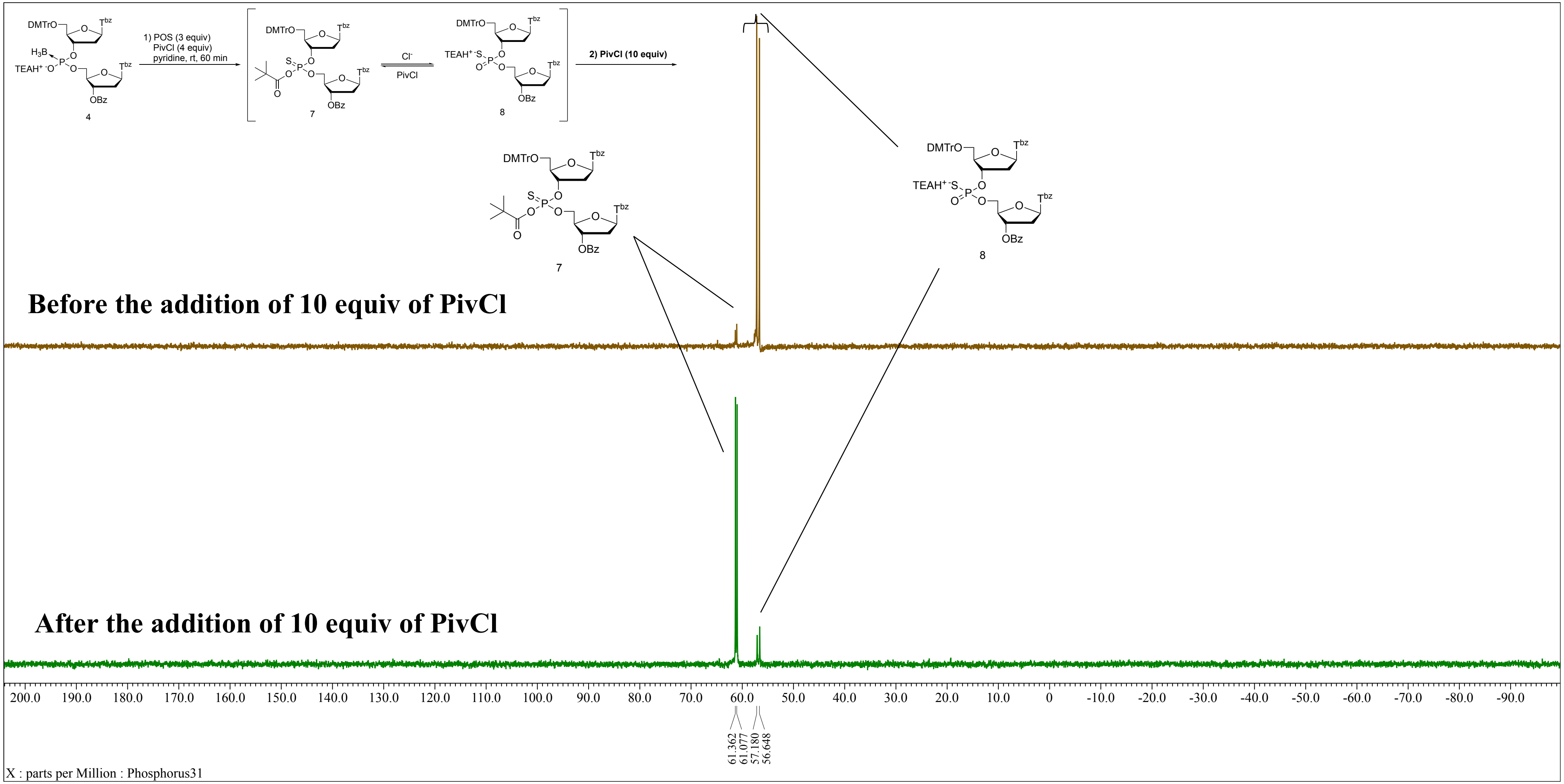

Figure S3 ^{31}P NMR analysis of the formation of the mixed anhydride derivative and the phosphorothioate diester

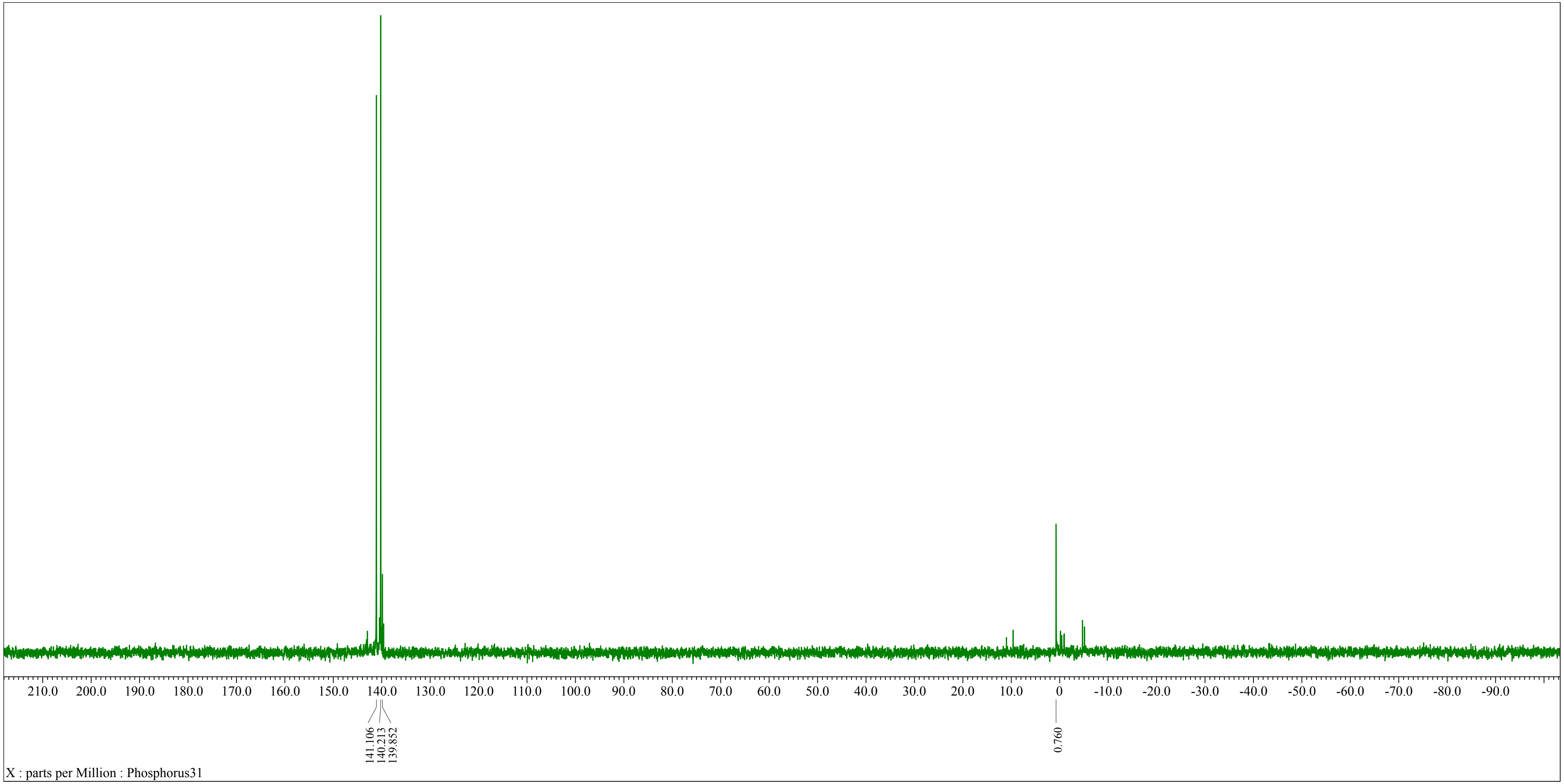

Figure S4 ^{31}P NMR analysis of the formation of the phosphite triester (9)

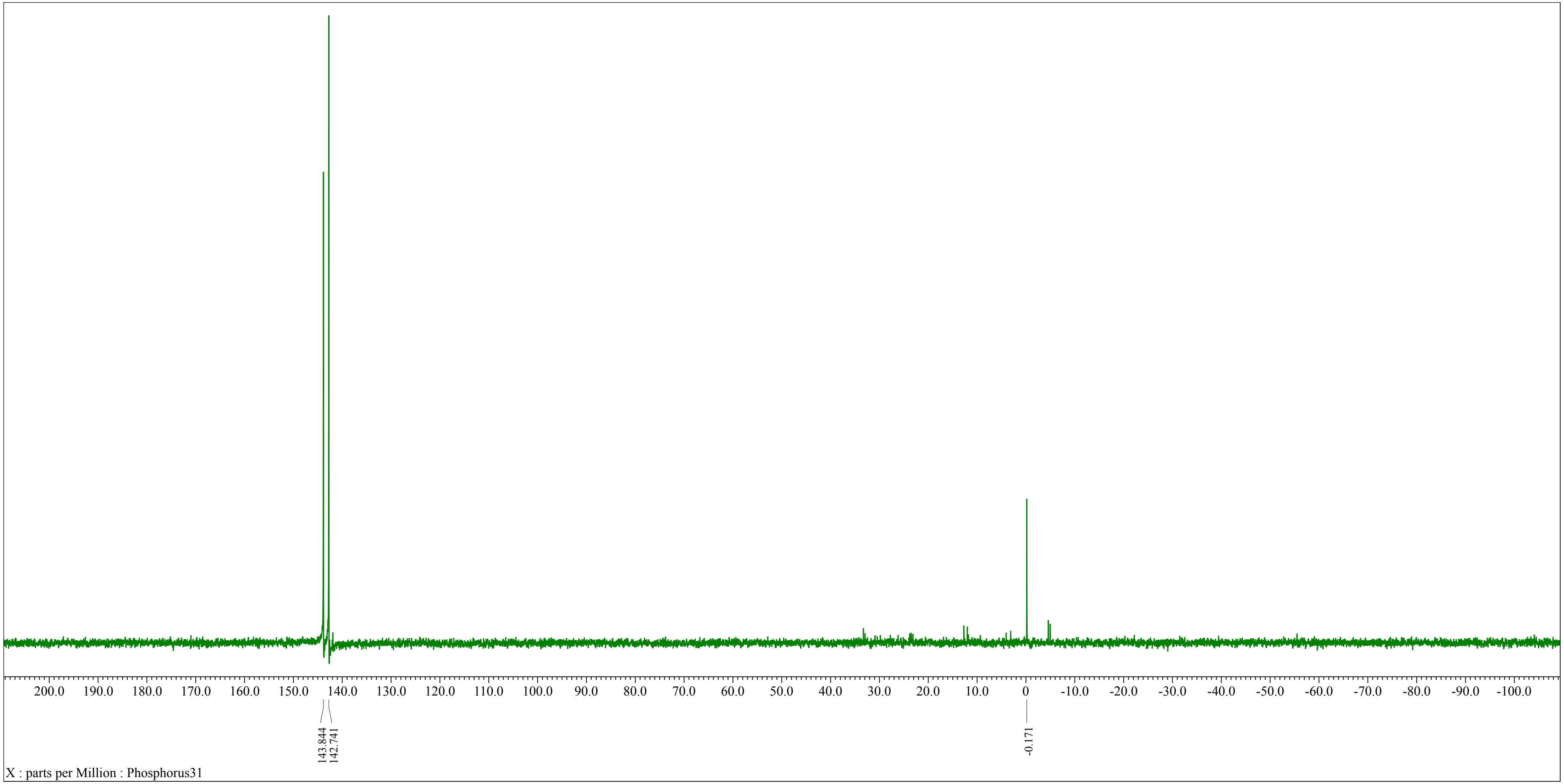

Figure S5 ^{31}P NMR analysis of the formation of the phosphoramidite (11)

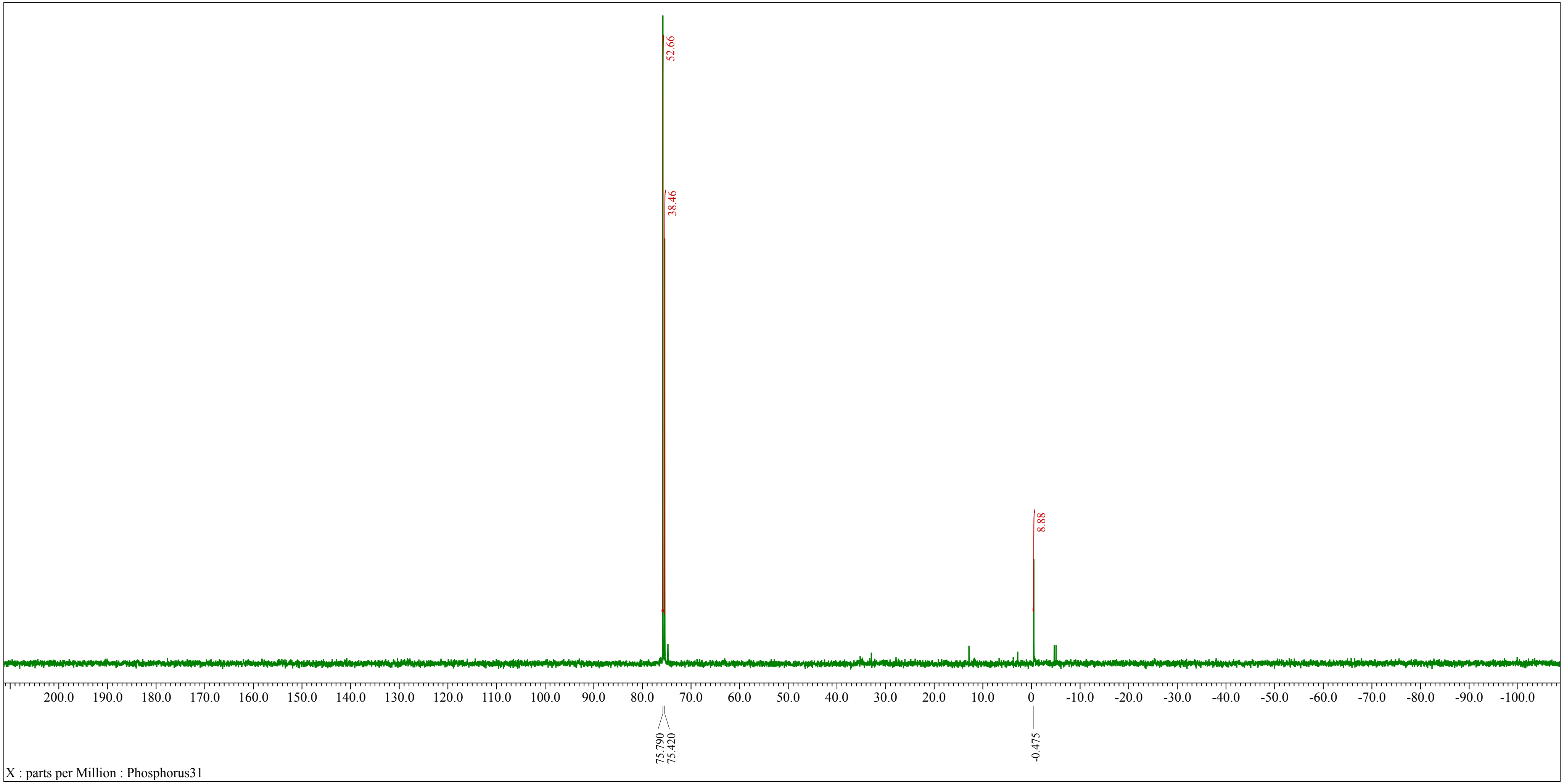

Figure S6 ^{31}P NMR spectrum of a crude mixture of the phosphorothioamidate (14)

RP-HPLC profiles of the dithymidylate phosphotriester (Table 1)

entry 1

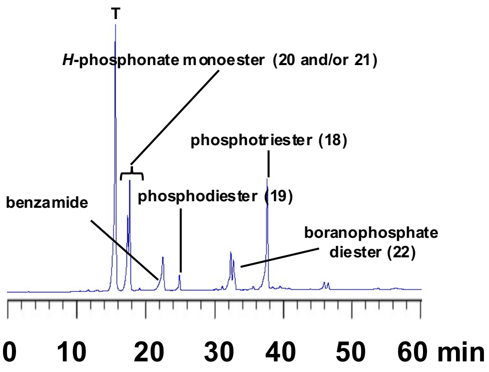

entry 2

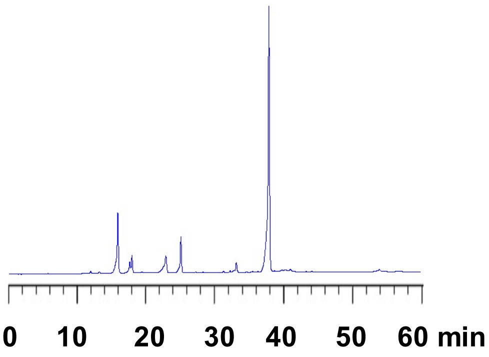

entry 3

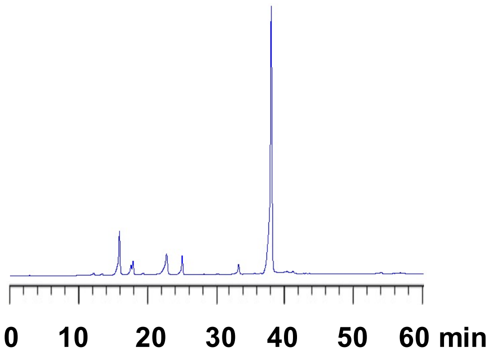

entry 4

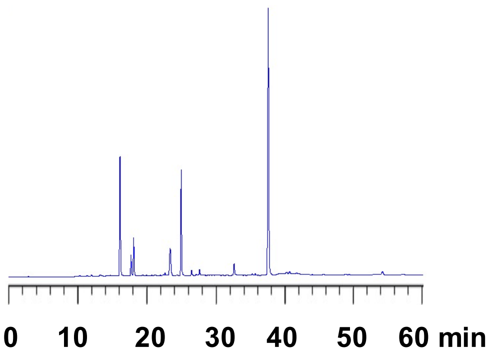

entry 5

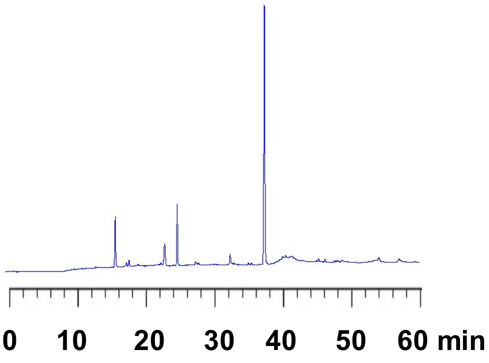

entry 6

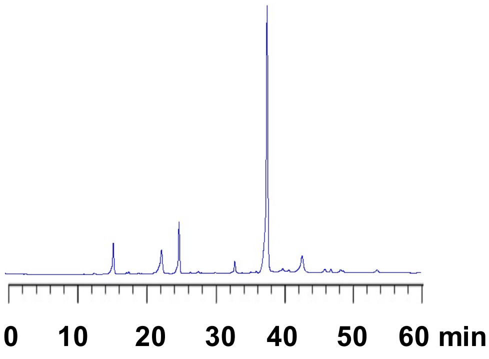

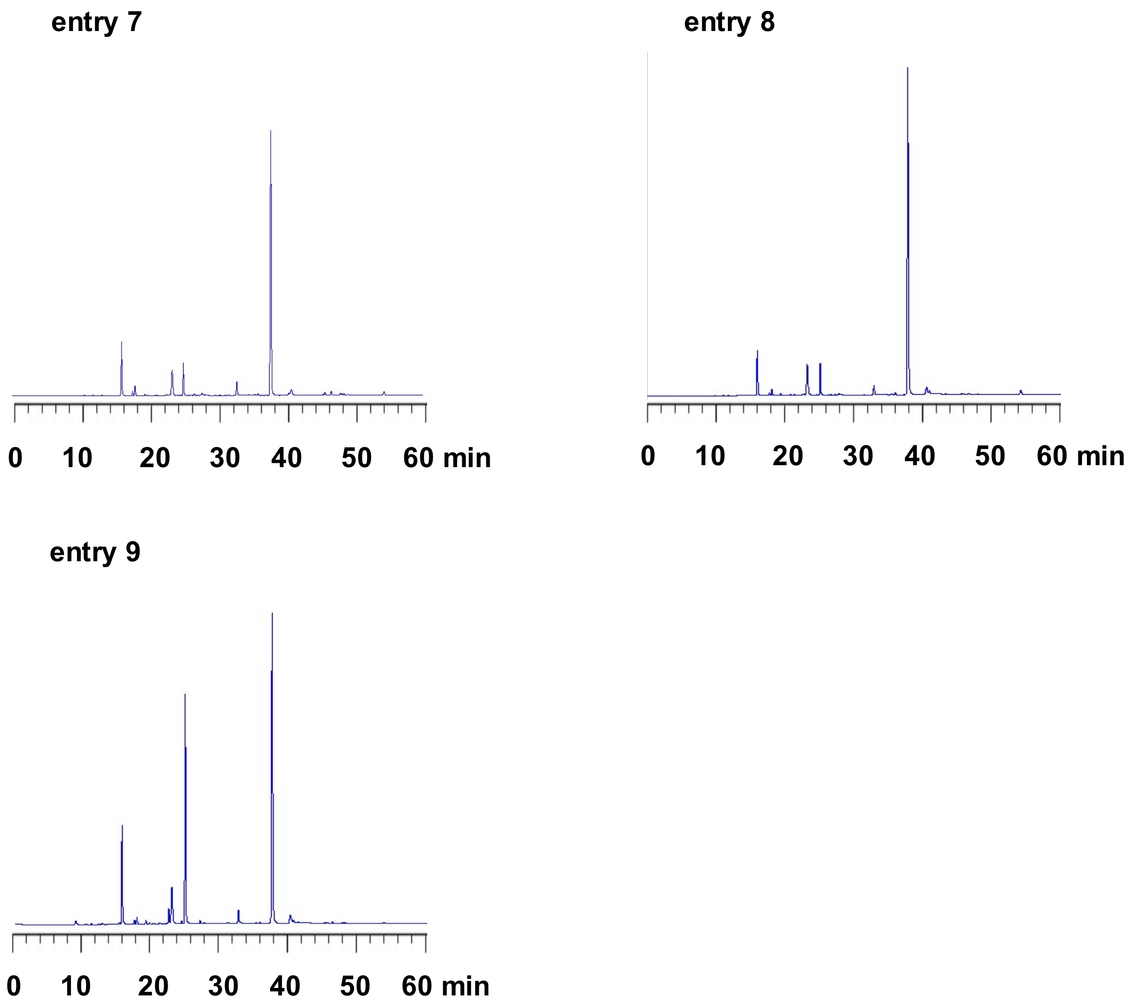

Figure S7 RP-HPLC was performed with a linear gradient of 0%–30% acetonitrile for 60 min in 0.1 M triethylammonium acetate buffer (pH 7.0) at 30 °C at a flow rate of 0.5 mL/min using a C18 column.

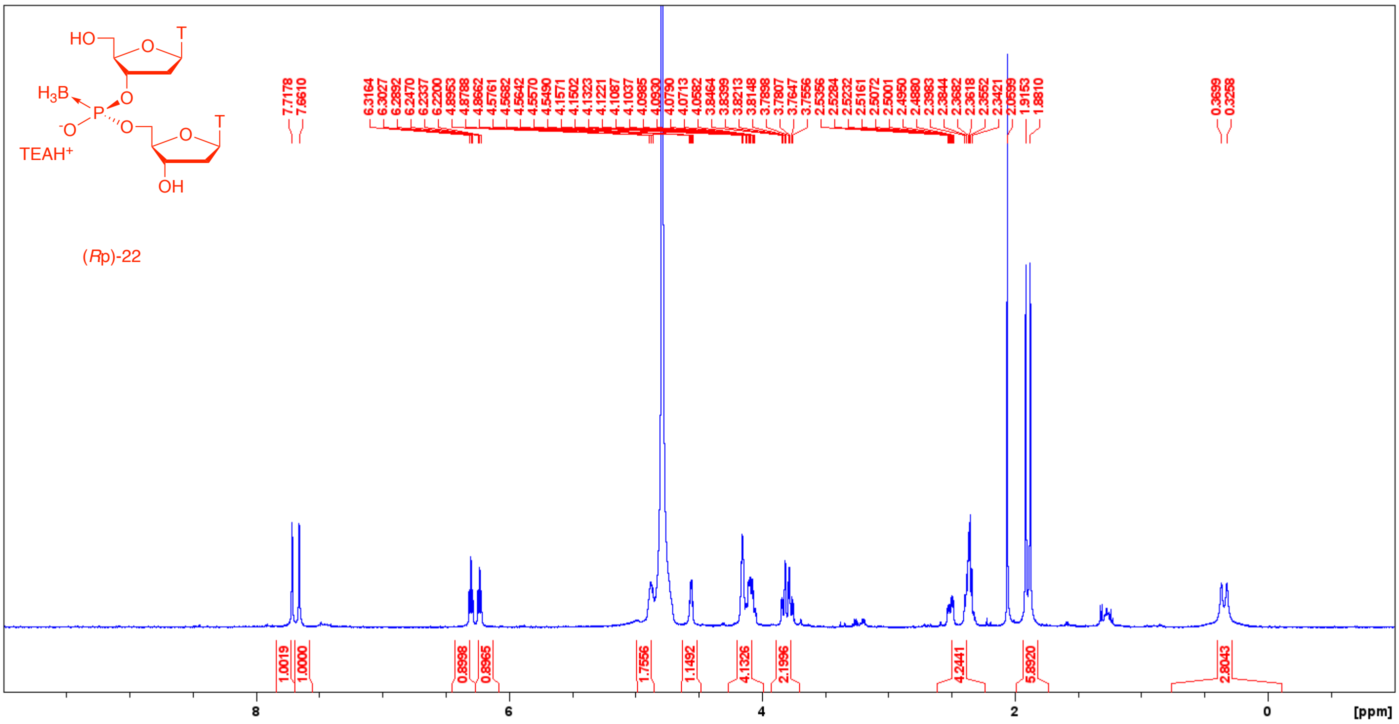

Figure S8 ^{1}H NMR spectrum of (*R*p)-22 (500 MHz, D_2O)

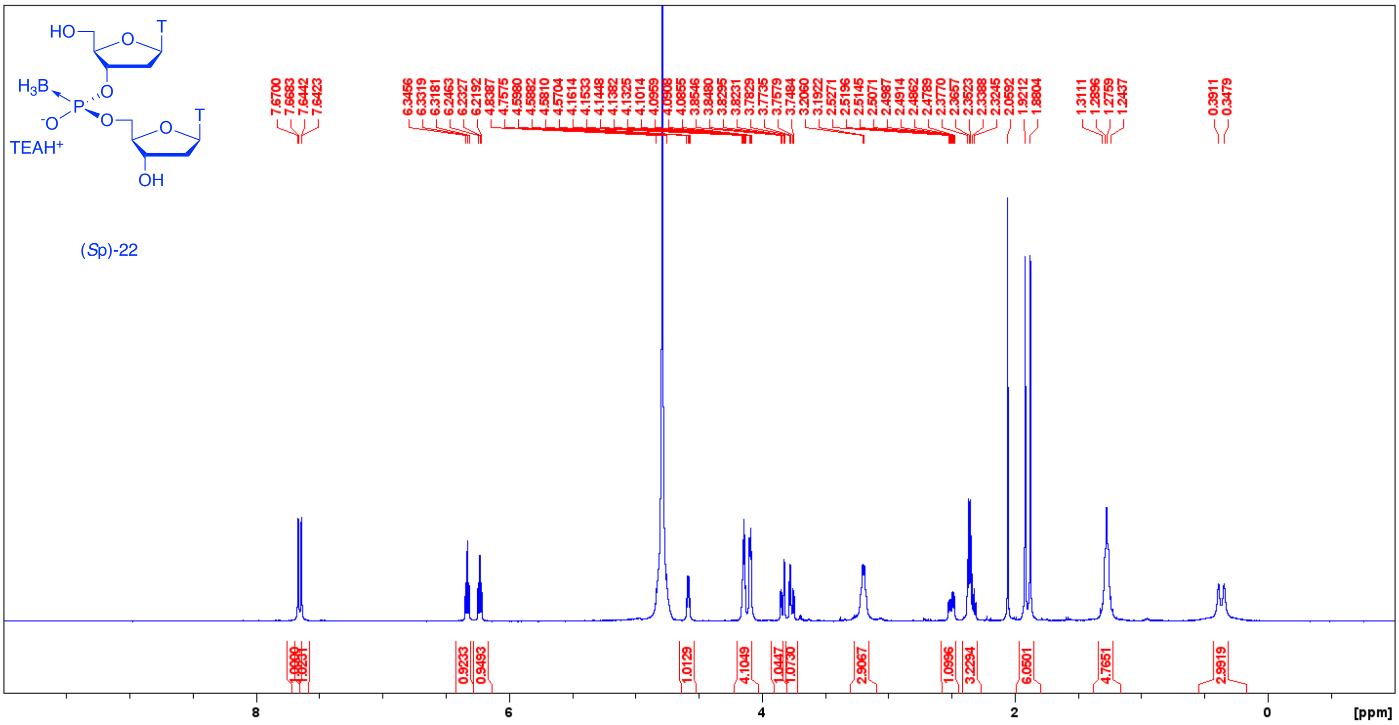

Figure S9 ^{1}H NMR spectrum of (*S*p)-22 (500 MHz, D_2O)

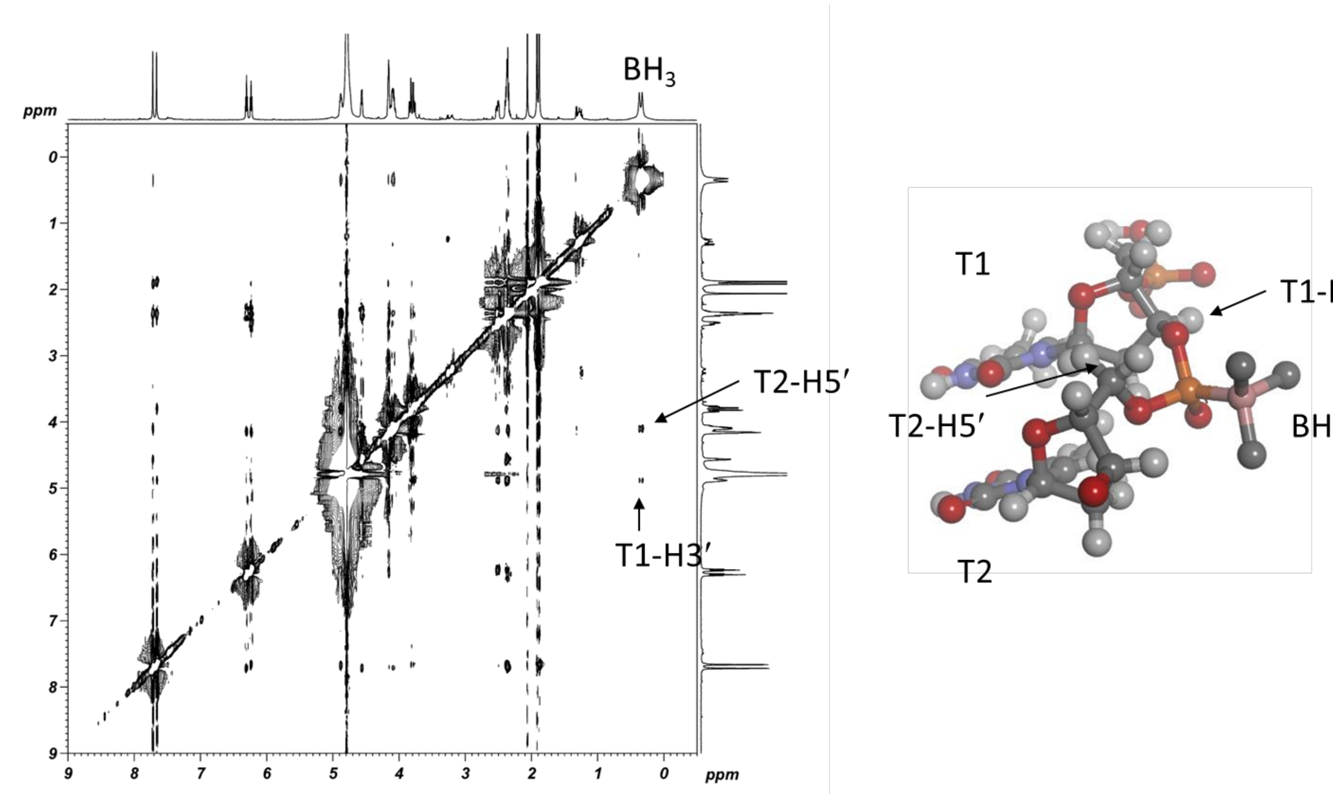

Figure S10 NOESY spectrum (*R*p)-PB diester ((*R*p)-22, ^{11}B decoupling). The molecular model was created by BIOVIA Discovery Studio Visualizer (Dassault Systemes S.E.) based on the DNA structure of B-type helix.

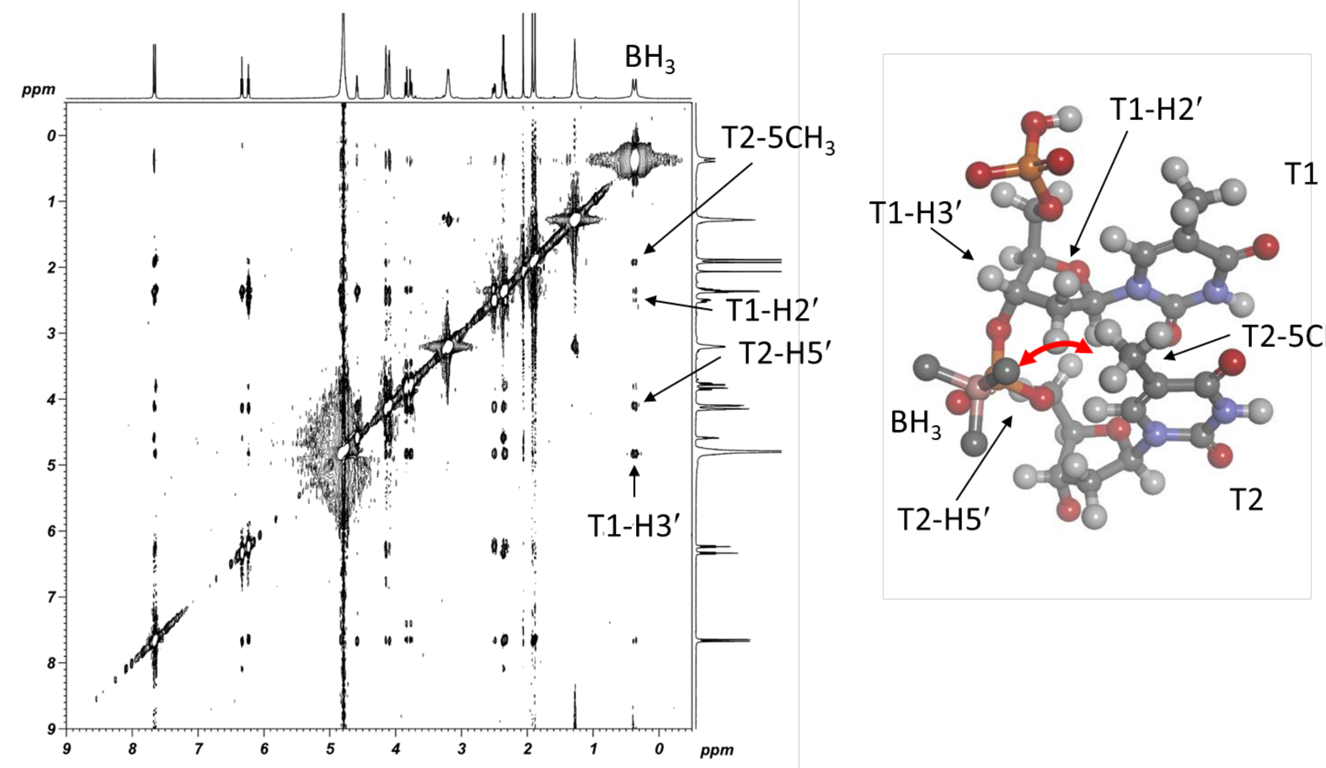

Figure S11 NOESY spectrum (*S*p)-PB diester ((*S*p)-22, ^{11}B decoupling). The molecular model was created by BIOVIA Discovery Studio Visualizer (Dassault Systemes S.E.) based on the DNA structure of B-type helix. The characteristic NOE is shown on the model with a red double-headed arrow.

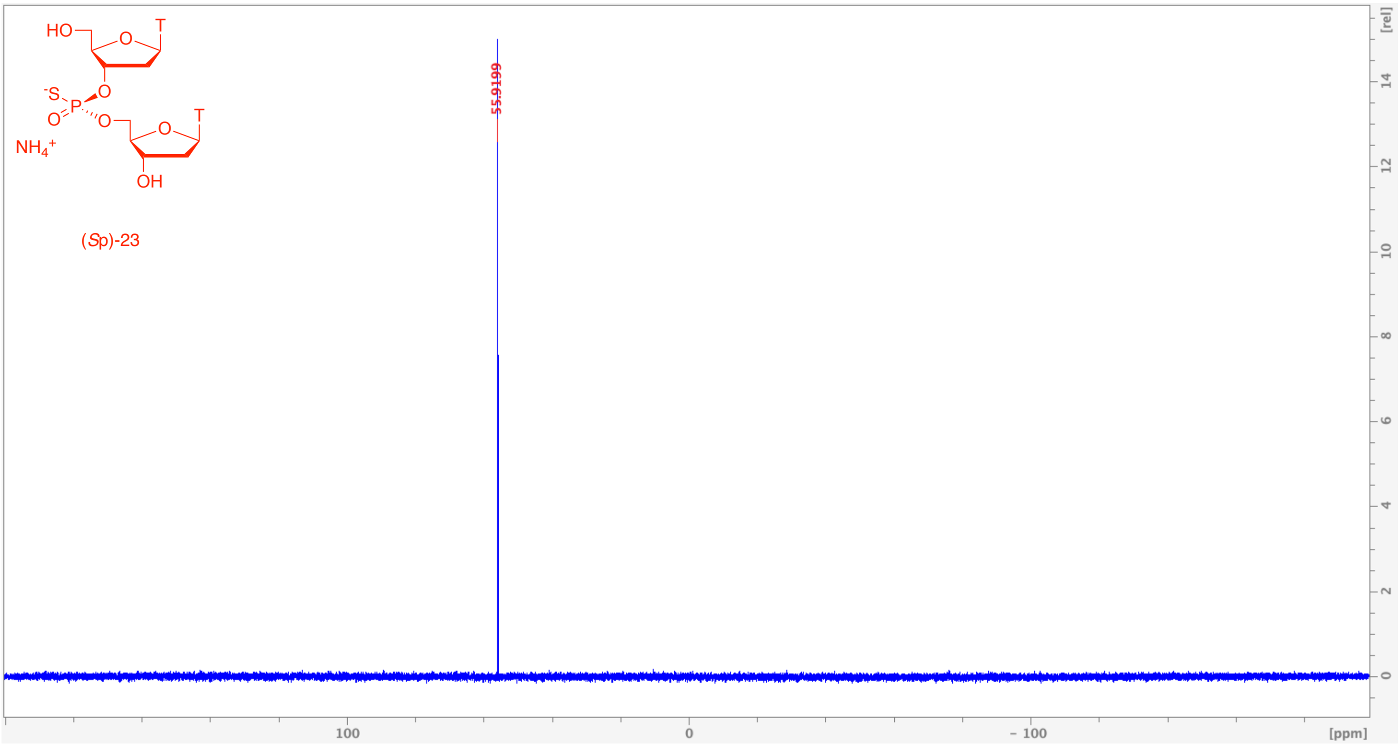

Figure S12 ^{31}P NMR spectrum of (*S*p)-23 (243 MHz, D_2O)

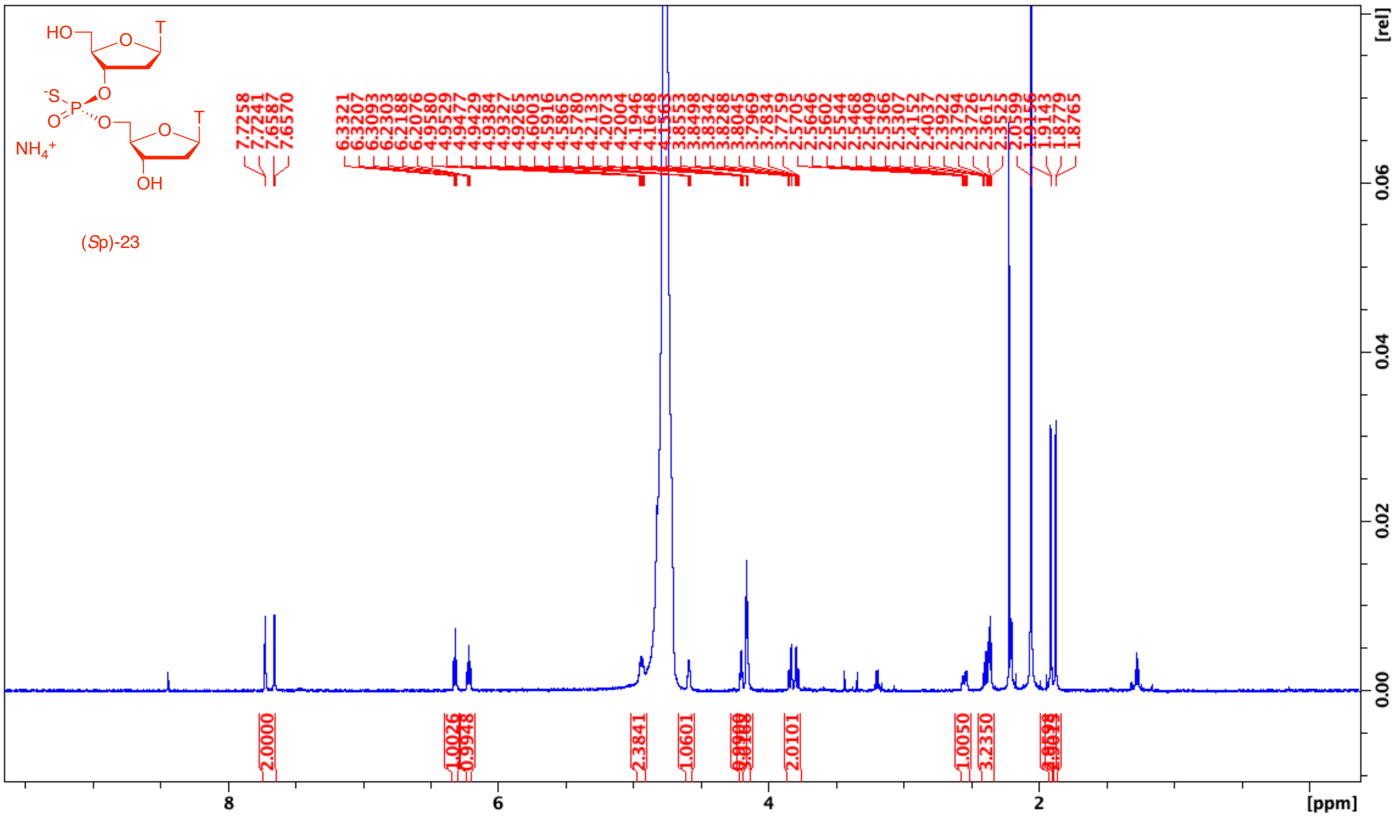

Figure S13 ^{1}H NMR spectrum of (*S*p)-23 (600 MHz, D_2O)

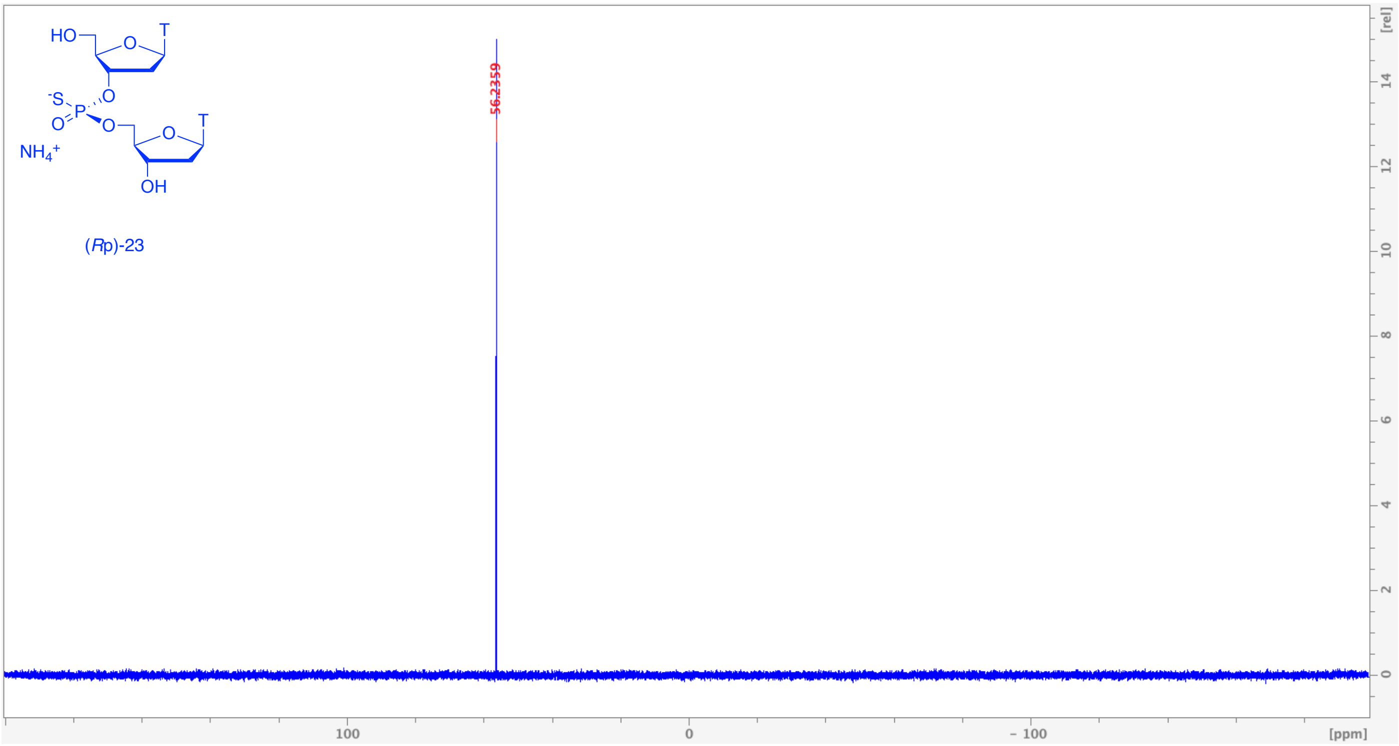

Figure S14 ^{31}**P NMR spectrum of (*R*p)-23 (243 MHz, D$_2$O)**

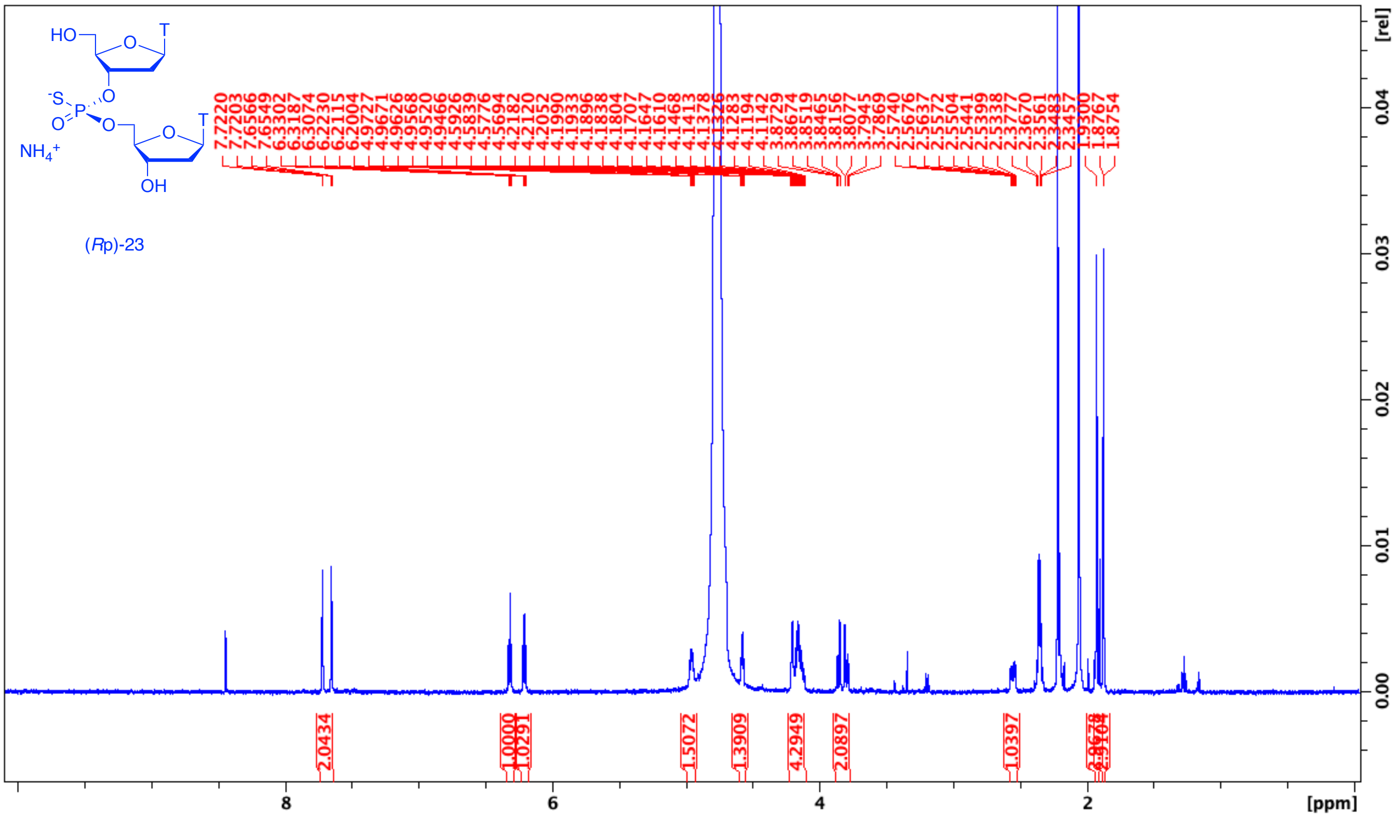

Figure S15 ^{1}**H NMR spectrum of (*R*p)-23 (600 MHz, D$_2$O)**

RP-HPLC profile of phosphorothioate tetramer (d($C_{PS}A_{PS}G_{PS}T$))

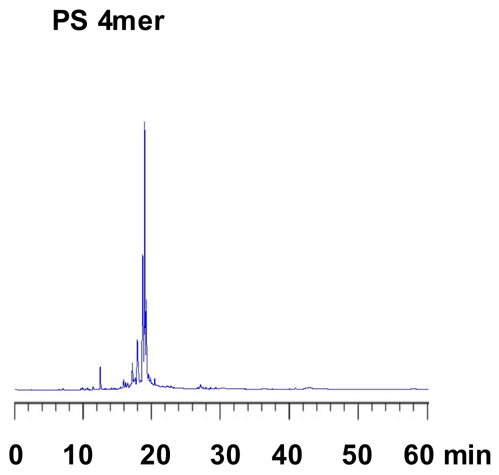

Figure S16 RP-HPLC was performed with a linear gradient of 0%–60% acetonitrile for 60 min in 0.1 M triethylammonium acetate buffer (pH 7.0) at 30 °C at a flow rate of 0.5 mL/min using a C18 column.

RP-HPLC profiles of PS dodecamer (d($G_{PS}C_{PS}A_{PS}T_{PS}T_{PS}G_{PS}G_{PS}T_{PS}A_{PS}T_{PS}T_{PS}C$))

Crude mixture of PS dodecamer

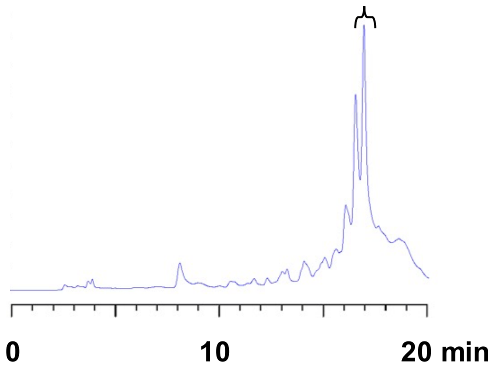

Purified PS 12mer

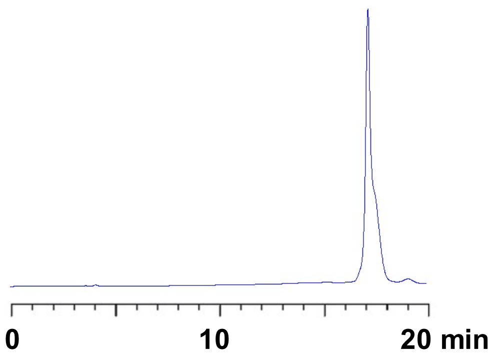

Figure S17 RP-HPLC was performed with a linear gradient of 5%–40% MeOH in 400 mM HFIP, 8 mM TEA for 20 min at 60 °C at a flow rate of 0.5 mL/min using a C18 column.

RP-HPLC profiles of PS/PO dodecamer ($d(G_{PO}C_{PS}A_{PO}T_{PS}T_{PO}G_{PS}G_{PO}T_{PS}A_{PO}T_{PS}T_{PO}C)$)

Crude mixture of PS/PO 12mer

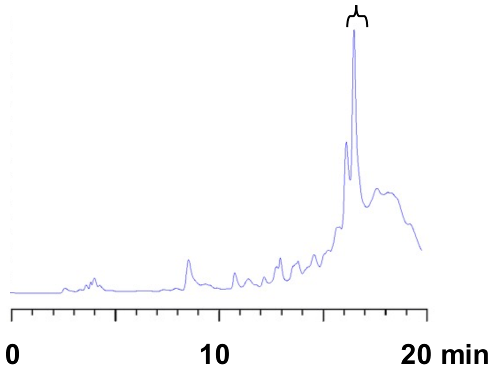

Purified PS/PO 12mer

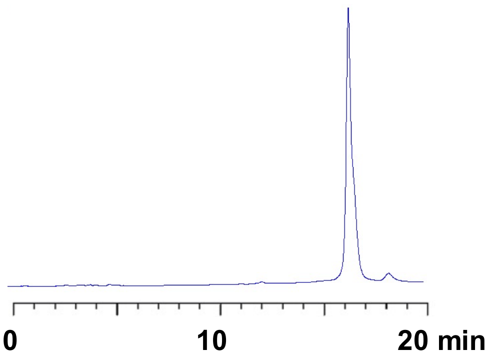

Figure S18 RP-HPLC was performed with a linear gradient of 5%–40% MeOH in 400 mM HFIP, 8 mM TEA for 20 min at 60 °C at a flow rate of 0.5 mL/min using a C18 column.

NMR spectra of new compounds

^{1}H NMR (400 MHz, $CDCl_3$)

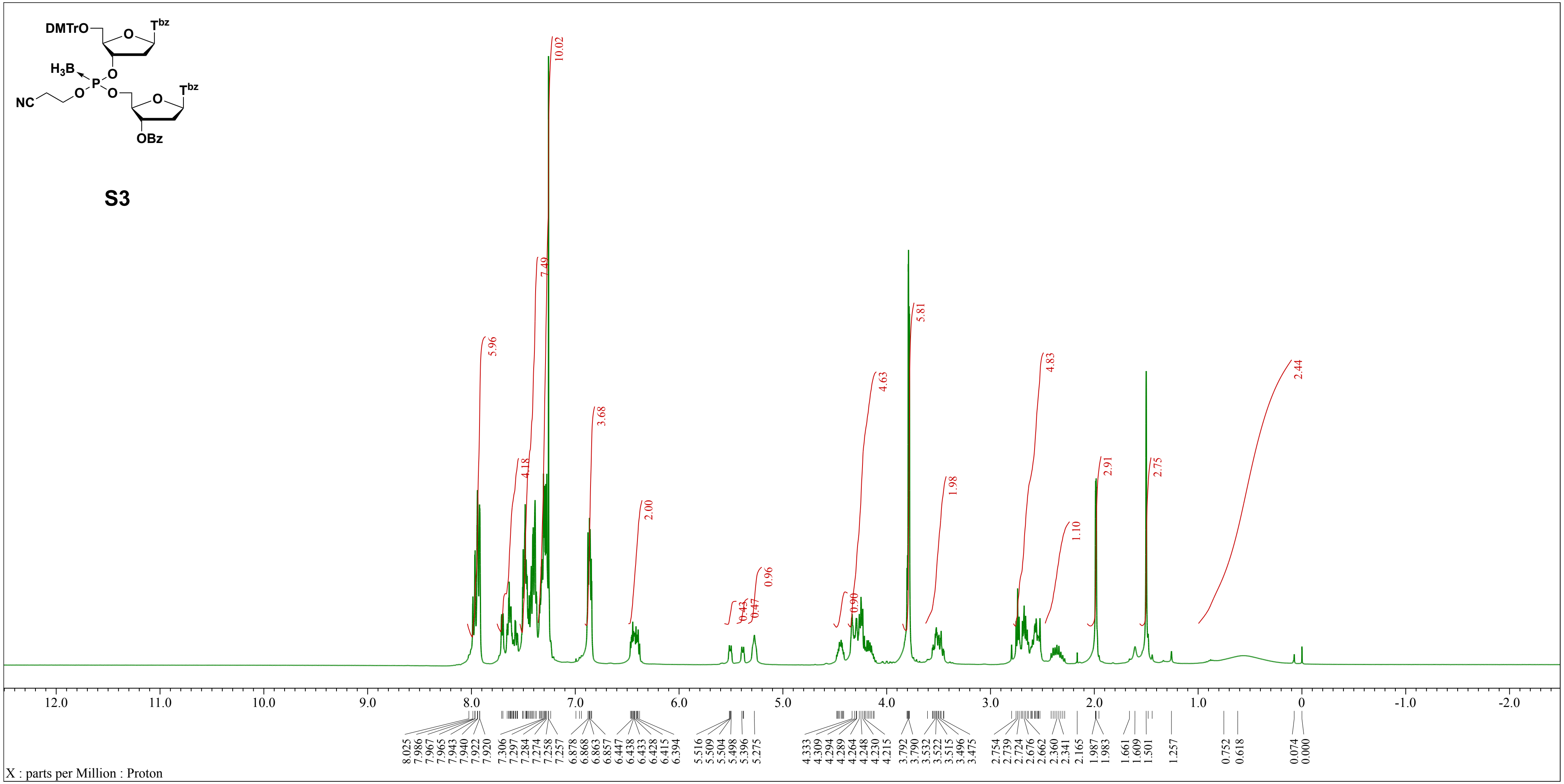

$^{13}C\{^1H\}$ NMR (101 MHz, $CDCl_3$)

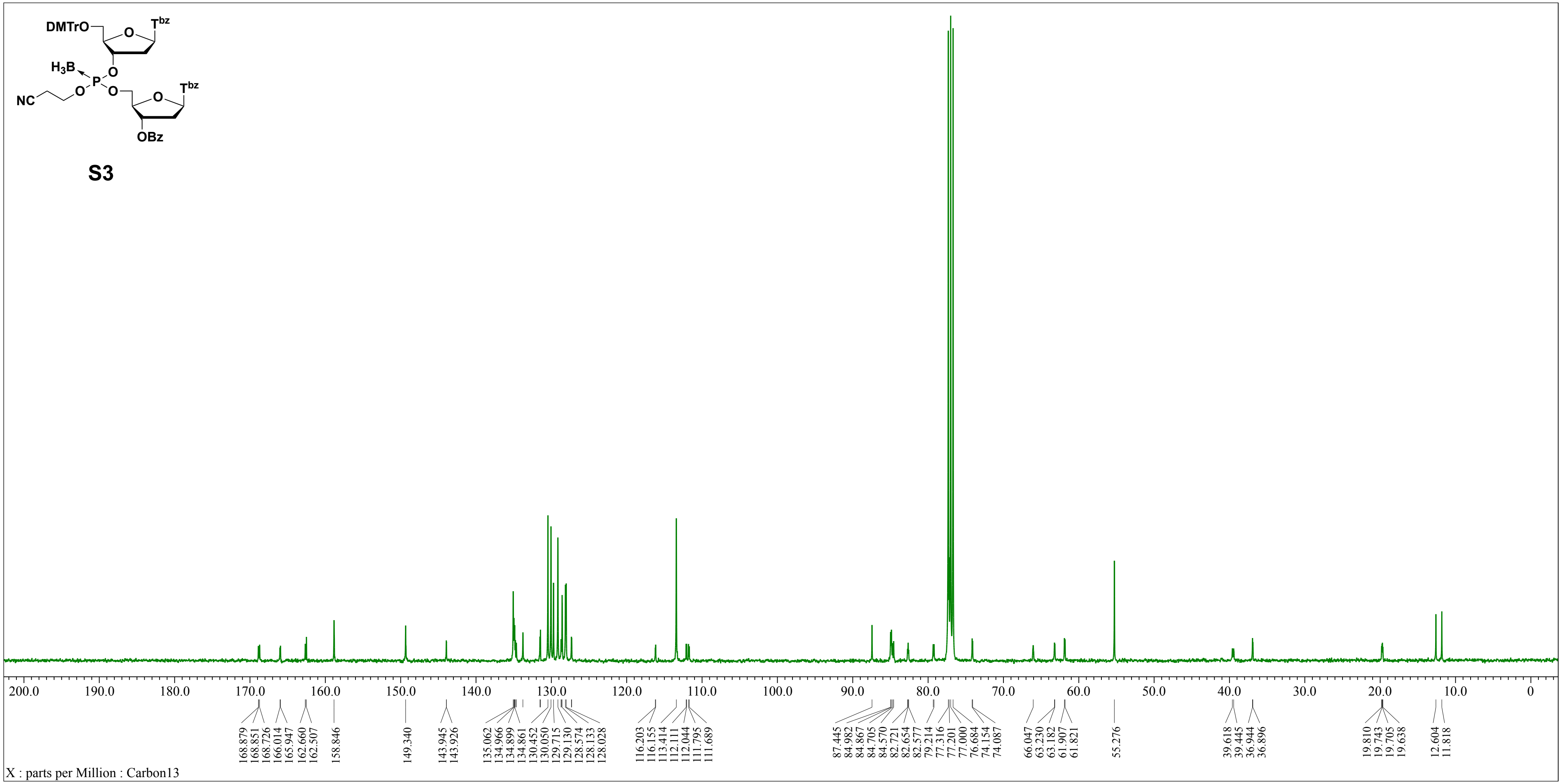

^{31}P NMR (162 MHz, $CDCl_3$)

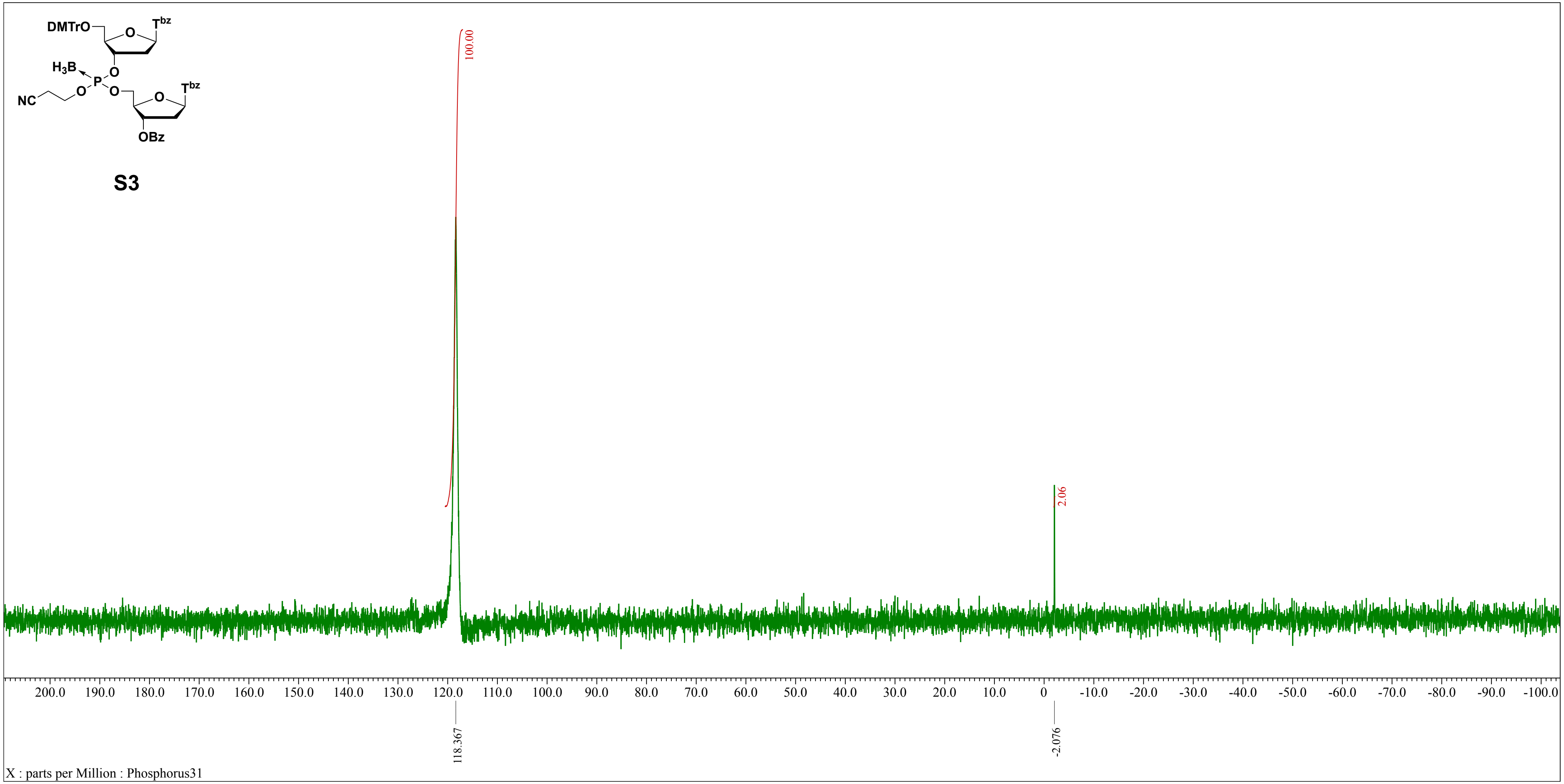

^{1}H NMR (400 MHz, $CDCl_3$)

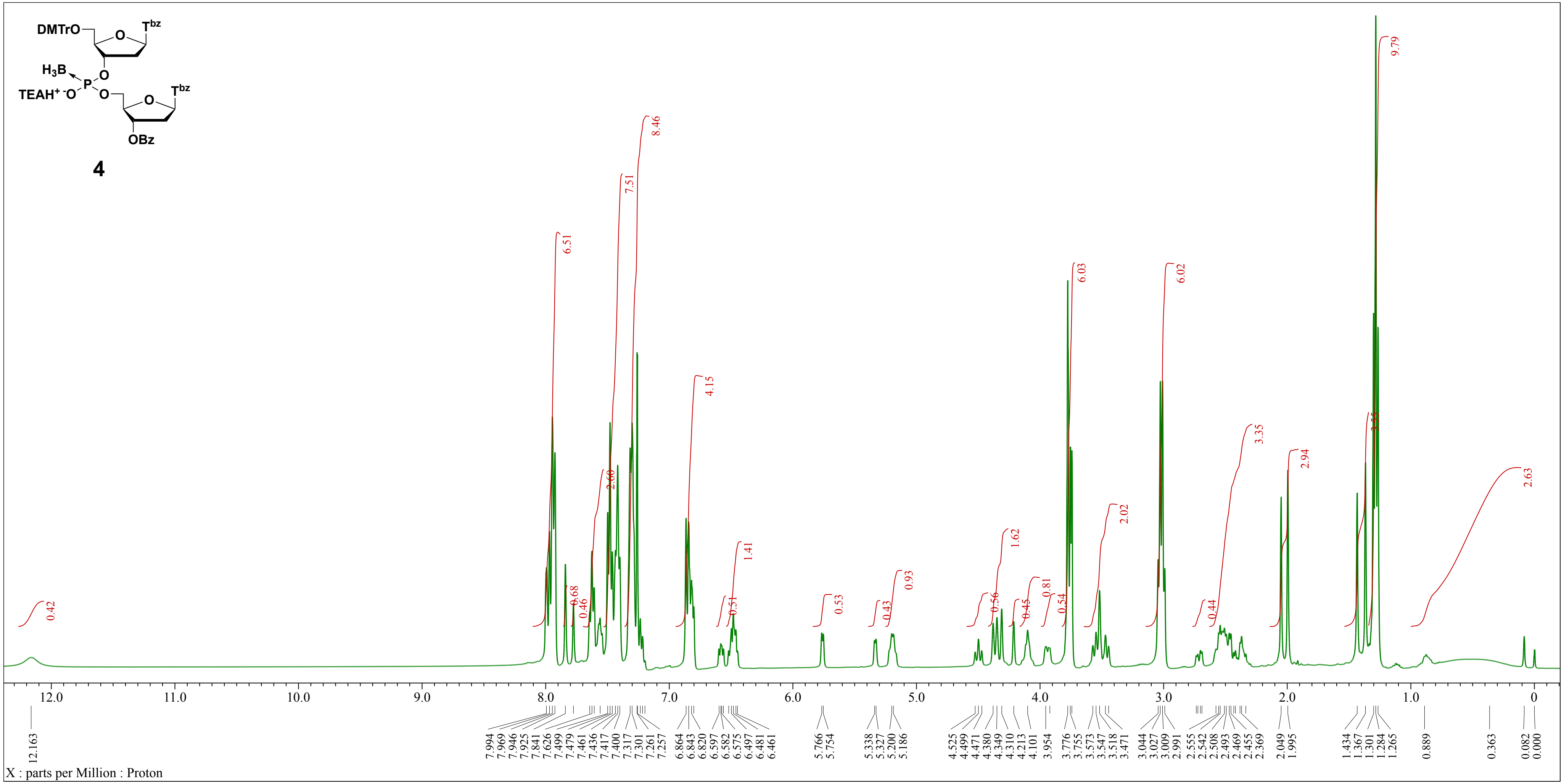

^{13}C {^{1}H} NMR (101 MHz, $CDCl_3$)

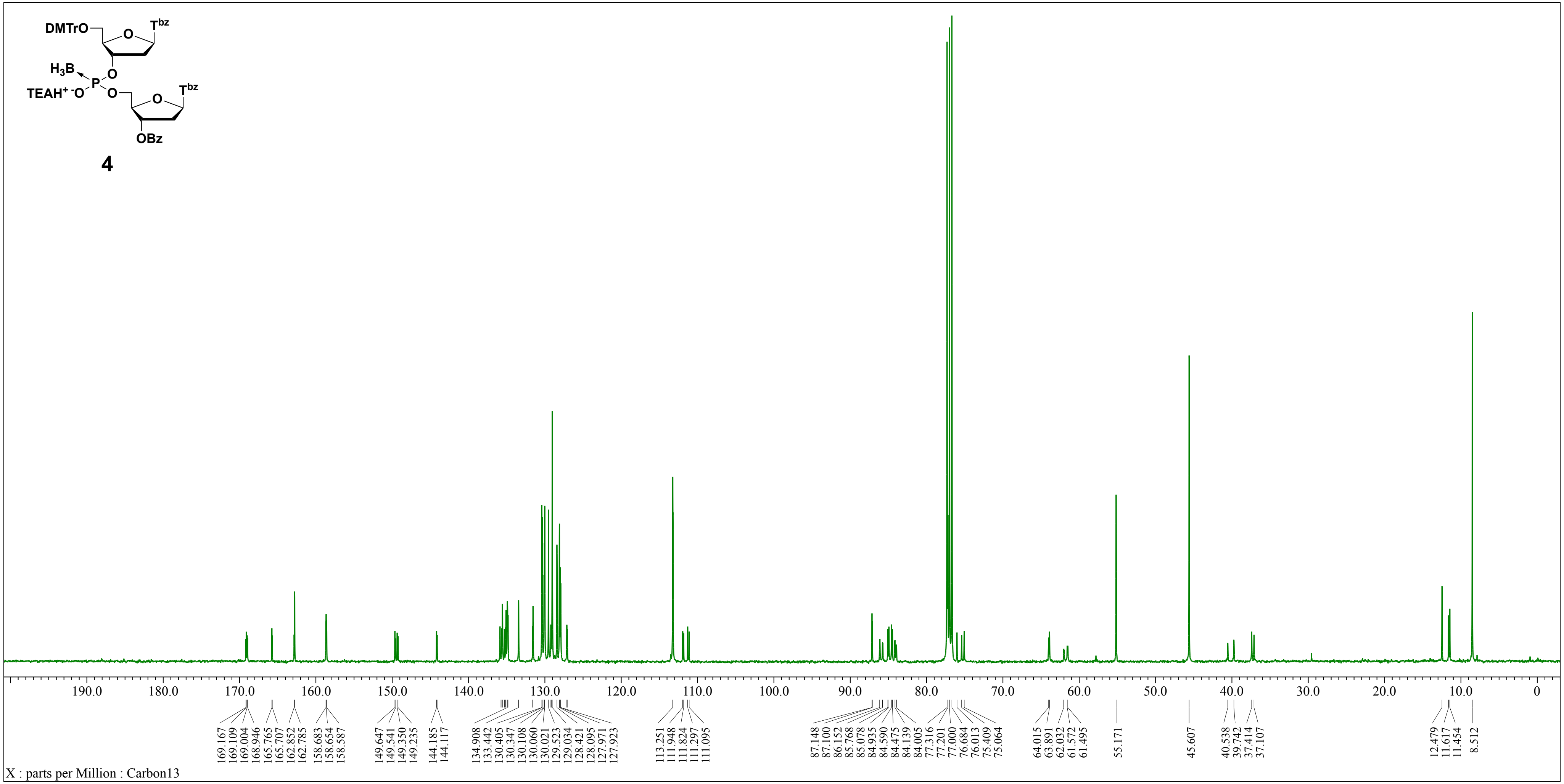

^{31}P NMR (162 MHz, $CDCl_3$)

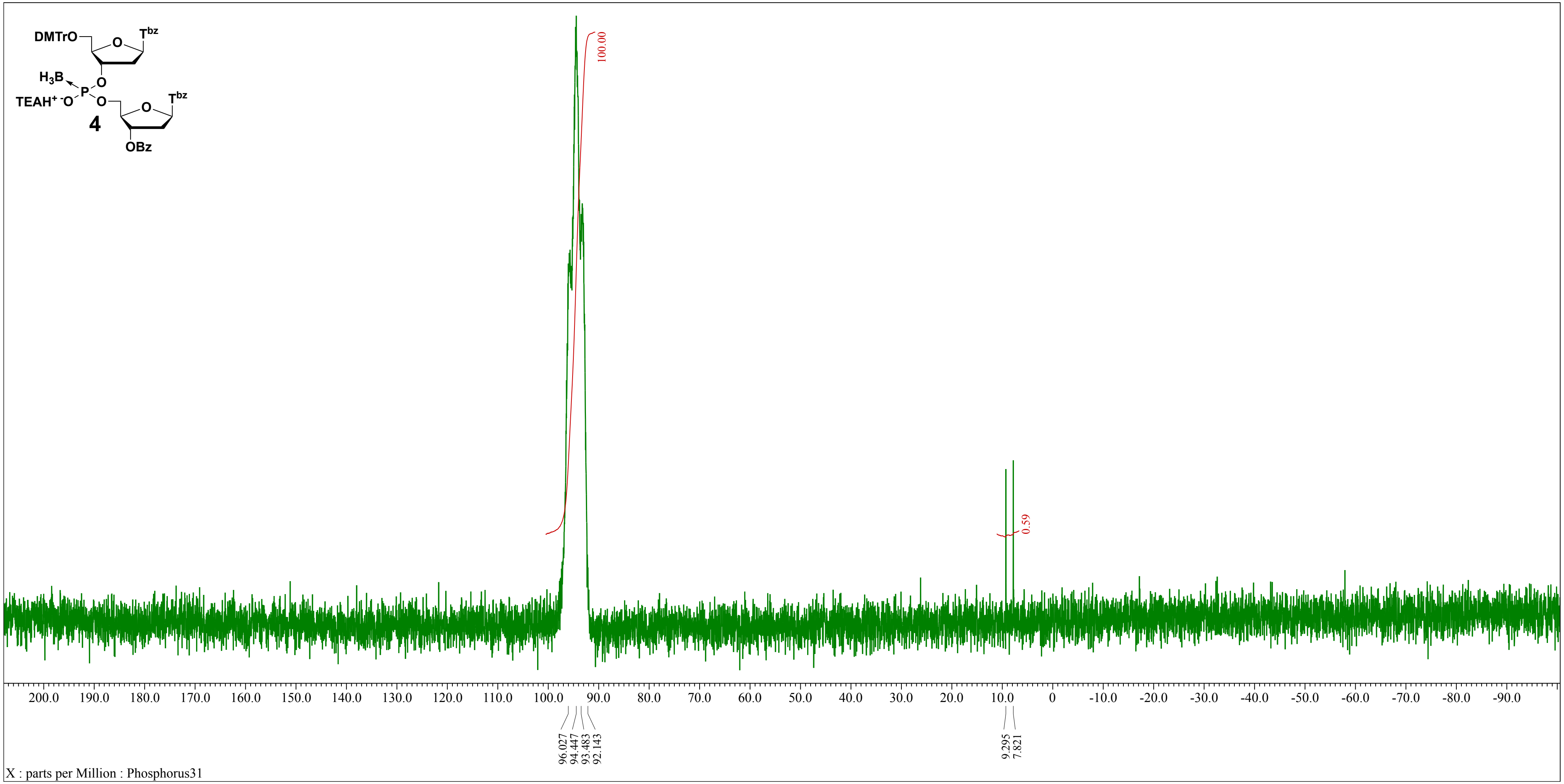

^{1}H NMR (400 MHz, $CDCl_3$)

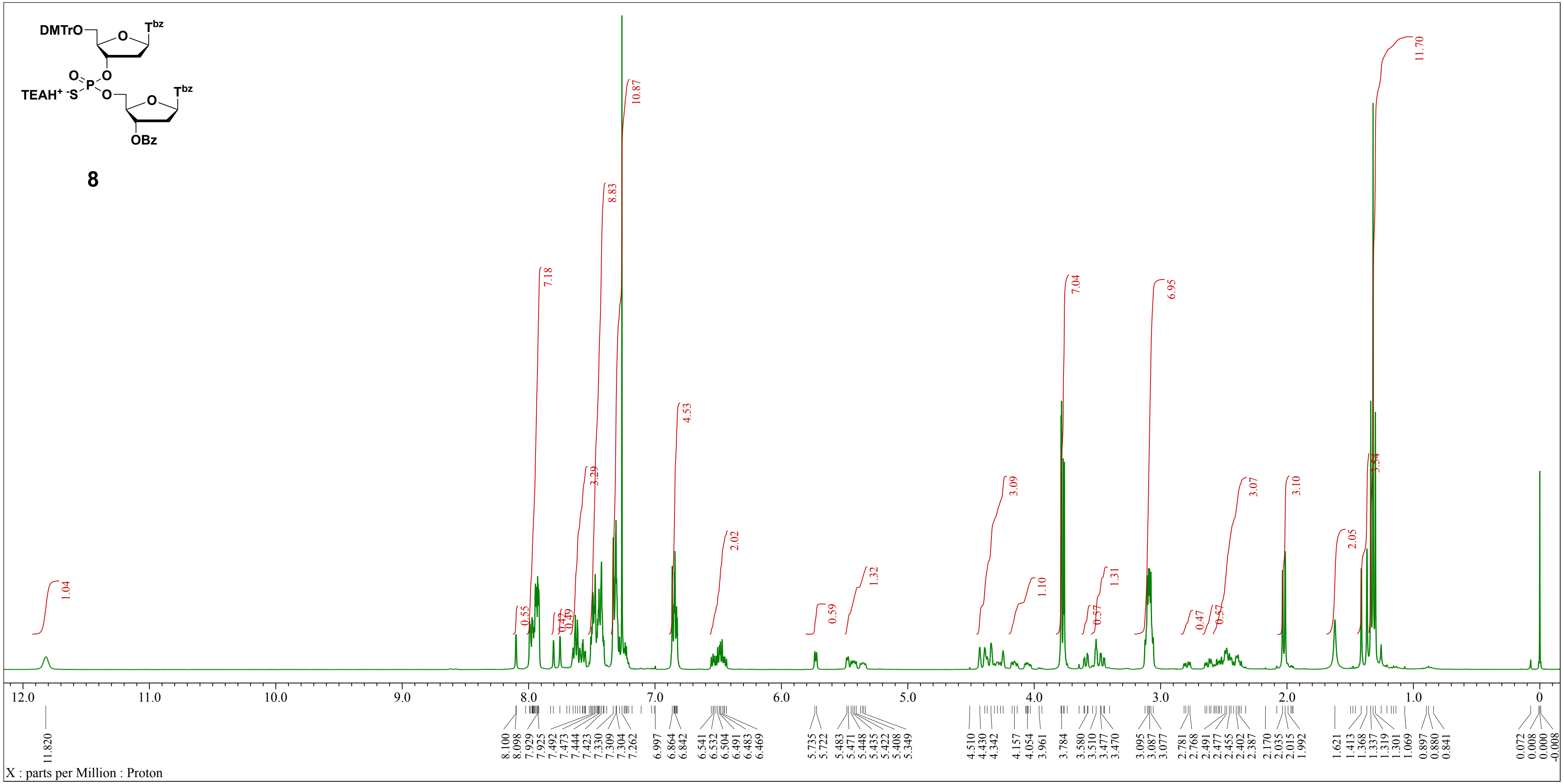

^{13}C {^{1}H} NMR (101 MHz, $CDCl_3$)

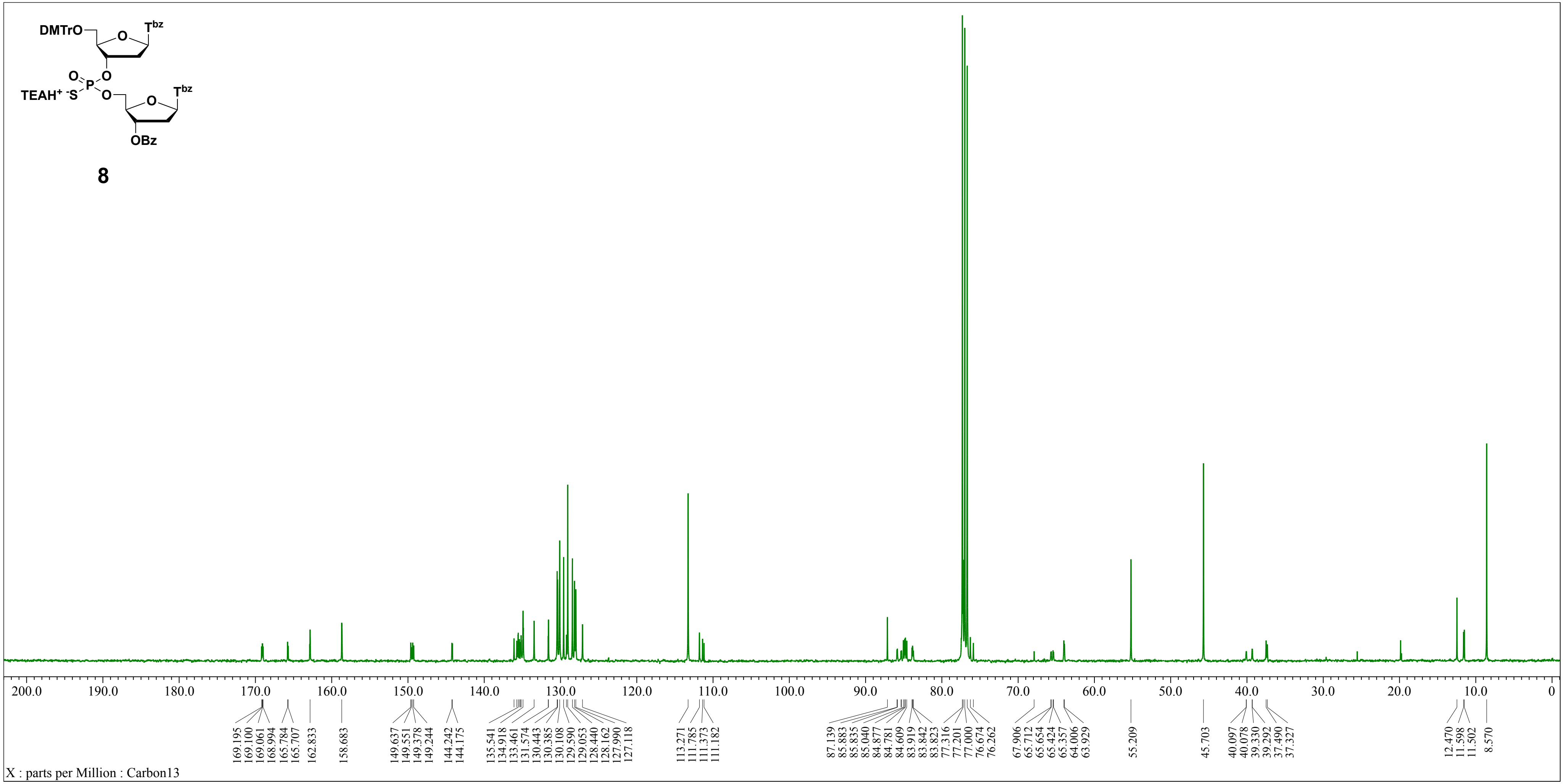

^{31}P NMR (162 MHz, CDCl$_3$)

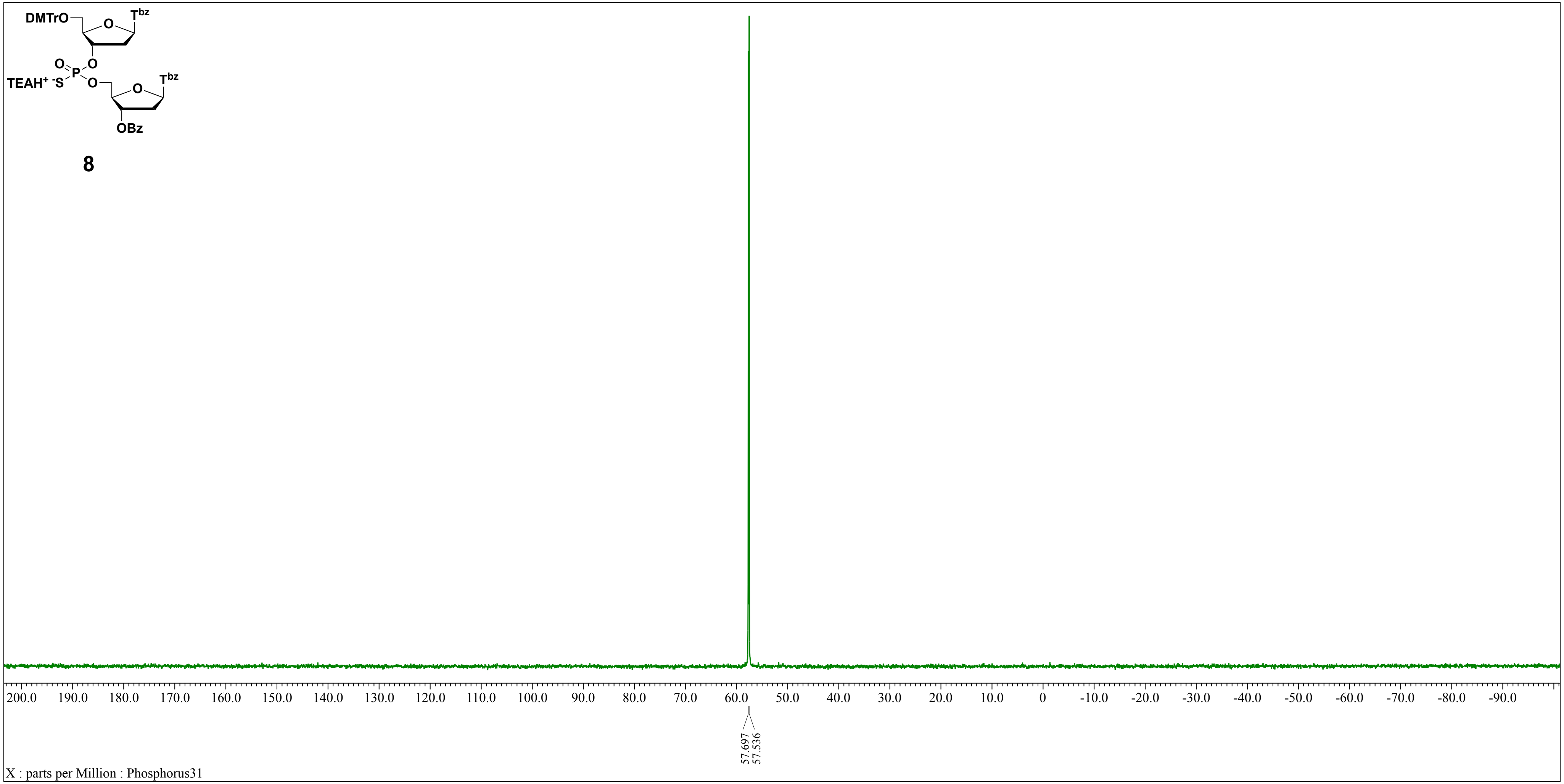

^{1}H NMR (400 MHz, $CDCl_3$)

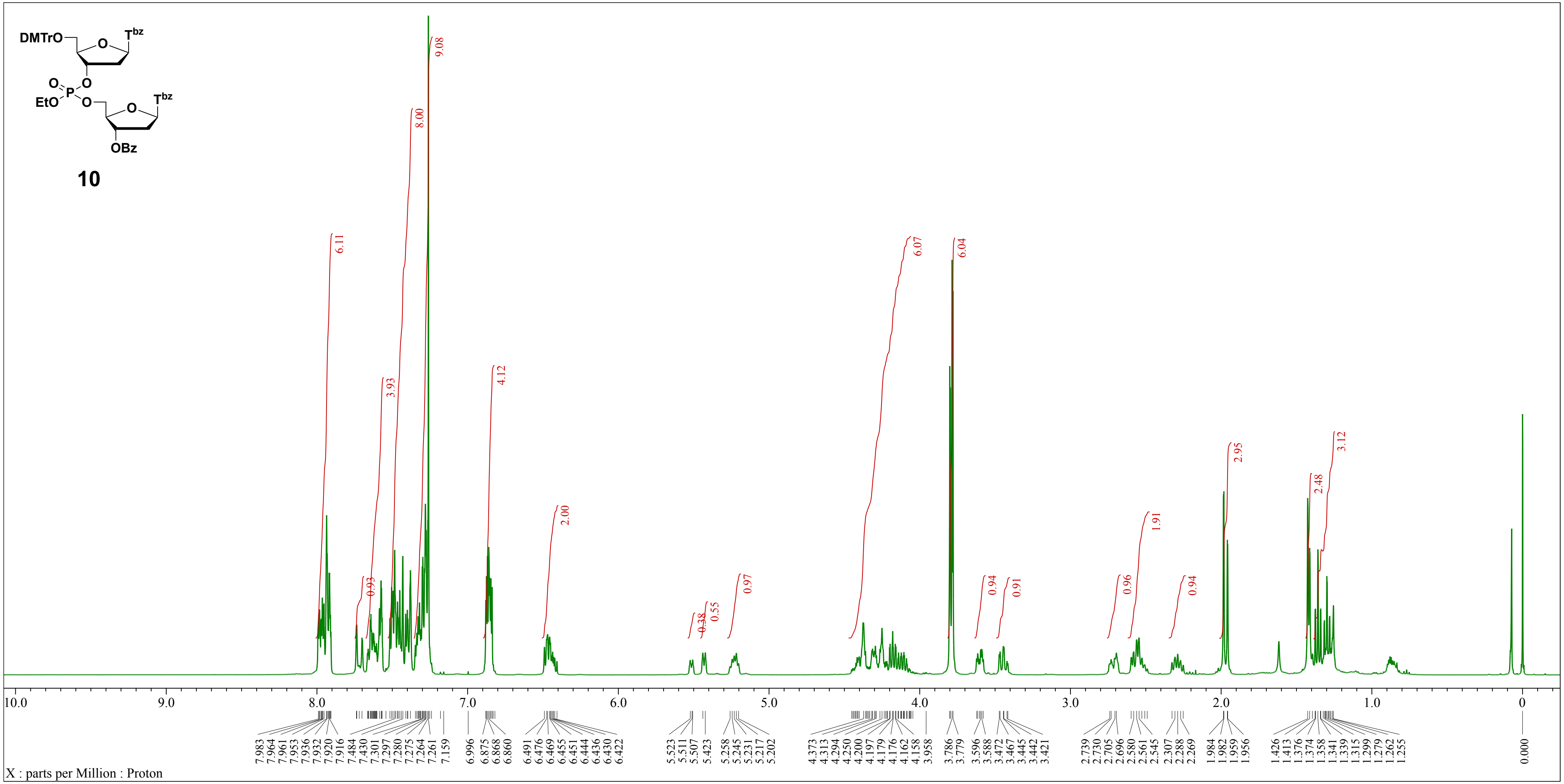

$^{13}C\ \{^1H\}$ NMR (101 MHz, $CDCl_3$)

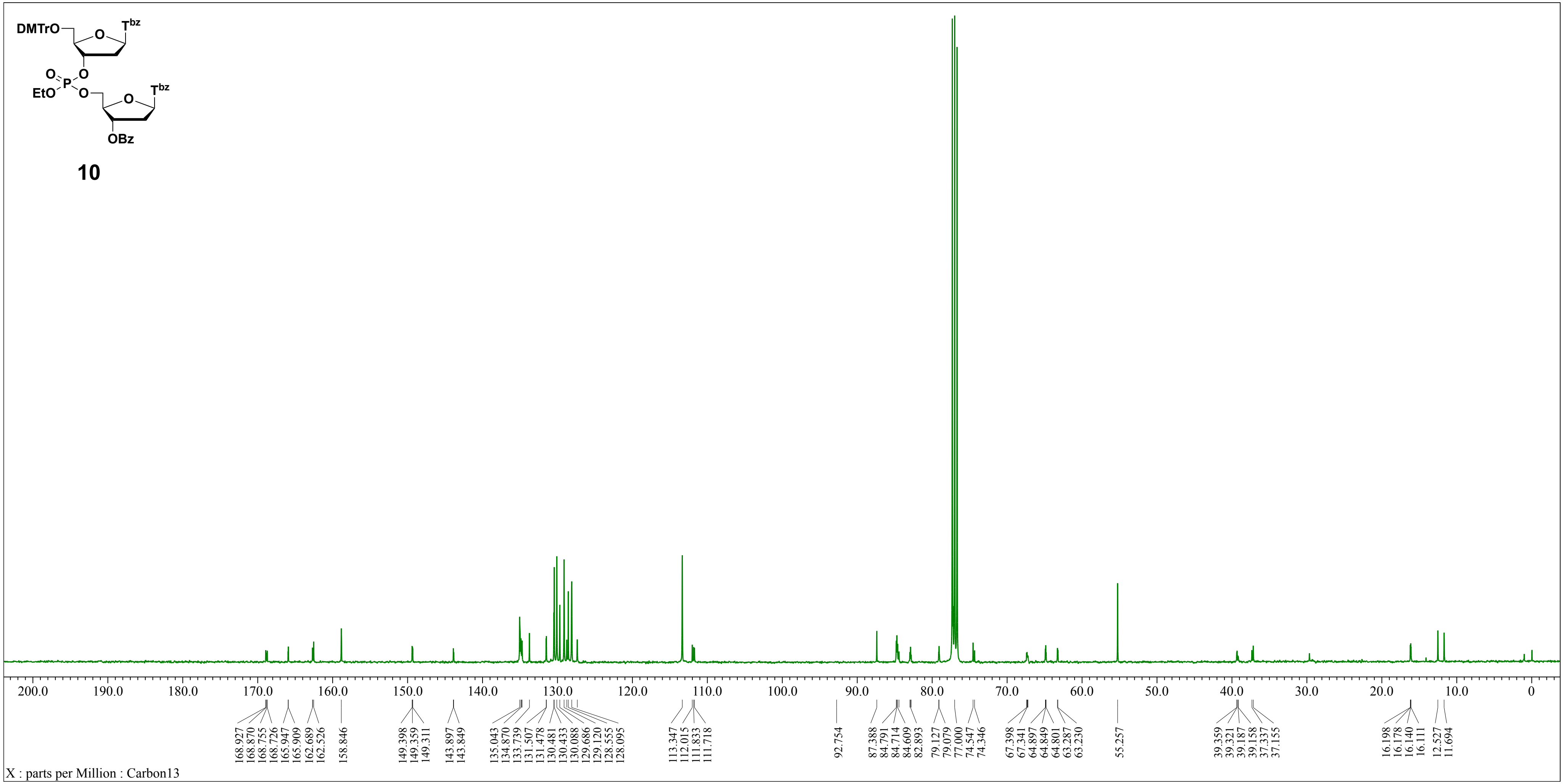

^{31}P NMR (162 MHz, $CDCl_3$)

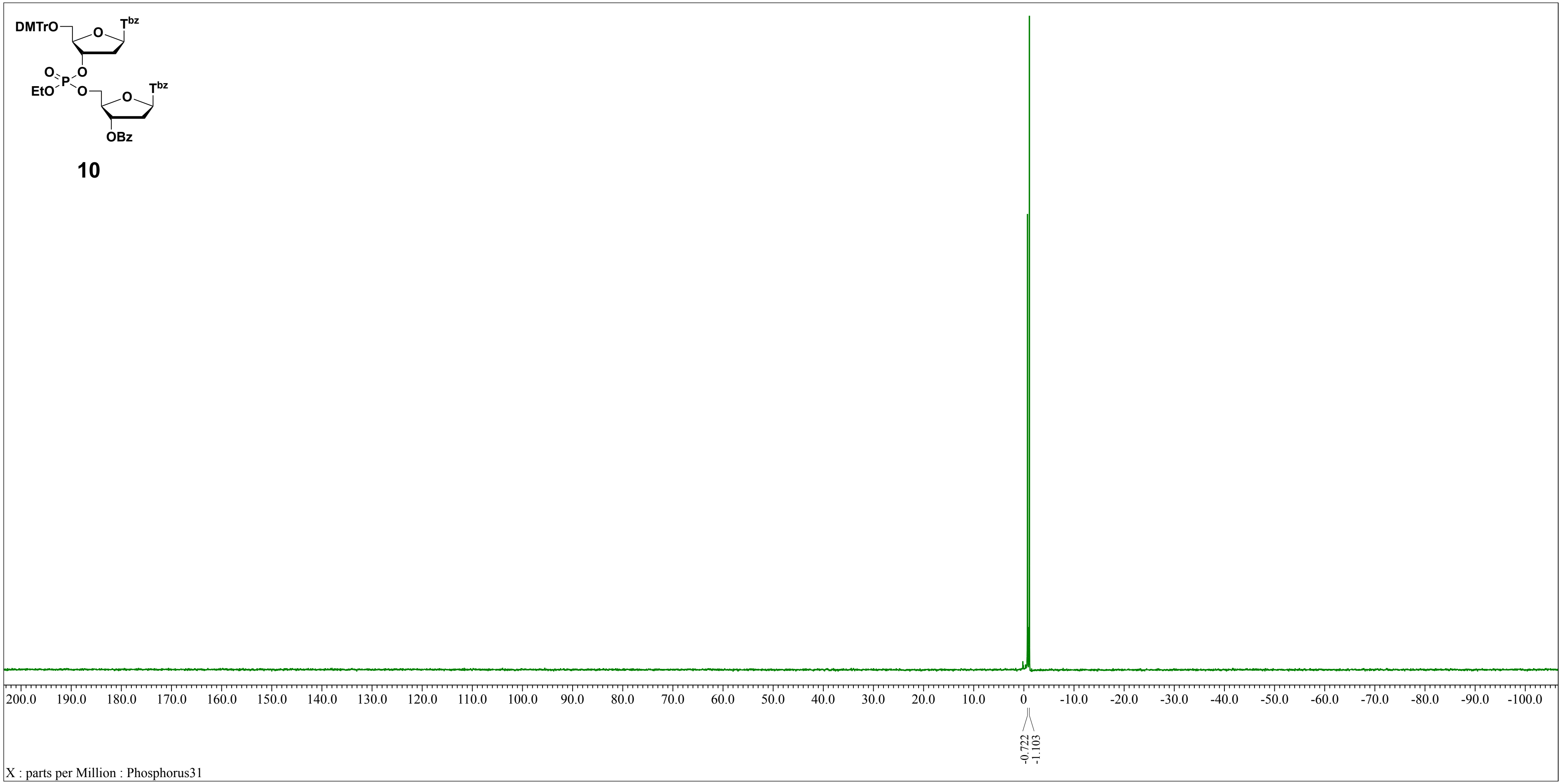

^{1}H NMR (400 MHz, $CDCl_3$)

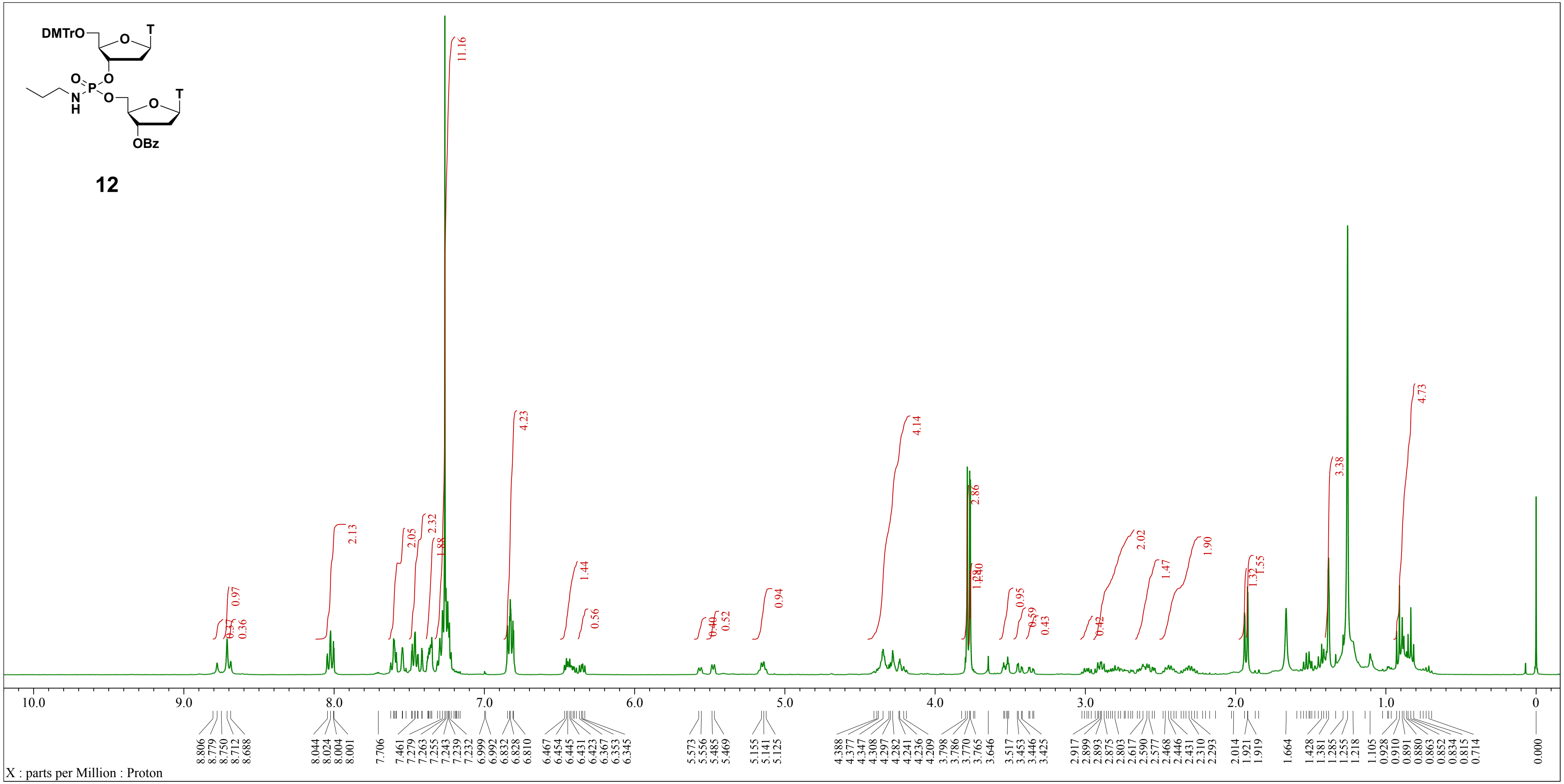

^{13}C {^{1}H} NMR (101 MHz, $CDCl_3$)

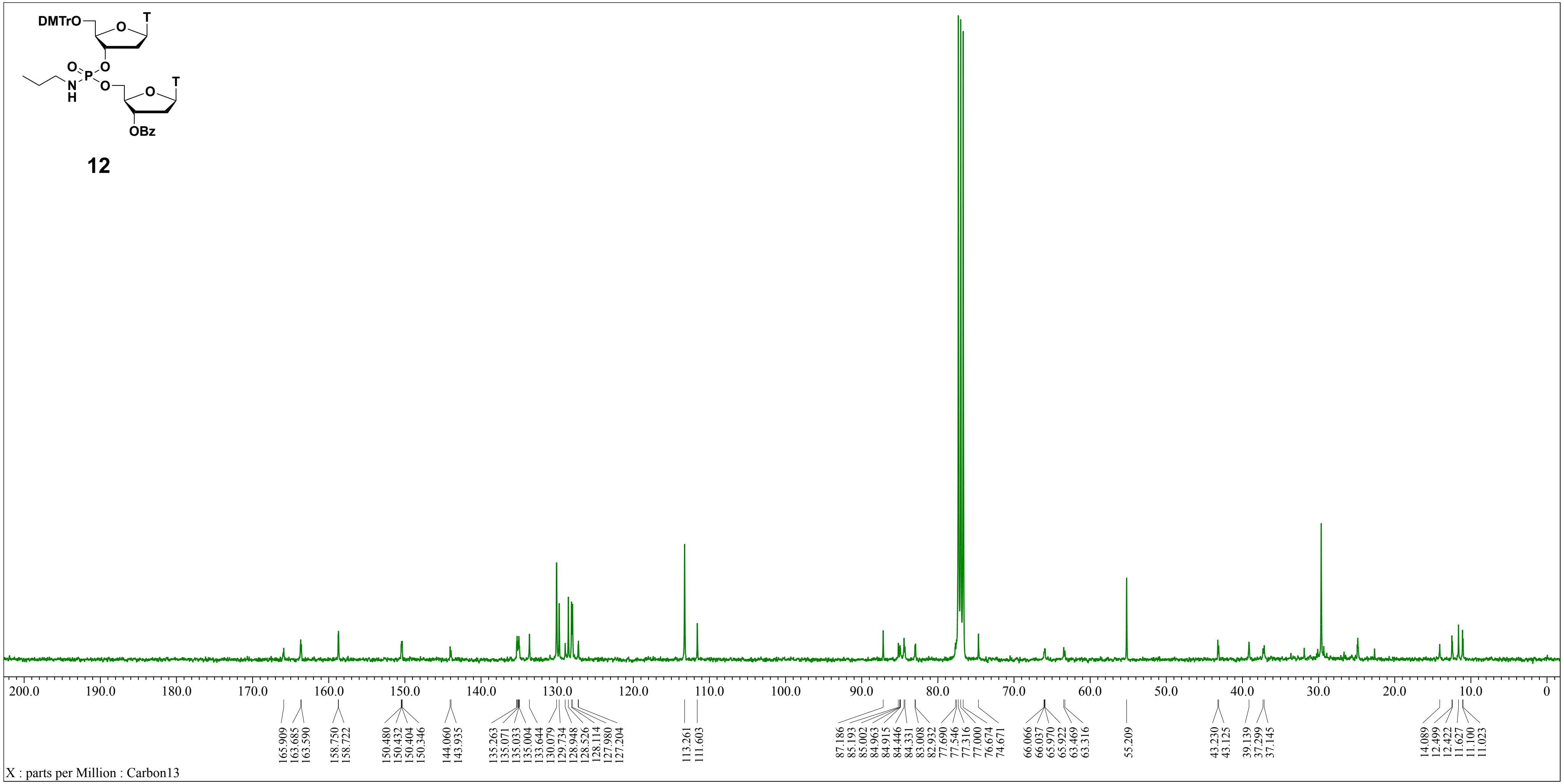

^{31}P NMR (162 MHz, $CDCl_3$)

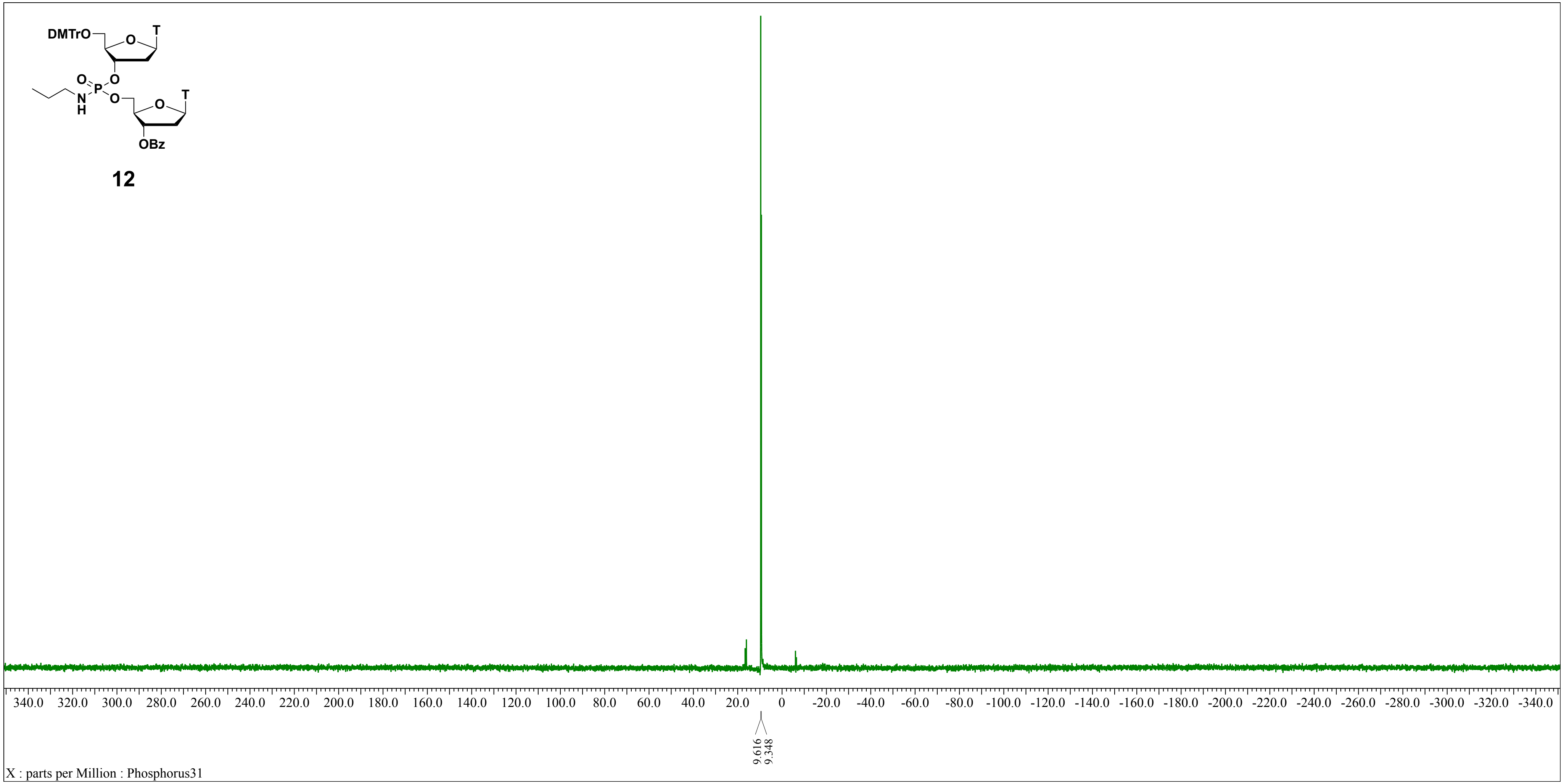

^{1}H NMR (400 MHz, $CDCl_3$)

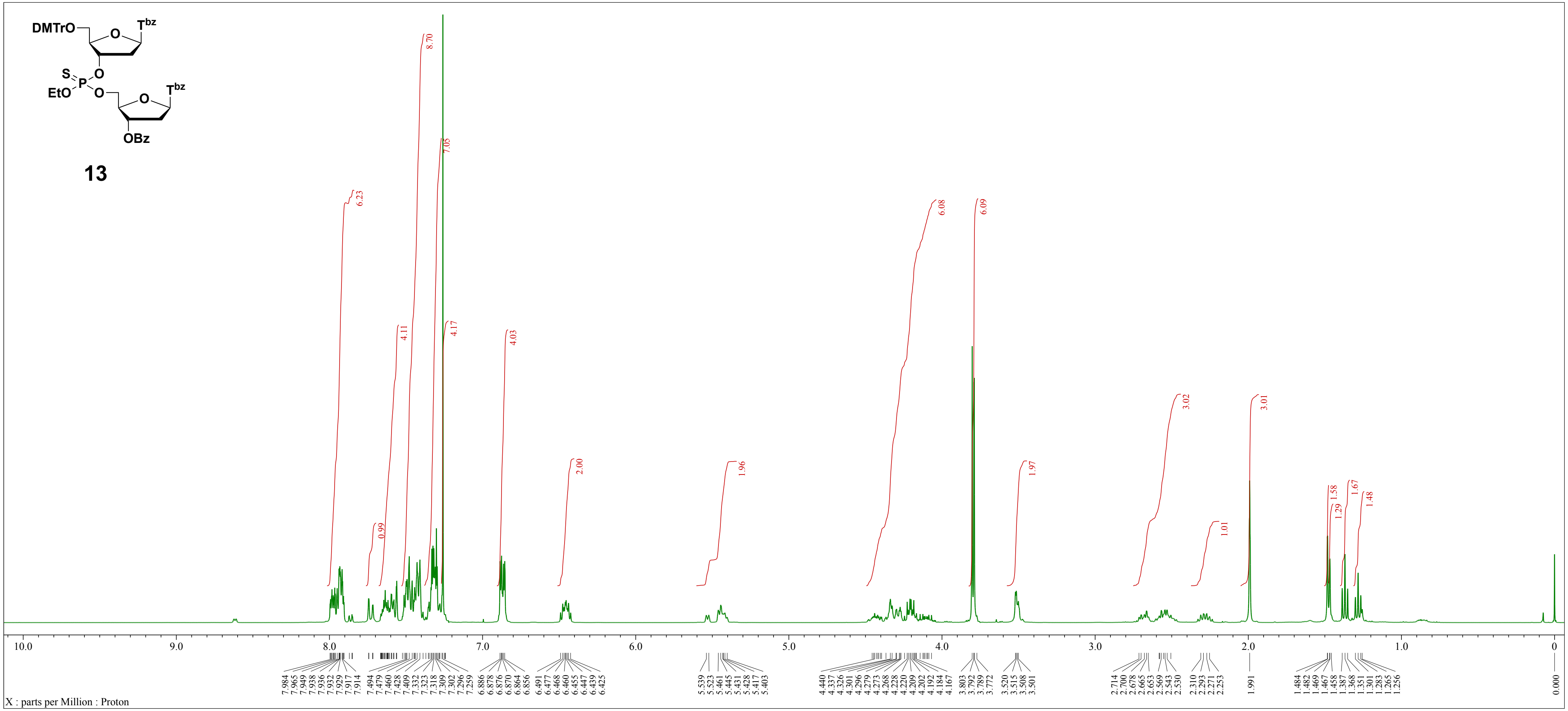

$^{13}C\ \{^{1}H\}$ NMR (101 MHz, $CDCl_3$)

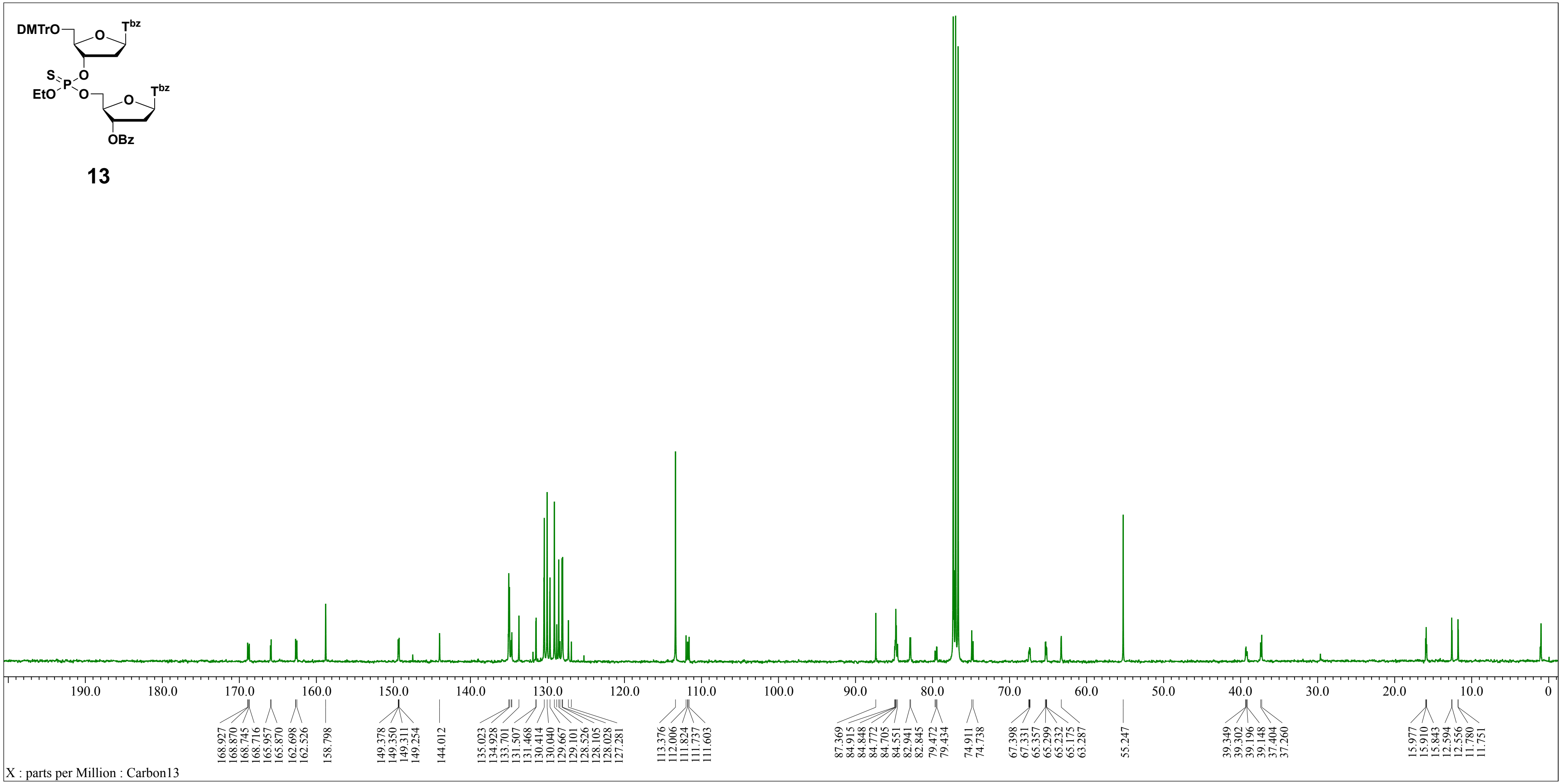

^{31}P NMR (162 MHz, $CDCl_3$)

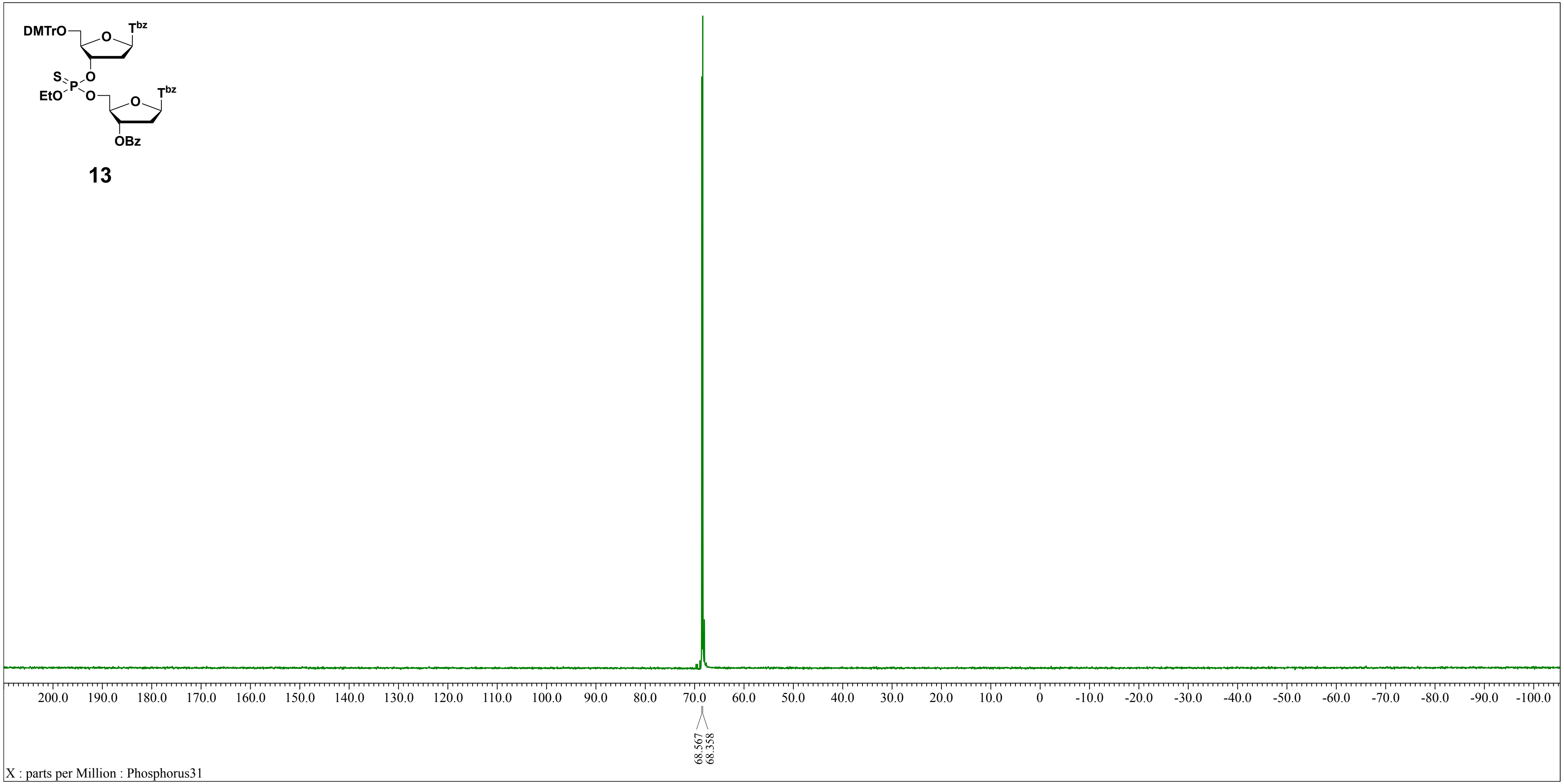

^{1}H NMR (400 MHz, $CDCl_3$)

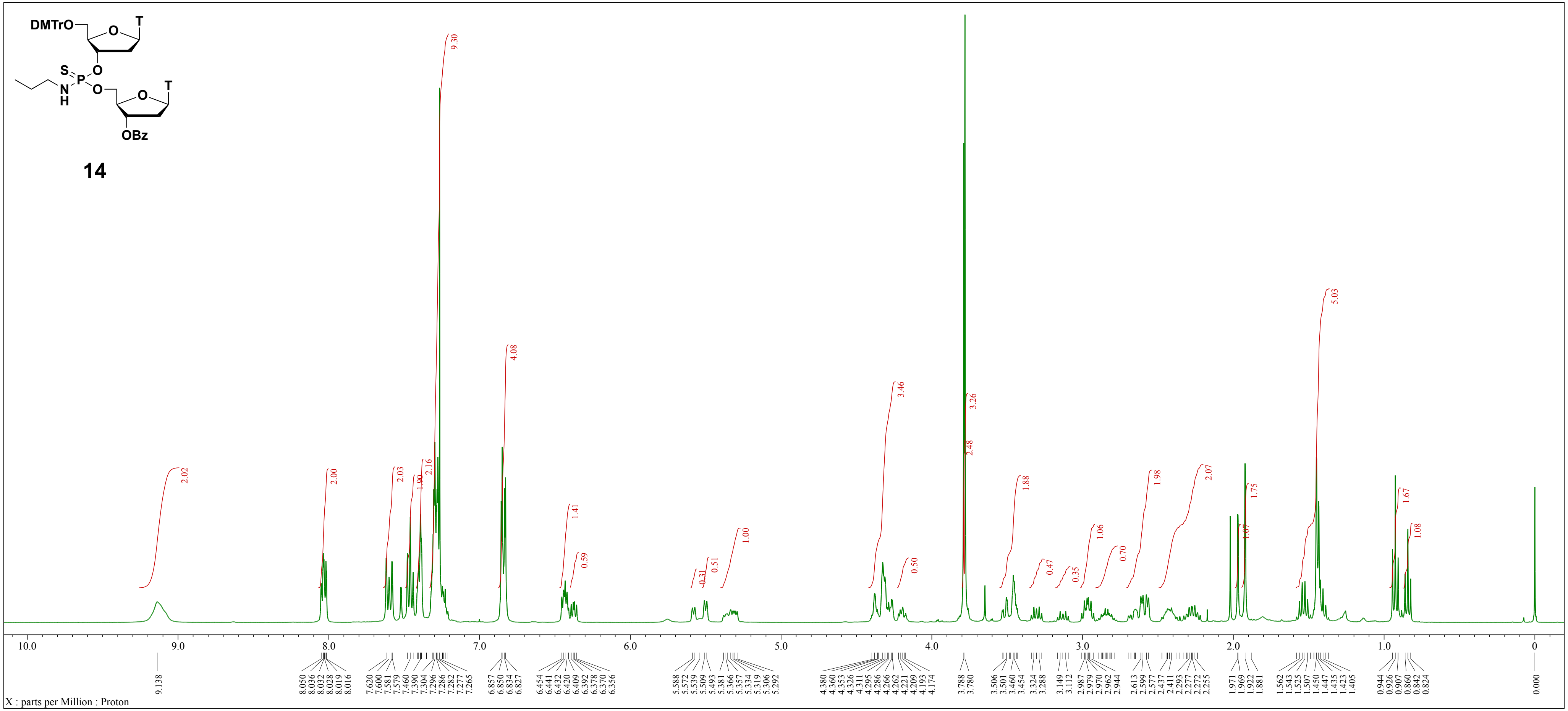

$^{13}C\ \{^1H\}$ NMR (101 MHz, $CDCl_3$)

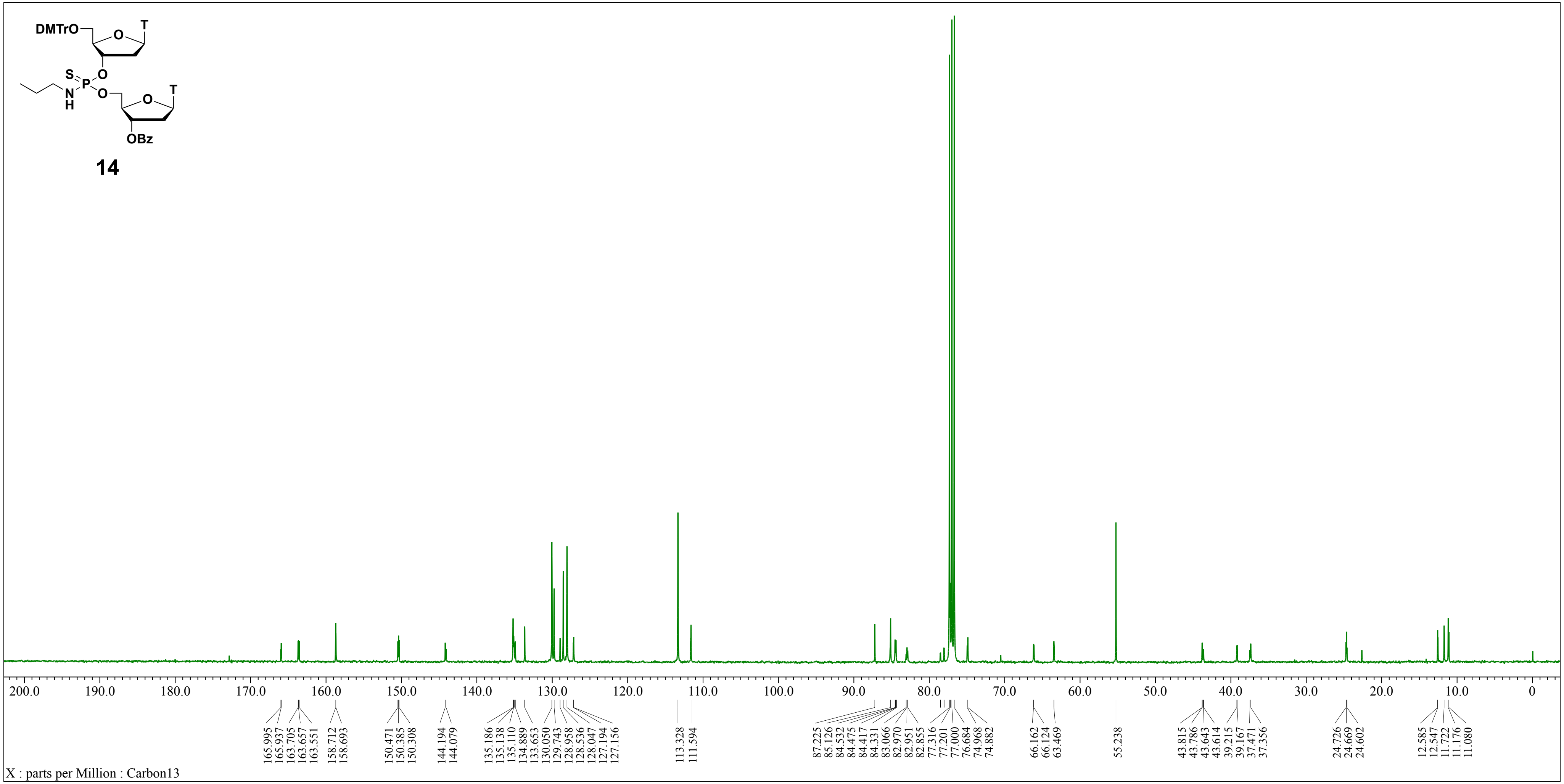

^{31}P NMR (162 MHz, $CDCl_3$)

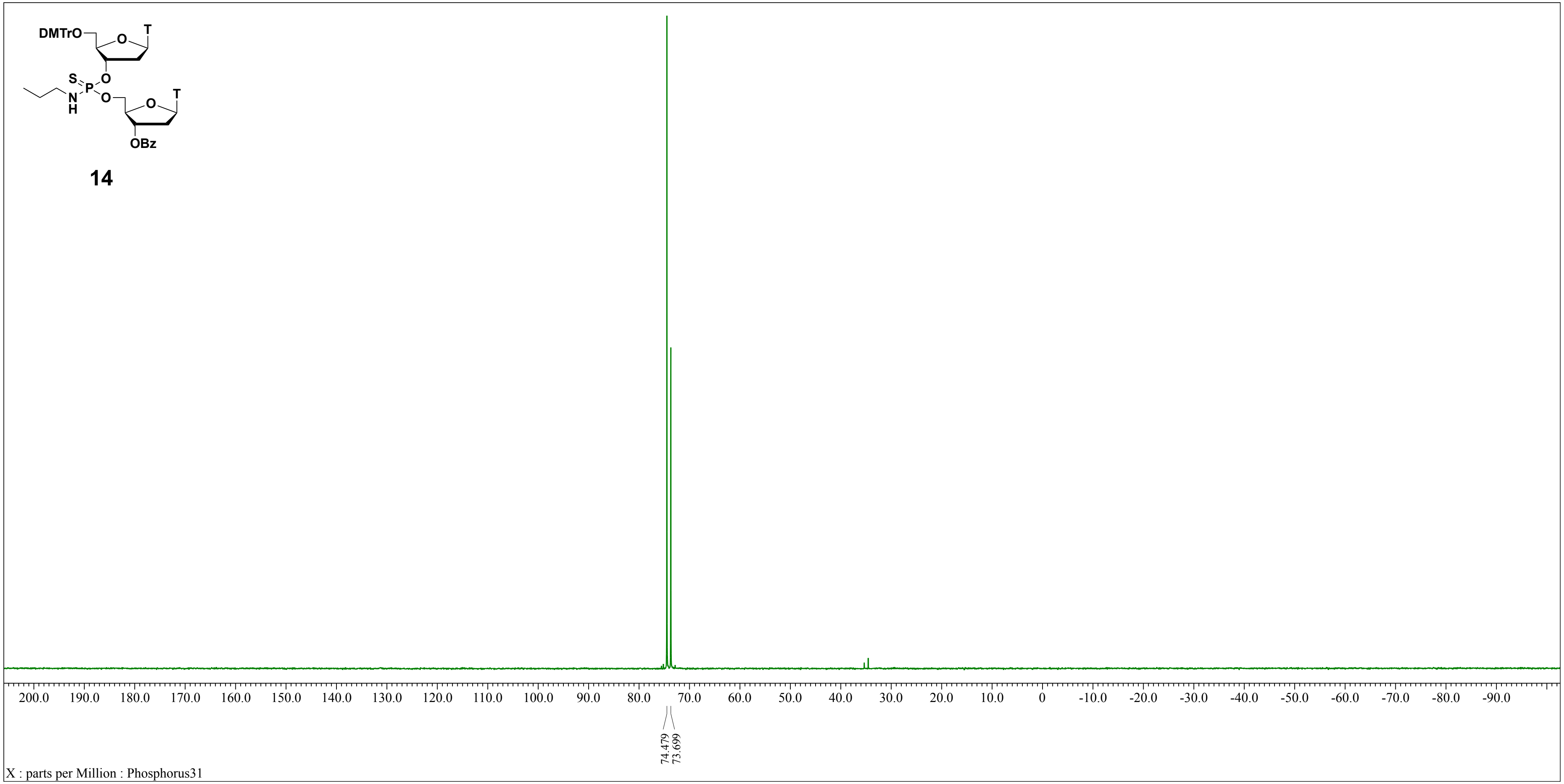

Supplement: Supplementary file 1 — jo3c00659_si_001.pdf [file jo3c00659_si_001.pdf]
